# Uptake of Cancer Genetic Services for Chatbot vs Standard-of-Care Delivery Models: The BRIDGE Randomized Clinical Trial

**DOI:** 10.1001/jamanetworkopen.2024.32143

**Published:** 2024-09-09

**Authors:** Kimberly A. Kaphingst, Wendy K. Kohlmann, Rachelle Lorenz Chambers, Jemar R. Bather, Melody S. Goodman, Richard L. Bradshaw, Daniel Chavez-Yenter, Sarah V. Colonna, Whitney F. Espinel, Jessica N. Everett, Michael Flynn, Amanda Gammon, Adrian Harris, Rachel Hess, Lauren Kaiser-Jackson, Sang Lee, Rachel Monahan, Joshua D. Schiffman, Molly Volkmar, David W. Wetter, Lingzi Zhong, Devin M. Mann, Ophira Ginsburg, Meenakshi Sigireddi, Kensaku Kawamoto, Guilherme Del Fiol, Saundra S. Buys

**Affiliations:** 1 Huntsman Cancer Institute, Salt Lake City, Utah; 2 Department of Communication, University of Utah, Salt Lake City; 3 Perlmutter Cancer Center, NYU Langone Health, New York; 4 School of Global Public Health, New York University, New York; 5 Department of Biomedical Informatics, University of Utah, Salt Lake City; 6 Veterans Administration Medical Center, Salt Lake City, Utah; 7 Department of Internal Medicine, University of Utah, Salt Lake City; 8 Department of Pediatrics, University of Utah, Salt Lake City; 9 Community Physicians Group, University of Utah Health, Salt Lake City; 10 Department of Population Health Sciences, University of Utah, Salt Lake City; 11 Department of Population Health, NYU Grossman School of Medicine, New York; 12 Center for Global Health, National Cancer Institute, Rockville, Maryland

## Abstract

**Importance:**

Increasing numbers of unaffected individuals could benefit from genetic evaluation for inherited cancer susceptibility. Automated conversational agents (ie, chatbots) are being developed for cancer genetics contexts; however, randomized comparisons with standard of care (SOC) are needed.

**Objective:**

To examine whether chatbot and SOC approaches are equivalent in completion of pretest cancer genetic services and genetic testing.

**Design, Setting, and Participants:**

This equivalence trial (Broadening the Reach, Impact, and Delivery of Genetic Services [BRIDGE] randomized clinical trial) was conducted between August 15, 2020, and August 31, 2023, at 2 US health care systems (University of Utah Health and NYU Langone Health). Participants were aged 25 to 60 years, had had a primary care visit in the previous 3 years, were eligible for cancer genetic evaluation, were English or Spanish speaking, had no prior cancer diagnosis other than nonmelanoma skin cancer, had no prior cancer genetic counseling or testing, and had an electronic patient portal account.

**Intervention:**

Participants were randomized 1:1 at the patient level to the study groups at each site. In the chatbot intervention group, patients were invited in a patient portal outreach message to complete a pretest genetics education chat. In the enhanced SOC control group, patients were invited to complete an SOC pretest appointment with a certified genetic counselor.

**Main Outcomes and Measures:**

Primary outcomes were completion of pretest cancer genetic services (ie, pretest genetics education chat or pretest genetic counseling appointment) and completion of genetic testing. Equivalence hypothesis testing was used to compare the study groups.

**Results:**

This study included 3073 patients (1554 in the chatbot group and 1519 in the enhanced SOC control group). Their mean (SD) age at outreach was 43.8 (9.9) years, and most (2233 of 3063 [72.9%]) were women. A total of 204 patients (7.3%) were Black, 317 (11.4%) were Latinx, and 2094 (75.0%) were White. The estimated percentage point difference for completion of pretest cancer genetic services between groups was 2.0 (95% CI, −1.1 to 5.0). The estimated percentage point difference for completion of genetic testing was −1.3 (95% CI, −3.7 to 1.1). Analyses suggested equivalence in the primary outcomes.

**Conclusions and Relevance:**

The findings of the BRIDGE equivalence trial support the use of chatbot approaches to offer cancer genetic services. Chatbot tools can be a key component of sustainable and scalable population health management strategies to enhance access to cancer genetic services.

**Trial Registration:**

ClinicalTrials.gov Identifier: NCT03985852

## Key Points

**Question:**

Are chatbot and standard-of-care approaches equivalent in completion of pretest cancer genetic services and genetic testing?

**Findings:**

In this equivalence trial involving 3073 patients, equivalence between service delivery models was observed for completion of pretest cancer genetic services (estimated percentage point difference, 2.0; 95% CI, −1.1 to 5.0) and completion of genetic testing (estimated percentage point difference, −1.3; 95% CI, −3.7 to 1.1).

**Meaning:**

These findings have important implications for clinical practice because they support chatbot approaches for meeting the rapidly increasing demand for genetic services in light of the limited genetic counseling workforce.

## Teaser

This randomized clinical trial examines whether chatbot and standard-of-care delivery models are equivalent in completion of pretest cancer genetic services and genetic testing among primary care patients.

## Introduction

Identifying unaffected individuals with inherited cancer susceptibility allows for genetic evaluation and implementation of individualized prevention and screening recommendations.^1,2,3,4,5,6^ However, most people with inherited cancer susceptibility are unaware of their condition.^7,8,9,10^ Additionally, more genes have been associated with inherited cancers,^11,12,13,14,15,16,17,18,19^ and broader ranges of family histories are indications for genetic testing,^20^ increasing the opportunity to identify individuals at increased risk. Once identified, at-risk individuals may face barriers to accessing genetic services due to the limited number of trained genetic specialists for direct patient care.^21,22,23^ Scalable and sustainable strategies are needed to effectively identify individuals with inherited cancer susceptibility and deliver genetic services.

Prior research has compared different delivery models for genetic services,^24^ and technology-based tools have received attention as potentially scalable approaches to service delivery.^25,26^ Interest in chatbots, automated conversational agents that use artificial intelligence and natural language processing to simulate human conversation, has grown rapidly.^26,27^ Chatbots have advantages for delivering information, including interactivity, chunking information into small segments, and allowing for choice in information received.^28,29,30,31^ In genetics contexts, chatbots have been deployed for various purposes.^32,33,34,35,36,37^ Prior research has provided support for the acceptability and feasibility of chatbots in genetics contexts^27,32,37,38^ and has shown that chatbots may facilitate family communication.^35,39^ However, few studies have examined how chatbots affect delivery of cancer genetic services,^40^ and randomized designs are needed to compare chatbots vs standard of care (SOC). This study aimed to address these research gaps.^41^

## Methods

This equivalence trial (Broadening the Reach, Impact, and Delivery of Genetic Services [BRIDGE] randomized clinical trial [RCT]) was approved as a single–institutional review board protocol by the University of Utah Institutional Review Board, consistent with current US National Institutes of Health policy for multisite RCTs. The trial protocol and statistical analysis plan are presented in Supplement 1. Because the trial compared 2 clinical service delivery models, the institutional review board approved a waiver of consent for the procedures described here. The study followed the Consolidated Standards of Reporting Trials (CONSORT) reporting guideline.

### Trial Design Overview

The BRIDGE trial was conducted between August 15, 2020, and August 31, 2023. First, the trial investigators identified unaffected primary care patients who qualified for genetic risk assessment in 2 health care systems (University of Utah Health and NYU Langone Health) using a standards-based platform to automatically evaluate cancer family history information in a patient’s electronic health record (EHR). The trial investigators then compared the primary outcomes of uptake of pretest cancer genetic services and genetic testing for chatbot vs enhanced SOC genetic services delivery models among eligible patients randomly selected from those identified by the algorithm (Figure). Participants were randomized using a random number generator 1:1 at the patient level. They were stratified by site from the primary care departments at each site, with at least 100 patients meeting the algorithm criteria. In both groups, a genetic counseling assistant (GCA) confirmed trial eligibility, sent outreach messages to primary care clinicians (PCCs) and patients, and placed genetic testing orders (as described in the Procedures subsection). The difference between groups was that in the enhanced SOC control group, patients were invited in the outreach message to complete an SOC pretest appointment with a certified genetic counselor. In the chatbot intervention group, patients were invited to complete a pretest genetics education chat. The null hypothesis of nonequivalence in the primary outcomes between groups was tested.

**Figure.**
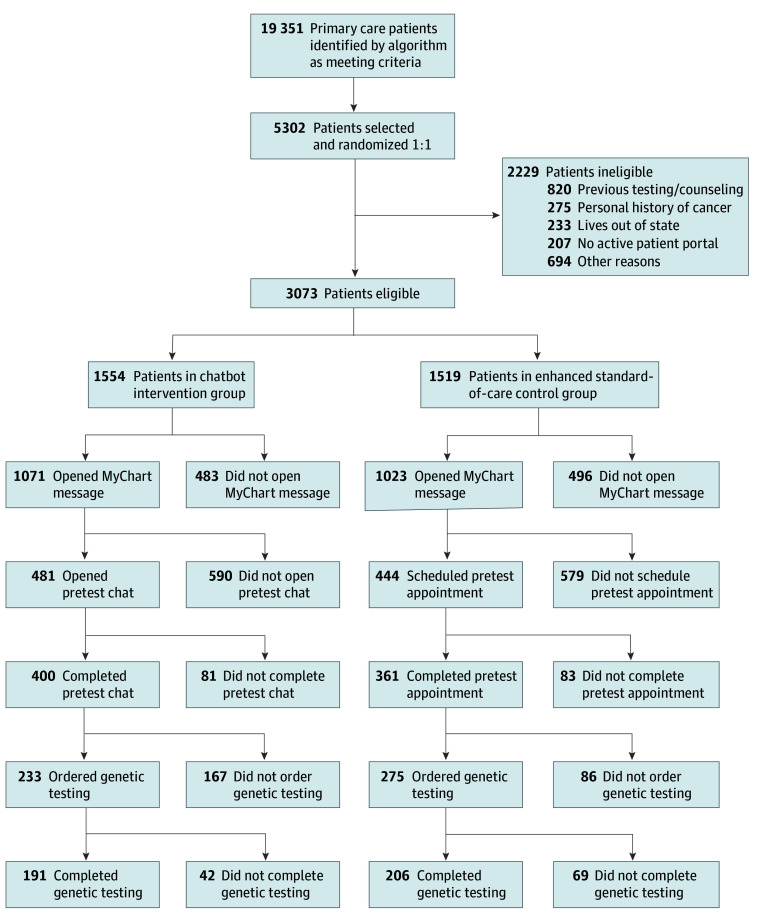
Study Flow Diagram

### Participants

We included unaffected patients aged 25 to 60 years who had a primary care visit in the University of Utah Health or NYU Langone Health systems in the previous 3 years and were eligible for cancer genetic testing according to modified National Comprehensive Cancer Network (NCCN) guidelines based on their previously obtained family history in the EHR (Epic; Epic Systems).^42,43,44^ Patients were identified using GARDE, an open-source, standards-based platform that extracts and evaluates cancer family history information available in structured EHR data elements and adds eligible patients to a registry in the EHR’s population health management tool.^42,43^ Primary care visits in internal medicine or family medicine were included at both sites; obstetrics and gynecology visits were also included at University of Utah Health. Patients were not required to have a PCC on record. Additional eligibility criteria for the trial were being English or Spanish speaking, having no prior cancer diagnosis other than nonmelanoma skin cancer, having no prior genetic counseling or testing related to hereditary cancer, and having an electronic patient portal (MyChart in Epic) account or creating one. Patients with a known variant in the family were excluded from the trial and referred for clinical genetic services.

### Procedures

Random samples of 2241 patients at University of Utah Health and 3061 at NYU Langone Health were selected from the pool of eligible patients identified by GARDE and allocated to study groups. Additional trial eligibility criteria were reviewed by a GCA before and after patient outreach. In both groups, 1 week before patient outreach, PCCs were notified that their patients would be contacted. Procedures were available in English or Spanish. Patients were not billed for pretest genetic services in either group. Genetic testing was billed as a clinical test in both groups.

#### Control Group

In the enhanced SOC control group, selected patients were first sent a patient portal message, recommending genetic services and encouraging them to contact the genetics clinic at their study site to schedule a pretest genetic counseling appointment. Nonresponders received a reminder message 1 week later and up to 2 additional follow-up telephone calls from a GCA. Interested eligible patients were scheduled for a pretest genetic counseling appointment with a certified genetic counselor. As described in the BRIDGE protocol previously,^41^ genetic counseling appointments in this group followed clinical SOC. Most were performed by telephone. Genetic testing orders were entered into the laboratory portal by a GCA, and a saliva kit, instructions, and return mailing materials were sent to patients’ home.

#### Intervention Group

In the patient-directed chatbot intervention group, selected patients were first sent a patient portal message recommending genetic services and including a hyperlink to complete pretest genetics education via chatbot. The pretest genetics education chat used the Invitae technical platform (Invitae Corporation) and chatbot interface for delivery,^45^ with content specifically scripted for the BRIDGE study by an interdisciplinary team.^31,41^ The chatbot content was developed to represent key information delivered during SOC pretest genetic counseling appointments and included text, images, and video. The first part of the chat was a video message from the lead genetic counselor at the patient’s site introducing the chat. Patients then moved through a core set of scripted information (eFigure in Supplement 2) and could request additional information on preselected topics or ask open-ended questions in a free-text format. The team scripted responses to possible open-ended questions; questions without a scripted response were emailed to the genetic counseling team. Patients with incomplete chats received a reminder message 1 week later and up to 2 follow-up telephone calls from a GCA. Patients could request to speak with a genetic counselor instead of or in addition to completing the pretest genetics education chat. At the end of the chatbot script, patients were offered the option to continue with genetic testing. A GCA contacted patients to confirm their decision about genetic testing and collect additional family history information if needed. The GCA entered the genetic testing order into the laboratory portal, and a saliva collection kit, instructions, and return mailing supplies were sent to patients’ home. Patient interactions with the chatbot, including their testing decisions, were saved in the patient EHR as a note.

#### Genetic Testing

Genetic testing was performed at Clinical Laboratory Improvement Act–certified commercial laboratories. Patients received pancancer, multigene panel tests for cancer susceptibility genes that included approximately 34 to 36 genes. The laboratory performing the testing billed the patients’ insurance. Uninsured patients were informed about free or reduced cost options.

### Measures

Data were abstracted from the population health management registry and study clinical records at each site. Outcomes and patient characteristics were assessed as follows.

#### Outcomes

The primary outcomes were defined as completion of pretest cancer genetic services (ie, completed pretest genetics education chat in the intervention group or pretest genetic counseling appointment in the control group) and completion of genetic testing. These 2 primary outcomes examined whether patients received the information needed to consider genetic testing through pretest services and whether they chose to complete testing. The secondary outcomes were starting pretest cancer genetic services (opening pretest genetics education chat in intervention group or scheduling pretest genetic counseling appointment in control group) and ordering genetic testing.

#### Patient Characteristics

We abstracted EHR data on patient age at outreach, sex, race and ethnicity, language preference, zip code, and having a recorded PCC. Race and ethnicity were included to characterize the patient populations of the 2 health care systems and are reported as Black, Latinx, White, or other race or ethnicity (American Indian, Alaska Native, Asian, Native Hawaiian, or Pacific Islander). Zip code data were used to determine urbanicity through Rural-Urban Commuting Area codes.^46^ We examined whether the patient met more than 1 NCCN criterion for cancer genetic testing.^42,43,44^

### Statistical Analysis

Descriptive statistics were calculated using the gtsummary package in R, version 4.3.0 (R Project for Statistical Computing).^47^ Pearson χ^2^, Wilcoxon rank sum, and Fisher exact tests assessed bivariate associations by experimental condition. We also computed descriptive statistics and bivariate associations stratified by study site. We used the Farrington-Manning test^48^ to test an equivalence hypothesis between study groups for the overall patient population and for each study site. The equivalence margin was set at 11 percentage points for completion of pretest cancer genetic services and 5 percentage points for completion of genetic testing based on a priori power calculations.^41^ All statistical analyses were performed using R, version 4.3.0,^49^ with *P* < .05 (2-sided) considered statistically significant.

## Results

### Participant Characteristics

Of the 5302 patients randomly selected, 3073 were eligible and included in this study (1554 in the chatbot group and 1519 in the enhanced SOC control group; Table 1). There were 1444 patients in the University of Utah Health sample (Table 2) and 1629 in the NYU Langone Health sample (Table 3). The mean (SD) age of patients at outreach was 43.8 (9.9) years. Of the 3063 patients with data on sex available, 2233 (72.9%) were female and 830 (27.1%) were male. Race and ethnicity were available for 2792 patients: 204 (7.3%) were Black, 317 (11.4%) were Latinx, 2094 (75.0%) were White, and 177 (6.3%) were of other race or ethnicity. Most patients had a recorded PCC (2360 of 3072 [76.8%]), lived in an urban area (2960 of 3071 [96.4%]), and indicated a preference for English (3028 of 3067 [98.7%]).

**Table 1.** Overall Patient Population Characteristics by Experimental Condition^a^

Characteristic	Overall (N = 3073)	Chatbot group (n = 1554)	Standard-of-care group (n = 1519)
Age, mean (SD) [range], y (n = 3071)	43.8 (9.9) [26-63]	43.5 (9.9) [26-63]	44.1 (9.9) [26-63]
Sex (n = 3063)			
Male	830 (27.1)	402 (25.9)	428 (28.3)
Female	2233 (72.9)	1149 (74.1)	1084 (71.7)
Race and ethnicity (n = 2792)			
Black	204 (7.3)	103 (7.3)	101 (7.3)
Latinx	317 (11.4)	165 (11.7)	152 (11.0)
White	2094 (75.0)	1055 (74.9)	1039 (75.1)
Other^b^	177 (6.3)	85 (6.0)	92 (6.6)
Language preference (n = 3067)			
English	3028 (98.7)	1530 (98.6)	1498 (98.8)
Non-English	39 (1.3)	21 (1.4)	18 (1.2)
No. of algorithm criteria met			
1	2873 (93.5)	1447 (93.1)	1426 (93.9)
≥2	200 (6.5)	107 (6.9)	93 (6.1)
Has a recorded primary care clinician	2360 (76.8)	1199 (77.2)	1161 (76.4)
Study site			
NYU Langone Health	1629 (53.0)	823 (53.0)	806 (53.1)
University of Utah Health	1444 (47.0)	731 (47.0)	713 (46.9)
Residence (n = 3071)			
Rural	111 (3.6)	58 (3.7)	53 (3.5)
Urban	2960 (96.4)	1496 (96.3)	1464 (96.5)
Completion of genetic testing			
No	2676 (87.1)	1363 (87.7)	1313 (86.4)
Yes	397 (12.9)	191 (12.3)	206 (13.6)
Completion of pretest genetic services			
No	2312 (75.2)	1154 (74.3)	1158 (76.2)
Yes	761 (24.8)	400 (25.7)	361 (23.8)
Ordered genetic testing			
No	2565 (83.5)	1321 (85.0)	1244 (81.9)
Yes	508 (16.5)	233 (15.0)	275 (18.1)
Beginning pretest services			
No	2148 (69.9)	1073 (69.0)	1075 (70.8)
Yes	925 (30.1)	481 (31.0)	444 (29.2)

^a^ Unless specified otherwise, values are presented as No. (%) of patients.

^b^ Includes American Indian, Alaska Native, Asian, Native Hawaiian, and Pacific Islander.

**Table 2.** University of Utah Health Patient Population Characteristics by Experimental Condition^a^

Characteristic	Overall (n = 1444)	Chatbot group (n = 731)	Standard-of-care group (n = 713)
Age, mean (SD) [range], y (n = 1442)	43.3 (9.6) [28-63]	43.3 (9.7) [28-63]	43.3 (9.5) [28-63]
Sex (n = 1441)			
Male	310 (21.5)	156 (21.4)	154 (21.7)
Female	1131 (78.5)	574 (78.6)	557 (78.3)
Race and ethnicity (n = 1412)			
Black	17 (1.2)	10 (1.4)	7 (1.0)
Latinx	156 (11.0)	85 (11.9)	71 (10.2)
White	1160 (82.2)	587 (81.9)	573 (82.4)
Other^b^	79 (5.6)	35 (4.9)	44 (6.3)
Language preference (n = 1441)			
English	1419 (98.5)	717 (98.2)	702 (98.7)
Non-English	22 (1.5)	13 (1.8)	9 (1.3)
No. of algorithm criteria met			
1	1355 (93.8)	690 (94.4)	665 (93.3)
≥2	89 (6.2)	41 (5.6)	48 (6.7)
Has a recorded primary care clinician	1007 (69.7)	522 (71.4)	485 (68.0)
Residence			
Rural	108 (7.5)	57 (7.8)	51 (7.2)
Urban	1336 (92.5)	674 (92.2)	662 (92.8)
Completion of genetic testing			
No	1239 (85.8)	627 (85.8)	612 (85.8)
Yes	205 (14.2)	104 (14.2)	101 (14.2)
Completion of pretest genetic services			
No	1050 (72.7)	526 (72.0)	524 (73.5)
Yes	394 (27.3)	205 (28.0)	189 (26.5)
Ordered genetic testing			
No	1169 (81.0)	605 (82.8)	564 (79.1)
Yes	275 (19.0)	126 (17.2)	149 (20.9)
Beginning pretest services			
No	969 (67.1)	488 (66.8)	481 (67.5)
Yes	475 (32.9)	243 (33.2)	232 (32.5)

^a^ Unless specified otherwise, values are presented as No. (%) of patients.

^b^ Includes American Indian, Alaska Native, Asian, Native Hawaiian, and Pacific Islander.

**Table 3.** NYU Langone Health Patient Population Characteristics by Experimental Condition^a^

Characteristic	Overall (n = 1629)	Chatbot group (n = 823)	Standard-of-care group (n = 806)
Age, mean (SD) [range], y	44.3 (10.2) [26-63]	43.8 (10.1) [26-63]	44.8 (10.2) [26-63]
Sex (n = 1622)			
Male	520 (32.1)	246 (30.0)	274 (34.2)
Female	1102 (67.9)	575 (70.0)	527 (65.8)
Race and ethnicity (n = 1380)			
Black	187 (13.6)	93 (13.5)	94 (13.6)
Latinx	161 (11.7)	80 (11.6)	81 (11.8)
White	934 (67.7)	468 (67.7)	466 (67.6)
Other^b^	98 (7.1)	50 (7.2)	48 (7.0)
Language preference (n = 1626)			
English	1609 (99.0)	813 (99.0)	796 (98.9)
Non-English	17 (1.0)	8 (1.0)	9 (1.1)
No. of algorithm criteria met			
1	1518 (93.2)	757 (92.0)	761 (94.4)
≥2	111 (6.8)	66 (8.0)	45 (5.6)
Has a recorded primary care clinician (n = 1628)	1353 (83.1)	677 (82.4)	676 (83.9)
Residence (n = 1627)			
Rural	3 (0.2)	1 (0.1)	2 (0.5)
Urban	1624 (99.7)	822 (99.9)	802 (99.5)
Completion of genetic testing			
No	1437 (88.2)	736 (89.4)	701 (87.0)
Yes	192 (11.8)	87 (10.6)	105 (13.0)
Completion of pretest genetic services			
No	1262 (77.5)	628 (76.3)	634 (78.7)
Yes	367 (22.5)	195 (23.7)	172 (21.3)
Ordered genetic testing			
No	1396 (85.7)	716 (87.0)	680 (84.4)
Yes	233 (14.3)	107 (13.0)	126 (15.6)
Beginning pretest services			
No	1179 (72.4)	585 (71.1)	594 (73.7)
Yes	450 (27.6)	238 (28.9)	212 (26.3)

^a^ Unless specified otherwise, values are presented as No. (%) of patients.

^b^ Includes American Indian, Alaska Native, Asian, Native Hawaiian, and Pacific Islander.

### Completion of Pretest Cancer Genetic Services and Genetic Testing

Analyses suggested equivalence between groups overall and for each site (Table 4). For the primary outcome of completion of pretest cancer genetic services, the estimated percentage point difference between groups was 2.0 (95% CI, −1.1 to 5.0). Outcomes were similar by site; estimated percentage point differences were 1.5 (95% CI, −3.1 to 6.1) for University of Utah Health and 2.4 (95% CI, −1.7 to 6.4) for NYU Langone Health. Few patients in the chatbot group who started the pretest genetics education chat requested to meet with a genetic counselor (4 [2.8%] at University of Utah Health and 4 [2.4%] at NYU Langone Health).

**Table 4.** Equivalence Tests for Primary and Secondary Trial Outcomes

Outcome by study site	Chatbot group^a^	Standard-of-care group^a^	Estimated percentage point difference (95% CI)	*Z* statistic	*P* value^b^
**Overall**					
Completion of genetic testing	191 (12.3)	206 (13.6)	−1.3 (−3.7 to 1.1)	3.05	.002
Completion of pretest genetic services	400 (25.7)	361 (23.8)	2.0 (−1.1 to 5.0)	8.28	<.001
Ordered genetic testing	233 (15.0)	275 (18.1)	−3.1 (−5.7 to −0.5)	1.41	.16
Beginning pretest services	481 (31.0)	444 (29.2)	1.7 (−1.5 to 5.0)	7.69	<.001
**University of Utah Health**					
Completion of genetic testing	104 (14.2)	101 (14.2)	0.1 (−3.6 to 3.7)	2.73	.006
Completion of pretest genetic services	205 (28.0)	189 (26.5)	1.5 (−3.1 to 6.1)	5.33	<.001
Ordered genetic testing	126 (17.2)	149 (20.9)	−3.7 (−7.7 to 0.4)	0.65	.52
Beginning pretest services	243 (33.2)	232 (32.5)	0.7 (−4.1 to 5.5)	4.74	<.001
**NYU Langone Health**					
Completion of genetic testing	87 (10.6)	105 (13.0)	−2.5 (−5.6 to 0.7)	1.58	.11
Completion of pretest genetic services	195 (23.7)	172 (21.3)	2.4 (−1.7 to 6.4)	6.39	<.001
Ordered genetic testing	107 (13.0)	126 (15.6)	−2.6 (−6.1 to 0.8)	1.36	.17
Beginning pretest services	238 (28.9)	212 (26.3)	2.6 (−1.7 to 7.0)	6.13	<.001

^a^ Values are presented as No. (%) of patients.

^b^ *P* values are based on the Farrington-Manning test for rate differences.

For the primary outcome of completion of cancer genetic testing, the estimated percentage point difference was −1.3 (95% CI, −3.7 to 1.1) (Table 4). Analyses suggested equivalence between groups overall and for University of Utah Health, but not for NYU Langone Health. The estimated percentage point differences for completion of genetic testing were 0.1 (95% CI, −3.6 to 3.7) for University of Utah Health and −2.5 (95% CI, −5.6 to 0.7) for NYU Langone Health (Table 4).

Analyses suggested equivalence between groups in the secondary outcome of beginning pretest services overall and for each study site (Table 4). There was no evidence to suggest equivalence between groups in the secondary outcome of ordering genetic testing overall and for each study site (Table 4): estimated percentage point differences were −3.1 (95% CI, −5.7 to −0.5) overall, −3.7 (95% CI, −7.7 to 0.4) for University of Utah Health, and −2.6 (95% CI, −6.1 to 0.8) for NYU Langone Health.

## Discussion

The BRIDGE trial compared uptake of cancer genetic services for chatbot vs SOC approaches among 3073 unaffected primary care patients in 2 large health care systems eligible for cancer genetic evaluation based on their family history. The trial findings suggested equivalence between these genetic services delivery models for the primary outcomes of uptake of pretest cancer genetic services and genetic testing and for the secondary outcome of beginning pretest genetic services, although statistically significantly more patients in the SOC group ordered genetic testing. The equivalence findings have important implications for clinical practice because chatbot approaches are supported to offer pretest cancer genetic services and genetic testing after outreach to unaffected patients eligible for genetic evaluation, providing a way to meet the rapidly increasing demand for these services. Few patients in the chatbot group requested a clinical genetic counseling appointment, suggesting acceptability of the chatbot approach.

A chatbot can provide multiple advantages as a technology-based approach to expand access to genetic services because it is highly scalable, private, and cost-effective and can be used at places and times of individuals’ choosing.^26,41,50^ Chatbots could be used to conduct routine tasks, allowing genetic counselors to focus on more specialized care,^51,52^ and to facilitate a more personalized counseling approach meeting patients’ individual needs.^25^ In the BRIDGE trial, the pretest genetics education chatbot achieved these goals by providing a core set of information to all patients while allowing some degree of personalization through selection of additional information or asking open-ended questions. A prior analysis of use of the chatbot in a pilot phase showed that most patients selected a limited amount of additional information and few asked open-ended questions, suggesting that the content generally met patients’ educational needs.^31^ This observation is supported by a small RCT of women with stage 0 to III breast cancer not meeting NCCN criteria for genetic testing, in which investigators found similar knowledge and satisfaction among those randomized to pretest counseling via chatbot compared with a certified genetic counselor.^40^ The BRIDGE study team has created an open-source version of the chat, enhancing the scalability of this approach in other health care systems.

The trial findings indicate that a chatbot could be part of what has been described as a mainstreaming model, with standardized pretest education delivered outside of traditional genetic counseling.^32,53^ Chatbots may support pretest genetics education without individualized genetic counseling for many patients. However, increasing reach and engagement is important, and others have highlighted the need to increase uptake of digital approaches.^54^ Although the completion of pretest genetic services observed in the BRIDGE trial is consistent with prior population screening efforts,^54,55,56,57^ most patients did not complete pretest genetic counseling or testing in either service delivery model. Optimizing patient engagement is critical. It will be important for future research to determine what aspects of cancer genetic counseling can be effectively delivered via chatbot, particularly as this tool becomes more personalized and empathic in its responses.^58^ Approaches that limit clinician and staff contacts to those essential to improving patient outcomes will be more scalable at a population level. The MAGENTA (Making Genetic Testing Accessible) trial compared 4 different approaches with or without individualized pretest and posttest genetic counseling.^59^ The MAGENTA investigators found the highest rates of testing completion in the 2 groups without pretest genetic counseling,^59^ which suggests that studies that compare outcomes of chatbot use to approaches without pretest education are also important. Patient cognitive and emotional responses, such as decision regret, to different service delivery models should also be investigated.

The findings of this study can inform the use of chatbots in cancer care more generally. A systematic review of 21 studies of chatbot applications across the cancer continuum provided some support for patient satisfaction and efficacy.^60^ Few prior studies have conducted randomized comparisons with SOC or examined service delivery outcomes.^60,61^ The present findings provide support for expanded use of chatbots. Chatbots may be a viable approach to address underidentification of individuals with inherited cancer syndromes through genetic risk assessment of unaffected individuals. Traditional strategies for identifying patients with inherited cancer susceptibility start with the person with cancer, prompting cascade testing of at-risk relatives if a pathogenic variant is identified.^62^ However, testing of patients with cancer is low and the majority of people who harbor pathogenic variants associated with cancer risk are not aware,^7,8,9,10^ underscoring the need for efficient and scalable approaches for identifying those at increased risk.^10,25,63^ A recent systematic review identified 6 studies with chatbots used to perform genetic cancer risk assessment and found a pooled estimated completion rate for risk assessment of 36.7% (95% CI, 14.8% to 65.9%).^64^ Completion rates may vary by factors such as population or affected status. Also, in several studies, chatbots were offered to patients in conjunction with medical appointments, indicating that timing and integration with other health care services may be important. Notably, none of these studies involved a comparison, highlighting the need for randomized designs. In addition, although the present trial focused on unaffected patients, automated approaches may be worth exploring to address the lack of genetic testing in patients with cancer.^65^ Future studies can also examine factors affecting adoption and implementation of chatbots in cancer genetics care.^66,67^

### Limitations

The trial findings should be considered in light of the following limitations. Genetic testing procedures were not fully automated for patients randomized to the chatbot group; to enhance patient safety in the trial, GCAs contacted all patients in the chatbot group to confirm testing decisions. The genetic counseling teams were not blinded to study group since the EHR indicated whether pretest services had been delivered via chatbot or an appointment. Most participants were White and female, and our prior analyses showed that use of EHR data introduced bias into the population identified as eligible for testing.^68^ Despite the availability of trial procedures in Spanish, few Spanish-speaking patients met the study criteria^68^ or completed genetic services and testing. Intentional approaches may be needed to deliver genetic services to these populations. All trial participants were affiliated with University of Utah Health or NYU Langone Health, had a primary care visit within the last 3 years, and had access to the patient portal. It will be important to expand the reach of genetic testing, with or without chatbots, among patients who are uninsured or face other access barriers. Future studies can also examine how usage of patient portal accounts affects responses to outreach for genetic services. Because we received a waiver of consent for clinical procedures in the trial, we were not able to collect baseline information about patients beyond what was available in the EHR. However, this pragmatic design allowed us to contact a random sample of eligible primary care patients rather than a highly selected group who agreed to participate in a research study.

## Conclusions

Despite this trial’s limitations, its findings advance the state of the science in delivery of cancer genetic services to unaffected patients meeting criteria for genetic evaluation. By comparing chatbot service delivery vs SOC using a multisite RCT design, the findings of the BRIDGE equivalence trial support the use of chatbot approaches to offer cancer genetic services. As referrals for cancer genetic services increase,^24,69,70,71^ models that direct genetic counseling time and resources to those patients with the greatest needs are warranted.^63^ The findings show that use of chatbots to deliver pretest genetic services has strong potential to increase access to these services for unaffected patients. These trial findings therefore support the implementation of systemwide population health management strategies to deliver cancer genetic services.

## Notes

Supplement 1.
Trial Protocol and Statistical Analysis Plan

Supplement 2. **eFigure.** BRIDGE Pretest Genetics Education Chat for the University of Utah Health Site

Supplement 3.
Data Sharing Statement
