# Supplementary material for: Uptake of Cancer Genetic Services for Chatbot vs Standard-of-Care Delivery Models: The BRIDGE Randomized Clinical Trial
Source: JAMA Netw Open. 2024 Sep 9;7(9):e2432143. doi: 10.1001/jamanetworkopen.2024.32143 (PMC11385050; doi:10.1001/jamanetworkopen.2024.32143)
Supplement: Supplement 1. — Trial Protocol and Statistical Analysis Plan [file jamanetwopen-e2432143-s001.pdf]

Supplemental Online Content.

Supplement 1. Trial protocol submitted to Institutional Review Board.

The full trial protocol is available at:

Kaphingst KA, Kohlmann W, Chambers RL, et al. Comparing models of delivery for cancer genetic services among patients receiving primary care who meet criteria for genetic evaluation in two healthcare systems: BRIDGE randomized controlled trial. *BMC Health Sciences Research*. 2021;21:542. doi:10.1186/s12913-021-06489-y

Electronic
RESEARCH INTEGRITY AND COMPLIANCE
THE UNIVERSITY OF UTAH

Date: Tuesday, August 6, 2024 10:49:19 AM

Print Close

IRB_00123563 **Created:** 6/5/2019 3:01 PM

PI: Kimberly Kaphingst ScD **Submitted:** 3/5/2019

Title: Broadening the Reach, Impact, and Delivery of Genetic Services

IRB_00123563
1. Contacts and Title

1. Study Introduction

1. **Responsible Investigator:**
Kimberly Kaphingst

Email	Training	CoI Date
kim.kaphingst@hci.utah.edu	4/25/2022 SMCG	3/19/2024

a. **Position of the Investigator:**

- (●) **Faculty or** Non-Academic Equivalent
- ( ) Student
- ( ) Staff
- ( ) Resident/Fellow
- ( ) Other

2. **Contact Persons for the Responsible Investigator:**

Name	Email	Training
Kadyn Kimball	kadyn.kimball@hsc.utah.edu	2/22/2023 MCG
Kelsey Knight	Kelsey.Knight@hsc.utah.edu	3/4/2024 SMCG
Wendy Kohlmann	wendy.kohlmann@hci.utah.edu	3/21/2022 SMCG
Lindsey Martineau	lindsey.martineau@utah.edu	1/9/2023 SMCG

3. **Guests of the Responsible Investigator:**

Last Name	First Name	E-Mail
Berry	Therese	therese.berry@hci.utah.edu
Intveld	Andrew	Andrew.Intveld@hci.utah.edu
Ma	Debra	deb.ma@hci.utah.edu
Mathis	Elizabeth	elizabeth.mathis@hsc.utah.edu
Sama	Neetha Reddy	Neetha.Sama@hci.utah.edu

4. **What type of application is being submitted?**
New Study Application (or Amendment/Continuing Review)

5. **Title Of Study:**

Broadening the Reach, Impact, and Delivery of Genetic Services

6. **Study Purposes and Objectives:**

The proposed study will employ an implementation science framework to compare two models of genetic services delivery for 1,920 patients in a randomized controlled trial with clinic-level randomization. We hypothesize that uptake of genetic testing (Study Aim 2) and adherence to management recommendations (Study Aim 3) will be equivalent between the genetic services delivery models. We propose the following Specific Aims:

Aim 1) Identify primary care patients who qualify for genetic risk assessment in two healthcare systems using a CDS infrastructure to automatically evaluate family history.

*This aim has already been studied in IRB #105797. We will be utilizing this tool that is now available. Aim 1 will not be analyzed in this protocol but is part of our overall research model.

Research Aims to be studied in this protocol:

Aim 2) Compare uptake of genetic testing for patient-directed standard of care vs. enhanced standard of care genetic services delivery models for 1,920 unaffected primary care patients. We will utilize a randomized trial design with clinic-level randomization to compare two standard of care models: a *patient-directed standard of care* model and an *enhanced* model. In the *patient-directed* model those identified by the clinical decision support (CDS) infrastructure as meeting testing criteria will be informed of their cancer risks, provided with educational resources, and offered the option to select genetic testing through a patient portal. This online portal will be overseen by HCI genetic counselors. The *enhanced standard of care* model in which primary care providers and patients are notified through CDS when criteria are met (the *enhancement* of standard of care) and of the availability of standard of care genetic services. We will compare uptake of genetic testing by model and by race/ethnicity and rurality.

Aim 3) Compare adherence to recommendations and other patient responses for the genetic services delivery models. We will compare the effects of the genetic services delivery models on adherence to management recommendations at 12 months after return of results, or last genetic services contact. We will assess cognitive (i.e., recall, comprehension, uncertainty, risk perceptions) and affective (i.e., test-related distress, positive reactions, decision regret) responses at 4 weeks, and communication (i.e., family, provider) interactions at 4 weeks and 12 months after last genetic services contact. We will also examine differences in effects by race/ethnicity and rurality.

7. **Is this a multi-site study, where more than one site needs IRB approval?**

● **Yes** ○ No

8. **Background and Introduction:**

Identifying unaffected individuals with inherited cancer susceptibility is critical to risk reduction and clinical management. Hereditary cancer syndromes affect risks of many common, adult-onset cancers.[1-5] Pathogenic variants (i.e., variant that increases cancer risk; PVs) in cancer predisposition genes can be identified in 7% of breast, 11%-15% of ovarian,[2,6-8] 10% of colorectal,[3,9,10] 4%-11% of pancreas,[11] and 12% of advanced prostate cancers.[12,13] Prior evidence supports individualizing screening and prevention based on cancer risk,[14-19] such as more frequent or earlier screening or chemoprevention, and identification of a PV may allow for targeted intervention and treatment if cancer occurs.[20-23]

Advances in technology are identifying more families with an inherited cancer susceptibility. Next generation sequencing-based testing of panels of multiple genes for hereditary cancer (i.e., multigene panel) can more than double the rate of mutation detection compared to testing single genes.[24-27] A growing number of genes have been associated with increased risks for breast, ovarian, pancreas, and prostate cancers,[7,11,13,28-30] and multiple genes, in addition to Lynch syndrome, have been identified in association with colorectal cancer risk.[10,31,32] Newer sequencing technologies are also expanding the tumor spectrum associated with particular genes, leading to a broader range of family histories being considered as an indication for genetic testing. For example, family history of prostate and pancreas cancer are being incorporated into testing criteria for homologous DNA repair pathway genes (e.g., *BRCA1/2*), criteria that were previously restricted to only breast and ovarian cancer.[33] Healthcare systems are challenged by the clinical management of the increasing number of patients who qualify for these services.[19,34]

There is a need to expand beyond cancer patient populations. Efforts to identify individuals with inherited cancer susceptibility are still generally focused on cancer patients with testing of at-risk relatives recommended once a PV is found.[35-37] However, this strategy misses families in which there is no living, affected relative to test, and may be an important barrier to accessing genetic services. One report found that 50% of unaffected patients presenting for clinical genetic counseling who received a recommendation for a relative to be tested first were lost to follow-up.[38] Studies have begun to explore the feasibility of screening primary care patients for inherited cancer susceptibility.[39-42] National guidelines recommend testing for unaffected individuals with a significant family history,[43,44] and the Affordable Care Act mandated coverage of genetic testing for hereditary breast/ovarian cancer for unaffected individuals with appropriate family history.[45] However, these initiatives have not yet been systematically implemented into primary care practice and it is unclear to what extent the need for genetic testing for hereditary cancer in unaffected individuals is being met. The National Health Interview Survey reported that only 9.5% of unaffected women with a family history of breast and ovarian cancer had discussed genetic testing with their healthcare provider, and only 2.7% had genetic testing.[46] Strategies to identify unaffected individuals across healthcare systems are therefore needed.

Family history can stratify patients' cancer risk but is underutilized in primary care. While debate continues over population-wide cancer genetic testing,[47-50] this strategy has not yet been widely implemented outside of research contexts, and often only in specific populations.[51-54] Family history can be used effectively to identify those most likely to carry a PV and to tailor screening regardless of whether a PV is identified.[55-61] However, although some family history is generally collected in primary care, the information is underutilized to stratify risk,[62-64] leading to missed prevention opportunities. In recent research, 37% of newly diagnosed breast cancer patients found to have PVs had a family history that warranted genetic testing even prior to their diagnosis.[65] Commonly reported

barriers to collection and use of family history information in clinical practice include limited visit time, competing demands, reimbursement criteria, and providers' training, knowledge and skills.[63,66-70] To address these barriers, over 75 tools have been developed[71-76] for patients to collect their family history information prior to a clinic visit to improve discussions with primary care providers (PCPs).[63,64,66,67,77-80] However, many of these tools require significant time and resources, were developed independent of workflow standards in clinics, and widespread implementation of these tools has not occurred.[64] Therefore, it is critical to develop novel strategies to capitalize on family history information already being collected in the EHR, regardless of the method, and pair it with automated tools for evaluation and stratification of risk.

Clinical decision support (CDS) tools and electronic health record (EHR) technology can utilize available family history information. System-level strategies have been suggested to assist PCPs in providing genetic services for patients.[81] EHR technology, together with CDS tools, are promising as systematic approaches to address many of the PCP time, knowledge, and skills barriers that limit the use of available family history information. CDS entails providing providers and patients with pertinent knowledge and/or person-specific information, intelligently filtered or presented at appropriate times, to enhance health and healthcare.[82] While CDS interventions have not always improved clinical care, we and others have identified best practices for optimizing effectiveness.[83,84] In prior studies, CDS tools have increased the efficiency of a hereditary cancer service,[85] improved referral patterns for patients at high risk for familial cancers,[86] and enhanced identification of Lynch syndrome among newly diagnosed colon cancer patients.[87]

Prior research comparing approaches for delivering cancer genetic services is limited. Once at-risk individuals are identified, genetic services (e.g., genetic counseling, genetic testing, risk-reduction recommendations) need to be delivered. The number of trained genetic specialists is limited with more sustainable approaches needed to accommodate an increasing patient volume.[88] Prior studies have compared mode of delivery (i.e., in-person vs. telephone) based on a traditional service model, with patient contact by a genetic counselor before and after genetic testing, examining outcomes such as uptake of genetic testing, adherence to follow-up, and patient responses (e.g., anxiety).[89-91] However, few studies have involved PCPs or explored supplementing genetic counselors with other models (i.e., computer-based pre-test education[92,93]) within the healthcare system. Implementation research studies[94] are urgently needed to compare the outcomes in real-world settings of different models to genetic services delivery, particularly models that direct genetic counseling resources to those patients with the greatest needs for genetic services.

Potential of patient-directed models of genetic services delivery has not been explored. Genetic testing is now available direct-to-consumer (DTC), including testing with multi-gene cancer panels, with no or minimal involvement of a clinician or genetic counselor.[95] Advocates believe DTC approaches increase access to genetic testing, particularly for patients in areas with few genetic services. However, it is unknown whether DTC genetic testing will result in appropriate management.[96] Clinicians need to be aware of the results to incorporate them into care, but a study of early adopters found that only 27% of patients had shared DTC results with their PCPs.[97] In addition, PCPs may have limited knowledge about how to use the results to individualize care.[98] Healthcare systems have a vested interest in ensuring that information as significant as genetic testing be part of the EHR in a meaningful way.[99] Therefore, there is significant need to develop genetic services delivery strategies that incorporate features of the DTC approach (e.g., patient-directed, increased access) but within the healthcare system and with support for providers interpreting these results.

Need to address disparities in access and use of genetic services across population subgroups. Prior research has often shown that individuals from racial and ethnic minority groups have decreased access to and utilization of genetic services,[100] even when cost barriers are minimized.[101] These disparities have been attributed to individual-level factors (e.g., awareness, knowledge, attitudes)[102-104] and system-level factors (e.g., insurance, access to specialists, lower trust).[103,105] One study with women diagnosed with breast cancer showed a strong desire for genetic testing among Latinas, but also that minority patients had more unmet needs for discussion of testing with providers.[106] Prior work has also indicated the importance of examining how race and ethnicity impact the delivery of genetic services. A randomized trial comparing in-person vs. telephone-based genetic counseling found that the effect of mode of delivery differed by race; minority women assigned to telephone counseling were the least likely to complete genetic testing.[107]

Even less research has examined access and use of genetic services by rurality (rural/frontier vs. urban). Rurality is a critical issue for Utah; 96% of the landmass is defined as rural (<100 persons per square mile) and 70% as frontier (<7 persons per square mile).[108] Individuals in rural areas in the U.S. have less access to care[109] and worse health outcomes.[110,111] Rural counties have higher incidence of cancers potentially preventable through screening.[112,113] Some disparities in cancer mortality are likely due to barriers to accessing health services in rural areas.[113] Clinical sites in rural areas may not have physicians knowledgeable about genetics.[114] In addition, in rural/frontier regions, clinics and hospitals can be hundreds of miles from patients' homes, so rural/frontier residents have less access to specialty genetics care.[70,115]

Citations can be found in the appropriate Documents & Attachments section.

IRB_00123563 **Created:** 6/5/2019 3:01 PM

IRB_00123563
2. Study Location and Sponsors

PI: Kimberly Kaphingst ScD **Submitted:** 3/5/2019

Title: Broadening the Reach, Impact, and Delivery of Genetic Services

2. Study Location and Sponsors

1. **Add all locations applying for approval of research via the University of Utah IRB or Human Research Protection Program (HRPP).**

   Click the appropriate button(s) below to add locations:

	Site Name	Investigators Name	Covered Entity	Sub Sites
View	NYU School of Medicine	Ophira Ginsburg	No	
View	University of Utah	Kimberly Kaphingst	Yes	

   a. **Select the lead site. Select N/A if there is no lead site.**
   University of Utah
   ☐ **N/A**

2. **Will a Central IRB (CIRB) or Single IRB (SIRB) model be used for review of this study for the sites listed in this application?**
   ● **Yes** ○ No

   a. **Provide the name of the organization providing CIRB/SIRB review:**
   University of Utah

3. **Indicate the source(s) of funding obtained or applied for to support this study.**

	Sponsor	Sponsor Type	Sponsor Contact Information	Prime Sponsor	Prime Sponsor Type	OrgID
View	NIH NATIONAL CANCER INSTITUTE	Federal Government	NCI Grants Management Specialist: Kimery Griffin<br>Email: griffink3@mail.nih.gov<br>Phone: 240-276-6315			11259

4. **Does this study have functions assigned to a Contract Research Organization (CRO)?**
   ○ Yes ● **No**

5. **Does this study involve use of the Utah Resource for Genetic and Epidemiologic Research (RGE)?**
   Examples: Utah Population Database (UPDB), Utah Cancer Registry (UCR), All Payers Claims Database (APCD), etc.
   ○ Yes ● **No**

IRB_00123563 **Created:** 6/5/2019 3:01 PM

IRB_00123563 View/Edit

PI: Kimberly Kaphingst ScD **Submitted:** 3/5/2019

Title: Broadening the Reach, Impact, and Delivery of Genetic Services

Addition of a Site

1. **Site Name:**
   NYU School of Medicine

2. **Site Principal Investigator**
   ☐ **Mark if Same as Responsible Investigator (syncs with investigator on the first page)**

   If the Site Principal Investigator does not have an ERICA account, fill out their name:
   Ophira Ginsburg

   Email:

   a. **Position of the Site Principal Investigator**
   Faculty or Non-Academic Equivalent

3. **Site Contact Persons, if different from the Site PI:**
   ☐ **Mark if Same as Contacts for Responsible Investigator (syncs with contacts on the first page)**

Name	Email	Training
There are no items to display		

4. **Site Guests:**

Name	Email	Training
There are no items to display		

5. **Select HIPAA coverage for this study:**
   Study procedures will be conducted outside a HIPAA Covered Entity at this site (HIPAA Privacy Rule does not apply)

6. **Select the study procedures that will be conducted at this site:**
   - Recruitment
   - Consent/Enrollment
   - Research observation/intervention with participants
   - Data collection
   - Data analysis

   Do you have an enrollment goal or anticipated enrollment number for this site?

   ○ Yes
   ○ No

7. **Add any additional sites that are part of this performance group**
   There are no items to display

IRB_00123563 **Created:** 6/5/2019 3:01 PM

IRB_00123563 View/Edit

PI: Kimberly Kaphingst ScD **Submitted:** 3/5/2019

Title: Broadening the Reach, Impact, and Delivery of Genetic Services

Addition of a Site

1. **Site Name:**
   University of Utah

2. **Site Principal Investigator**
   ☑ **Mark if Same as Responsible Investigator (syncs with investigator on the first page)**
   Kimberly Kaphingst

Email	Training	CoI Date
kim.kaphingst@hci.utah.edu	4/25/2022 SMCG	3/19/2024

   a. **Position of the Site Principal Investigator**
      Faculty or Non-Academic Equivalent

   b. **Will the Site PI consent participants?** ○ Yes ● **No**

3. **Site Contact Persons, if different from the Site PI:**
   ☑ **Mark if Same as Contacts for Responsible Investigator (syncs with contacts on the first page)**

Name	Email	Training
Kadyn Kimball	kadyn.kimball@hsc.utah.edu	2/22/2023 MCG
Kelsey Knight	Kelsey.Knight@hsc.utah.edu	3/4/2024 SMCG
Wendy Kohlmann	wendy.kohlmann@hci.utah.edu	3/21/2022 SMCG
Lindsey Martineau	lindsey.martineau@utah.edu	1/9/2023 SMCG

4. **Site Staff and Sub-Investigators**

Name	Email	Training	Obtaining Consent	CoI Date
Jo Anson	jo.anson@hci.utah.edu	3/7/2022 MCG	☑	6/13/2024
Kenneth Boucher	ken.boucher@hci.utah.edu	11/2/2022 MCG	☐	7/11/2024
Saundra Buys	saundra.buys@hci.utah.edu	6/26/2024 MCG	☐	5/23/2024
Sarah Colonna	Sarah.Colonna@hsc.utah.edu	10/4/2021 MCG	☐	5/17/2024
Whitney Espinel	Whitney.Espinel@hci.utah.edu	11/14/2022 MCG	☑	4/12/2024
Amanda Gammon	amanda.gammon@hci.utah.edu	8/19/2021 MCG	☑	11/7/2023
Kinley Garfield	kinley.garfield@hci.utah.edu	C	☑	5/2/2023

Name	Email	Training	Obtaining Consent	CoI Date
Samantha Greenberg	samantha.greenberg@hci.utah.edu	12/6/2023 MCG	☑	12/15/2023
Kelsi Hagerty	kelsi.hagerty@hci.utah.edu	C	☑	6/12/2019
Priyanka Kanth	Priyanka.kanth@hsc.utah.edu	8/16/2022 MC	☐	5/3/2023
Kensaku Kawamoto	kensaku.kawamoto@utah.edu	1/19/2024 MCG	☐	6/28/2024
Kadyn Kimball	kadyn.kimball@hsc.utah.edu	2/22/2023 MCG	☑	5/2/2023
Wendy Kohlmann	wendy.kohlmann@hci.utah.edu	3/21/2022 SMCG	☑	7/17/2024
Cathryn Koptiuch	cathryn.koptiuch@hci.utah.edu	C	☑	1/26/2022
Ryan Mooney	u0576405@utah.edu	3/20/2023 MC	☑	5/26/2023
Anne Naumer	anne.naumer@hci.utah.edu	9/26/2023 MCG	☐	2/7/2024
Joshua Schiffman	Joshua.Schiffman@hci.utah.edu	5/18/2024 MCG	☐	7/25/2024
Katie Tobik	katie.tobik@hci.utah.edu	C	☑	3/6/2019
Jennie Vagher	Jennie.Vagher@hci.utah.edu	1/3/2022 MCG	☑	6/12/2024
David Wetter	david.wetter@hci.utah.edu	1/11/2023 MCG	☐	4/27/2024

5. **Site Guests:**

Name	Email	Training

There are no items to display

6. **Select HIPAA coverage for this study:**
Study procedures will be conducted within a HIPAA Covered Entity at this site (HIPAA Privacy Rule applies)

7. **Select the study procedures that will be conducted at this site:**

Recruitment

Consent/Enrollment

Research observation/intervention with participants

Data collection

Data analysis

Do you have an enrollment goal or anticipated enrollment number for this site?

◯ Yes
◯ No

8. **Select the University of Utah department responsible for this research:**

HUNTSMAN CANCER INSTITUTE

9. **Add any additional sites that are part of this performance group**
   There are no items to display

IRB_00123563 **Created:** 6/5/2019 3:01 PM

PI: Kimberly Kaphingst ScD **Submitted:** 3/5/2019

Title: Broadening the Reach, Impact, and Delivery of Genetic Services

Sponsor Information

Please review these previously entered fields as you fill out the new form:

Sponsor: NIH NATIONAL CANCER INSTITUTE
Contact: NCI Grants Management Specialist: Kimery Griffin Email: griffink3@mail.nih.gov Phone: 240-276-6315
Grant Number: 1U01CA232826
Awardee: University of Utah (lead) and NYU Principal Investigators: Saundra Buys and Kimberly Kaphingst (Utah); Ophira Ginsburg (NYU)
Effective Date Start: 9/1/2018
Effective Date End: 8/31/2023

a. **Are you receiving award or contract management for the sponsored funds through the University of Utah Office of Sponsored Projects?**

● **Yes** ○ No

If yes, select the associated OSP Proposal ID/DSS through eAward to link it to the ERICA system.

You must have a fully approved Proposal ID/DSS number through eProposal which will show up in eAward after OSP has integrated the ID. To access the eAward application, use the instructions on the OSP website.

Link to a Proposal ID/DSS through eAward

3. Participants

1. **Ages of Participants:**

   18 and older (Consent form needed)

2. **Specific age range of participants (e.g., 7-12 years old, 60+, etc.):**
   25-60 years old

3. **Indicate any vulnerable participant groups (other than children) included:**

   None

   If "Other", please specify:

   If "None" and no children are involved, answer the following question.
   Has the participant selection process overprotected potential subjects who are considered vulnerable so that they are denied opportunities to participate in research?
   ◯ Yes ● **No**

4. **Number of participants to be included and/or enrolled in this entire study, across all study locations:** 1,920 consent cover letters sent; up to 20,000 EHR records

   At Utah prior to October 2019: 960 consent cover letters sent; up to 20,000 EHR records

5. **Characteristics of Participants/Inclusion Criteria:**

**Adapted Criteria for Genetic Testing for Inherited Cancer Susceptibilities**
First Degree Relative or Second Degree Relative diagnosed with the following regardless of age: Ovarian Cancer, Pancreas Cancer
First Degree Relative or Second Degree Relative diagnosed with the following <50 years of age: Breast Cancer, Colorectal Cancer, Endometrial Cancer
Three or more relatives on the same side of the family diagnosed with the following clusters of cancer regardless of age:
Breast Cancer, Ovarian Cancer, Pancreas Cancer, Prostate Cancer
Colorectal Cancer, Endometrial Cancer, Ovarian Cancer, Pancreas Cancer, urinary tract, brain, small intestine
Melanoma, Pancreas Cancer
Ashkenazi Jewish ancestry and family history of Breast Cancer, Ovarian Cancer, Pancreas Cancer, Prostate Cancer

Inclusion Criteria:

Criteria will be simplified to focus on first- and second-degree relatives, specific cancer type, number of family members, and age at diagnosis (**See Table**)

The algorithms to stratify patients' risk for this study will be based on National Comprehensive Cancer Network (NCCN) criteria for testing for genes in the homologous DNA repair pathway associated with breast, ovarian, prostate, and pancreas cancer and Lynch syndrome, and international consensus recommendations for genetic testing for hereditary melanoma.

Participants will be eligible to participate if they speak English.

Future possible amendment will to be include Spanish speaking patients.

6. Participant Exclusion Criteria:

Exclusion Criteria:

We will exclude patients who have a prior cancer diagnosis, other than non-melanoma skin cancer, and/or prior genetic counseling or testing related to hereditary cancer.

We will also exclude patients who do not have access to internet and therefore unable to access the patient portal.

7. Is a substantial percentage of the participant population anticipated to be non-English speaking?

○ Yes ● **No**

4. Study Information

1. **Design of Study (select all that apply):**

☐ **Non-Experimental and/or Descriptive Research Design:**

There are no items to display

☐ **Experimental and/or Interventional Research Design:**

Survey/Questionnaire Research
Randomized Trial

☐ **Development of a research resource (repositories, databases, etc.)**

There are no items to display

☐ **Other**

2. **Does your study involve the use of any placebo?**
○ Yes ● **No**

3. **Length of entire study, from initiation through closeout:**
5 Years

4. **How will participants be recruited or identified for inclusion in the study?**
   a. **Select all methods that will be used:**
   In-person contact (e.g., patients, students, etc.)
   Written or electronic record review

   b. **Describe the recruitment/participant identification process in detail (e.g. who will review charts or records, who can refer participants to the study, where will flyers be posted, how often will recruitment letters be sent, when will follow-up phone calls be made, etc.):**

   Using the EHR algorithms, individuals will be identified as meeting any genetic testing criterion. The patients will be flagged and forwarded through the electronic health record (EHR) as a work list to a genetic counseling assistant (GCA). The flag will include what criteria were reported. Eligible patients will receive an initial contact attempt in an email through the patient portal. If there is no response within a week, the patient will be resent the email through patient portal, followed by a letter to their home address, and 3 follow-up phone calls. Patients who complete genetic counseling will be sent the consent cover letter and the follow-up questionnaires.

5. **How will consent be obtained?**
Informed Consent Process (with or without a document)
Waiver or Alteration of Informed Consent

6. **Describe all the procedures chronologically, from screening/enrollment through study closeout, which will be completed in the research project.**

Clinical Standard of Care Procedures

Patient Screening

Patients meeting National Comprehensive Cancer Network guidelines for referral for genetic services will be identified using the screening tool developed and implemented in the EPIC EHR (Study Aim 1). The primary care providers (PCPs) of eligible patients will be notified of their patient's eligibility one week prior to patients being notified of the recommendation for genetic services. Patients will then be referred to participate in standard of care genetic services, with the clinical procedures depending on the study arm to which the clinic was randomized: enhanced standard of care or patient-directed standard of care.

Patients meeting criteria will be notified of the recommendation through the patient portal (i.e. MyChart). Those who do not have MyChart will be notified by mail. Those notifications will be followed by standard scheduling procedures. Patients who do not have MyChart will be asked to sign up for MyChart when they are contacted. Those who were not actively using MyChart will be provided with a refresher on the use of MyChart since this project focuses on using this tool to deliver genetic services more effectively.

Patients who do not have access to the internet or are unwilling or able to use MyChart will be directed to in person genetic counseling services. A waiver of consent will be requested to track the individuals who are not able to participate through MyChart, but they will not be asked to complete study questionnaires.

Study Arms – Clinic Randomized

Enhanced Standard of Care

This arm is referred to as enhanced since clinical decision support (CDS) tools are used to identify patients meeting guidelines rather than relying solely on provider identification of patients. Patients in this arm will have an in-person or telephone pre-test genetic counseling visit. Pre-test counseling includes review of the family history, risk assessment, discussion of the purpose of genetic testing and possible outcomes, implications for insurance coverage, costs, and assessment of psychosocial needs.

Patients can opt to proceed with genetic testing during this session. Consent for testing will be documented and a sample can be collected at that time or scheduled at another time that is convenient for the patient. All test results are returned by a genetic counselor by phone or in person based on patient preference per clinic protocol. A copy of the results and letter with tailored screening recommendations are returned to the PCP and patient via the patient portal (i.e. MyChart) or via a letter in the mail.

Patient-Directed Standard of Care

Patients in this arm will be offered genetic services through the patient portal (i.e. MyChart). These educational resources will present standard pre-test genetic counseling information as described in the Enhanced Standard of Care Arm. Patients who decide to proceed with genetic testing will be asked to document consent for testing.

Patients who decide to have genetic testing will have a saliva collection kit, instructions, and shipping materials sent to their homes to collect and return a sample to the testing laboratory; this is a service offered by commercial laboratories. Patients will have an opportunity to contact the genetics clinic with any questions or to request an appointment with a genetic counselor at any time.

All results will be reviewed by a genetic counselor. Negative results will be returned along with a letter including screening recommendations tailored based on family history to the patient and PCP via the patient portal. Genetic counselors will return results for pathogenic variants and variants of uncertain significance via phone. A copy of the results and letter with tailored screening recommendations will be provided to the patient and PCP via the patient portal.

All patients will be offered the option to schedule follow-up appointments in the genetics clinics; these contacts and appointments will be tracked.

Standard of Care Genetic Testing

Genetic testing will be performed at Clinical Laboratory Improvement Act (CLIA)-certified, commercial laboratories. Based on standard clinical practice, the choice of laboratory will be based on services provided and patient's insurance and/or financial status. Testing will be billed to insurance. Patients who are uninsured or lack coverage for genetic testing will be assisted in applying for aid through programs offered by the laboratories.

Genetic testing will not be required to participate in the research questionnaires.

Research Procedures

1. A research coordinator or genetic counseling assistant will determine who is eligible to participate in the research procedures based on participation in genetic services and access to MyChart, the patient portal. A wavier of recruitment is being requested for this authorization. Up to 20,000 unique electronic health records may be accessed to determine inclusion in this study, and basic health outcomes tracked for comparison.

2. Consent cover letter will be sent via patient portal to offer participation in this research study, with a link to an online questionnaire. The consent cover letter will be sent to patients approximately 4 weeks after genetic test results are returned for those who decide to test and about 8 weeks after participation in pre-test genetic counseling services for those who decide not to test.

   If no response is received to the first message, after 1 week another message in the patient portal will be sent. If still no response up to 3 phone calls will be made.

3. Online questionnaires will utilize REDCap, which has a HIPAA-compliant interface. Estimated 960 patients will be offered participation through University of Utah Health; 1,960 patients over both participating sites.

   i. Questionnaire #1: At about **4 weeks after return of results for those who decide to test** (8 weeks after pre-test genetic counseling), we will assess cognitive (i.e., recall, comprehension, uncertainty, risk perceptions), affective (i.e., test-related distress, positive reactions, decision regret), communication (i.e., family, provider) responses, experience with genetic counseling, and sociodemographic characteristics including numeracy for those who decide to test. For those who do not decide to test, we will assess experience with genetic counseling, reason for not testing, decision regret, risk perceptions, and sociodemographic characteristics including numeracy.

   ii. Questionnaire #2: At about **12 months**, we will ask about discussion of their test results with their PCP, family members, or others, and self-reported use of cancer screenings for those who decide to test. We will also utilize EHR data to examine adherence to recommendations. For those who decide not to receive genetic testing, we will assess the self-reported use of cancer screenings, whether genetic testing was pursued at another time or through another source, communication with family members and whether other relatives had testing.

   iii. After each returned questionnaire, participants will be compensated via the patient portal with a thank you message and a $10 e-gift card. This totals $20 dispersed if both questionnaires are returned.

   iv. For any participants who would like to complete questionnaires by telephone, a genetic counseling assistant or research coordinator will administer the questionnaire. Calls will be digitally recorded to allow for analysis of comprehension. Each questionnaire will take about 15 minutes to complete. Participants may also complete the questionnaires by mail if they choose.

4. Study staff will track the EHR of participants who were identified by the CDS algorithms. Any screenings, care or health procedures related to genetic services will be recorded in the research record to determine if an impact is made because of participation in genetic services. This is estimated to be up to 20,000 individual records in the EHR.

5. Data analysis for outcome data will occur after patient enrollment is complete. Patients who return questionnaires will be compared to similar cohorts that were not directly involved with research questionnaires.

   More specific details of data analysis are outlined in the statistical methods section.

7. **Are all procedures for research purposes only (non-standard or non-standard of care procedures)?**

   ◯ Yes ● **No**

   If no, list the procedures that are performed for research purposes only (non-standard or non-standard of care procedures):

   Questionnaire #1: estimated 4-8 weeks from last genetic services contact (4 weeks for those who decide to test and 8 weeks for those who decide not to test)

   Questionnaire #2: estimated 12-months from last genetic services contact

Data Analysis of genetic services impact and outcomes

8. **Is there a safety monitoring plan for this study?**
   ◯ Yes ● **No**

9. **Provide a summary of the statistical methods, data analysis, or data interpretation planned for this study. Factors for determining the proposed sample size (e.g., power) should be stated.**

Research Aims Analysis for this specific IRB:

Aim 2 Analysis.

Aim 2 analyses will address the dimensions of Reach, Effectiveness, Adoption, and Implementation from our conceptual framework. For Effectiveness, we will be testing for equivalence between study arms in uptake of genetic testing. We will check randomization by comparing demographic characteristics between patients in the two arms. Any characteristic(s) that are significantly different will be controlled for in the multivariable analysis. We will examine descriptive statistics, and will use Fisher's Exact test with a two-sided 0.05 significance level for univariate comparison of proportions to examine the association between study arm and uptake of genetic testing. We will next build multilevel multivariable logistic regression models to examine the effect of study arm on the outcome controlling for clinic-level (clinic type, patient volume, patient composition, number of providers), provider-level (specialty, years in practice, gender), and patient-level (e.g., sociodemographic, clinical) variables. We will examine the intra-class correlation (ICC) by clinic and provider. If a significant ICC is observed, the regression models will be extended to include random effects for clinic or provider, and fit using software for generalized mixed models. Covariates will be included in models if significant in unadjusted analyses. For Reach, using the multivariable regression models with interaction terms, we will examine whether the effects of study arm on uptake of genetic testing is modified by race/ethnicity or rurality. For Adoption, we will examine the proportions of providers and patients who access educational resources and with referrals, and whether this differs by population subgroup. For Implementation, we will assess responses to patient portal messages and completion of study workflows. Statistical significance will be assessed as $p<0.05$; SAS 9.4 and/or Stata 15.1 will be used for analysis.

Aim 2 Sample Size

Sample size for Aim 2 was calculated using PASS 15 based on an equivalence test for the difference of two proportions in a cluster-randomized design. Sample sizes of 480 in the patient-directed arm and 480 in enhanced standard of care arm, which were obtained by sampling 32 clusters (clinics) with 15 participants each and 16 clusters (clinics) with 30 participants each, respectively, achieve 80% power to detect equivalence. The margin of equivalence, given in terms of the difference between proportions, extends from -0.10 to 0.10. The calculations assume Likelihood Score Tests (Farrington & Manning) were used with an ICC of 0.05, and a significance level of 0.05. We believe that this is likely an overestimate of ICC based on our prior work. This sample size is sufficient for a multilevel multivariable regression analysis and achieves >95% power to detect a small effect size (0.02) attributable to 1 independent variable using an F-Test with an alpha of 0.05, adjusted for an additional 20 independent variables. To examine effect modification, sample size is calculated based on logistic regression of a binary response variable (Y) on a binary independent variable (X) and a binary confounder variable (Z). A sample size of 1,710 observations achieves 80% power at a 0.05 significance level to detect the X-Z interaction odds ratio of 1.5, using a two-sided Wald test. We will therefore enroll 1,920 patients in the Aim 2 trial to allow for about 10% attrition.

Aim 3 Analysis

Aim 3 analysis will follow analytic procedures to address the dimension of Effectiveness from our conceptual framework. For comprehension, concordance between patient responses and information from genetic counselors' notes will be scored. We will test bivariate relationships between the outcome variables and study arm using Student's t-test or the nonparametric Wilcoxon rank-sum test between continuous variables, and chi-square test (or Fisher's exact test if small cell counts) for categorical data. We will examine distributions for continuous outcomes and transform the data if skewed. Procedures for building multilevel multivariable linear (continuous outcomes) or logistic (binary outcomes) regression models are described above. Regression models extended to include random effects for clinic or provider will be fit using software for linear mixed models for continuous outcomes and generalized linear mixed models for categorical outcomes. As with Aim 2, we will examine effect modification by testing for significant interactions between variables in regression models.

Aim 3 Sample Size

We estimate a sample size of 1,100 for a multilevel multivariable regression analysis with >90% power to detect a small effect size (0.02) attributable to 2 independent variables using an F-Test with an alpha of 0.05 using PASS 15 Software. The variables tested are adjusted for an additional 20 independent variables. To examine effect modification sample size is calculated based on logistic regression of a binary response variable (Y) on a binary independent variable (X) and a binary confounder variable (Z) with a sample size of 1,710 observations achieves 80% power at a 0.05 significance level to detect the X-Z interaction odds ratio of 1.5. Calculations assume that a two-sided Wald test is used.

IRB_00123563 **Created:** 6/5/2019 3:01 PM

PI: Kimberly Kaphingst ScD **Submitted:** 3/5/2019

Title: Broadening the Reach, Impact, and Delivery of Genetic Services

IRB_00123563
- Request for Waiver of Consent

Request for Waiver or Alteration of Consent

* Requested Waivers

	Date Created	Type of Request	Purpose of Waiver Request
View	3/18/2019	Waiver of Informed Consent	Wavier of consent for chart review without patient contact

IRB_00123563 **Created:** 6/5/2019 3:01 PM

PI: Kimberly Kaphingst ScD **Submitted:** 3/5/2019

Title: Broadening the Reach, Impact, and Delivery of Genetic Services

IRB_00123563
IRB Smart Form

Request for Waiver or Alteration of Consent

1. **Purpose of the Waiver Request:**
   Wavier of consent for chart review without patient contact

2. **Type of Request:**
   Waiver of Informed Consent

3. **List the identifying information you plan to collect or keep a link to (e.g. names, dates, or identification numbers such as social security numbers or medical record numbers, etc.).**
   Name and contact information
   Demographics
   Race/ethnicity
   Prior referral to genetic counseling
   Family history
   Genetic counseling and testing
   Primary care provider
   Date of last appointment
   History of reading and responding to messages via the patient portal
   Other health conditions

   Identifiers will be deleted from the research record, but we will keep the other information used in planning patient recruitment in order to compare differences between those who did and did not participate in the research questionnaires.

4. **Explain why the research could not be practicably conducted without using identifiable information. Examples of such explanation could include the following:**
   Identifiable information is needed to determine health outcomes in regards to genetic delivery services, in alignment with the research aims.

5. **Explain why the research could not practicably be conducted without the waiver or alteration. For example, complete the following sentence "If I had to obtain consent, the research could not be conducted because...":**
   The investigators would be unable to answer the research question if consent were necessary for chart review without any patient contact. In addition the time for staff to obtain consent to review the EHR would be inefficient for practicable analysis of how genetic delivery impacts primary care.

6. **Explain why the research and privacy risk of the research are no more than *minimal:***
   Because the review of the patient's electronic heath records is specific in data abstraction and will be hosted on secure university servers. Breach of confidentially is a risk, but minimal and unlikely.

7. **Describe the measures you will take to ensure the waiver or alteration will not adversely affect the rights and welfare of the *subjects:***
   The information to be collected is standard of care and research use of such information will not alter care received.

8. **Explain how you will, if applicable and appropriate, provide the subjects with additional pertinent information *after* they have participated in the study, or indicate *"Not applicable":***

Not applicable

Consent Process

1. **The following investigators and internal staff will obtain consent (as indicated on the Study Location and Sponsors Page):**

Jo Anson	University of Utah
Whitney Espinel	University of Utah
Amanda Gammon	University of Utah
Kinley Garfield	University of Utah
Samantha Greenberg	University of Utah
Kelsi Hagerty	University of Utah
Kadyn Kimball	University of Utah
Wendy Kohlmann	University of Utah
Cathryn Koptiuch	University of Utah
Ryan Mooney	University of Utah
Katie Tobik	University of Utah
Jennie Vagher	University of Utah

List by name, role, and affiliation any others who will obtain consent (e.g. Dr. John Smith, Co-Investigator, etc.).
Jo Anson, Kadyn Kimball-study coordinator
Kelsi Hagerty, Ryan Mooney, Katie Tobik-genetic counseling assistant(s)

Wendy Kohlmann, Co-I, senior genetic counselor

HCI Genetic Counseling Shared Resource (GCSR) Team:
Whitney Espinel
Amanda Gammon
Kinley Garfield
Samantha Greenberg
Cathryn Koptiuch

All are certified, licensed genetic counselors who will conduct all genetic counseling following the standard FCAC clinical practices.

2. **Describe the location(s) where consent will be obtained.**
Consent will be obtained electronically via the web-based patient portal

3. **Describe the consent process(es), including the timing of consent. Describe whether there is a waiting period between the consent process and obtaining consent from the participant (i.e., any time between informing participants and actually obtaining consent).**
Patients who receive genetic services, whether or not they decide to test, will be sent the consent cover letter via patient portal inviting them to participate in the research procedures, with a link to an online questionnaire. The consent cover letter will be sent 4 weeks after genetic test results are returned for those who decide to test and about 8 weeks after participation in pre-test genetic counseling services for those who decide not to test. If no response is received to the

first message, after 1 week another message in the patient portal will be sent. If still no response up to 3 phone calls will be made.

4. **Describe what measures will be taken to minimize the possibility of coercion or undue influence.**
   The consent cover letter will explain to the patient that not participating in this study will have no effect on the clinical care they receive.

5. **Describe the provisions that are made to allow adequate time to exchange information and questions between the investigator and participant.**
   Participants are encouraged to ask questions and are given the contact information for Dr. Kaphingst (one of the Principal Investigators) if any questions or concerns come up after the introduction of the study.

6. **Will a legally authorized representative (LAR) be used?**
   ○ Yes ● **No**

7. **Will a language other than English be used to obtain consent?**
   ○ Yes ● **No**

8. **Are you requesting that documentation of informed consent be waived by the IRB (a consent process in place, but no documentation of consent, e.g. questionnaire cover letter, web-based consent, consent without signature, etc.)?**
   ● **Yes** ○ No

   If yes, complete the following:

   a. **Explain why the waiver of consent documentation is being requested.**
   The research procedures in the study are two follow-up questionnaires. These procedures present no more than minimal risk of harm to subjects and involve no procedures for which consent is normally required outside of the research context.

   b. **Justification for the waiver is one of the following:**
   The research presents no more than minimal risk of harm to subjects and involves no procedures for which written consent is normally required outside of the research context.

5. Data Monitoring Plan

1. **Privacy Protections:** Privacy refers to persons and to their interest in controlling access of others to themselves. Privacy can be defined in terms of having control over the extent, timing and circumstances of sharing oneself (physically, behaviorally, or intellectually) with others. **What precautions will be used to ensure subject privacy is protected?**

Select all that apply:

The research intervention is conducted in a private place

The collection of information about participants is limited to the amount necessary to achieve the aims of the research, so that no unneeded information is being collected

De-identification of photos, audio tapes, or video tapes of the participant that will be made during the study

Other or additional details (specify):

All EHR and questionnaire data collected as part of the study will be computerized, maintained, and secured in a local database at either UU or NYU with password protection (at both the system and file levels). Any hardcopy data gathered will be stored in secure rooms or locked filing cabinets.

Under the direction of Dr. Kaphingst at UU and Dr. Ginsburg at NYU, all data will be safeguarded and identifiable data will be accessible only by individuals involved in the research project on a need-to-know basis. Personally identifying contact information (e.g., name, email addresses, mailing addresses) will be kept in a separate, password-protected file. No names will be used on any data representations; identifying data are only available to and used by the individuals involved in contacting and/ or counseling the study subjects. Data sent from NYU to UU for analysis will be de-identified. Any identifying material that is no longer needed will be shredded.

Consistent with the NCI Cancer Moonshot Public Access and Data Sharing Policy, we will share de-identified, non-genomic individual-level data with a data repository such as the Cancer Epidemiology Data Repository (CEDR). Underlying primary data will be de-identified according to the standards set forth in the HHS Regulations for the Protection of Human Subjects to ensure that the identities of research subjects cannot be readily ascertained with the data and will be stripped of identifiers according to the HIPAA Privacy Rule. The consent document will describe sharing of de-identified individual-level human subjects data with the data repository and researchers at other institutions.

Participants will be told that they do not have to answer any questionnaire items that make them uncomfortable.

2. **Confidentiality Precautions:** Confidentiality is an extension of the concept of privacy; it refers to the subject's understanding of, and agreement to, the ways identifiable information will be stored and shared. Identifiable information can be printed information, electronic information or visual information such as photographs. **What precautions will be used to maintain the confidentiality of identifiable information?**

Select all that apply:

Storing research data on password protected computers or in locked cabinets or offices

Participant identifiers will be stored separately from the coded, participant data

Other or additional details (specify):

All EHR and questionnaire data collected as part of the study will be computerized, maintained, and secured in a local database at either UU or NYU with password protection (at both the system and file levels). Any hardcopy data gathered will be stored in secure rooms or locked filing cabinets.

Under the direction of Dr. Kaphingst at UU and Dr. Ginsburg at NYU, all data will be safeguarded and identifiable data will be accessible only by individuals involved in the research project on a need-to-know basis. Personally identifying contact information (e.g., name, email addresses, mailing addresses) will be kept in a separate, password-protected file. No names will be used on any data representations; identifying data are only available to and used by the individuals involved in contacting and/ or counseling the study subjects. Data sent from NYU to UU for analysis will be de-identified. Any identifying material that is no longer needed will be shredded.

Consistent with the NCI Cancer Moonshot Public Access and Data Sharing Policy, we will share de-identified, non-

genomic individual-level data with a data repository such as the Cancer Epidemiology Data Repository (CEDR). Underlying primary data will be de-identified according to the standards set forth in the HHS Regulations for the Protection of Human Subjects to ensure that the identities of research subjects cannot be readily ascertained with the data and will be stripped of identifiers according to the HIPAA Privacy Rule. The consent document will describe sharing of de-identified individual-level human subjects data with the data repository and researchers at other institutions.

Participants will be told that they do not have to answer any questionnaire items that make them uncomfortable.

3. **Will photos, audio recordings, or video recordings, or medical images of participants be made during the study?**

   ● **Yes** ○ No

   If yes, describe the recording/images and what will become of them after creation (e.g., shown at scientific meetings, stored in the medical/research record, transcribed, erased, etc.):

   For any patients who would like to complete questionnaires by telephone, a genetic counseling assistant or research coordinator will administer the questionnaire. Calls will be digitally recorded to allow for analysis of comprehension. These digital recordings will be stored in the research record and erased at the end of the study.

4. **How will study data and documentation be monitored throughout the study?**

   Select all that apply:

   Periodic review and confirmation of participant eligibility

   Periodic review of the transfer/transcription of data from the original source to the research record

   Other additional details (specify):

5. **Who will be the primary monitor of the study data and documentation?**

   Select all that apply:

   Principal Investigator

   Study Coordinator or Research Nurse

   Other or additional details (specify):

   Other or additional details (specify):

   HCI Population Sciences Regulatory Team, working with Dr. Kaphingst and the Study Coordinator at Utah

6. **How often is study data and documentation monitoring planned (e.g., monthly, twice a year, annually, after N participants are enrolled, etc.)?**

   annually

IRB_00123563 **Created:** 6/5/2019 3:01 PM

PI: Kimberly Kaphingst ScD **Submitted:** 3/5/2019

Title: Broadening the Reach, Impact, and Delivery of Genetic Services

IRB_00123563
6. Risks and Benefits

6. Risks and Benefits

1. **Describe the reasonable foreseeable risks or discomforts to the participants:**

   The risks of this study are minimal. The participant may feel upset thinking about or talking about personal information related to genetic testing. These risks are similar to the experience when discussing personal information with others. Some questions could make participants feel distressed or uncomfortable.

2. **Describe the potential benefits to society AND to participants (do not include compensation):**

   Participants may receive some benefit from this research. They may learn more about any inherited cancer risks. The potential benefit to society is to allow us to understand whether a CDS infrastructure can improve identification of primary care patients who may qualify for genetic risk assessment, and whether a patient-directed standard of care model to cancer genetic services delivery has equivalent outcomes to an enhanced standard of care model.

3. **Are there any costs to the participants from participation in research?**

   ○ Yes ● **No**

   If yes, specify:

4. **Is there any compensation to the participants?**

   ● **Yes** ○ No

   a. **If yes, answer the following:**
      Specify overall amount:
      Participants who return the questionnaires at week 4 and at month 12 will be compensated for each individual questionnaire.

   b. **Specify when participants will be paid (e.g. at each visit, at end of study, etc.):**
      A $10 gift card will be sent to participants once a questionnaire has been received back.

   c. **If applicable, please specify payment by visit or other time interval (e.g. $10 per visit, etc.):**
      $10 per questionnaire, for a maximum compensation of $20.

   d. **If applicable, explain plan for prorating payments if participant does not complete the study:**
      Will not prorate compensation.

7. HIPAA and the Covered Entity

1. **Does this study involve Protected Health Information (PHI) or de-identified health information?**
   ● **Yes** ○ No

   a. **Select the method(s) of authorization that will be used:**

   Waiver or Alteration of Authorization

   Limited data set

   De-identified

   Select the method of de-identification: Safe Harbor De-Identification

   b. **Will PHI be disclosed outside the Covered Entity?**
   ● **Yes** ○ No

   To whom?
   NIH/NCI

   And for what purposes?
   To follow the Cancer Moonshot Public Access and Data Sharing Policy and other regulatory needs.

Does this study involve any of the following:

2. **The investigational use of a drug?**
   ○ Yes ● **No**
   Mark yes, for an expanded access application.

3. **The investigational use of a medical device or humanitarian use device?**
   ○ Yes ● **No**
   Mark yes, for an expanded access application.

4. **The investigational use of a dietary supplement, food, or cosmetic?**
   ○ Yes ○ No

5. **Is this an investigator-initiated drug or device trial lead by the Principal Investigator'**
   ○ Yes ● **No**
   All investigator-initiated drug or device trials are required to have a full research protocol attached to the Documents and Attachments page.

6. **Will this study involve the use of an imaging modality from the department of Radiology?**
   ○ Yes ○ No

7. **Exposure to radioisotopes or ionizing radiation?**
   ○ Yes ● **No**

8. **Genetic testing and/or analysis of genetic data?**
   ○ Yes ● **No**

9. **Creating or sending data and/or samples to a repository to be saved for future research uses?**
   ○ Yes ● **No**

10. **Are you:**
    - **Collecting samples of blood, organs or tissues from participants for research purposes;**
    - **Introducing Recombinant or Synthetic Nucleic Acids (e.g. viral vectors, oligonucleotides) or cells containing recombinant nucleic acids (e.g. CAR-T) into participants; OR**
    - **Introducing other biological materials (e.g. bacteria, viruses) into participants.**

    ○ Yes ● **No**

11. **Does this study involve any of the following?**
    - **Cancer Patients**
    - **Cancer Hypothesis**
    - **Cancer risk reduction**
    - **Cancer prevention**

    ● **Yes** ○ No

12. **Any component of the Clinical and Translational Science Institute (CTSI)?**
    ○ Yes ● **No**

    The Clinical Research Center (CRC)?
    ○ Yes ● **No**

IRB_00123563 **Created:** 6/5/2019 3:01 PM

PI: Kimberly Kaphingst ScD **Submitted:** 3/5/2019

Title: Broadening the Reach, Impact, and Delivery of Genetic Services

IRB_00123563
- Request for Waiver of Authorization

Request for Waiver or Alteration of Authorization

Request for Waiver of Authorization for Recruitment Only

This option must only be used if you are reviewing PHI in order to identify eligible participants BEFORE approaching them to obtain consent and authorization. All other waiver requests must be entered below.

Waiver of Authorization for Recruitment Requested

Other Requests for Waivers of Authorization:

- *Click "Add" below to add a new waiver request to this application.*
- *Click the waiver name link to edit a waiver that has already been created.*
- *To delete a waiver request, contact the IRB.*

	Date Created	Type of Request	Purpose of Waiver Request
View	2/21/2019	Waiver of Authorization	Waver of Authorization for access to EHR for chart review of patients that are identified by CDS tool for inclusion/exclusion criteria. This would be to track patients utilizing genetic services up to 24 months to assess the impact of genetic services on their health and/or health decisions.
View	3/18/2019	Waiver of Authorization	Wavier of authorization to not include authorization language in the consent cover letter.

IRB_00123563 **Created:** 6/5/2019 3:01 PM

PI: Kimberly Kaphingst ScD **Submitted:** 3/5/2019

Title: Broadening the Reach, Impact, and Delivery of Genetic Services

IRB_00123563
Waiver of Authorization for Recruitment Only

Request for Waiver or Alteration of Authorization

Request for Waiver of Authorization for Recruitment Only

The PI must agree to the terms of this waiver request as described on this page. When the PI uses the "Submit" activity to submit the application for IRB review, a checkbox to accept the terms will be available in the "Submit" activity window.

This waiver request includes justification for waivers of consent for recruitment only, according to 45 CFR 46.116(d).

Terms for the Waiver of Authorization:

- The purpose of this waiver of authorization is to allow for the use of PHI in order to identify and recruit individuals who may be eligible to participate in the specific research described in this IRB application. The waiver of authorization is necessary to accommodate this minimal-risk research activity prior to seeking a full authorization from research participants.

- Methods for identifying individuals may include the following:
    - Reviewing medical charts
    - Reviewing databases that include PHI
    - Reviewing other medical- or health-based documents that include PHI

- Identifiable information used under this waiver may include the following, as this is the minimum necessary for identifying eligible individuals:
    - Name
    - Contact information, such as phone number, address, or email address
    - An ID number, such as MRN or SSN
    - Date of birth
    - Medical and health information that may determine study eligibility

- Any PHI recorded by the study team will only be used for recruitment and determining study eligibility. After this has been completed, the PHI must be removed from the research record or destroyed, unless the participants have given authorization for continued use of the PHI.

- PHI will only be viewed by approved members of the study team and will not be disclosed for research purposes to any individual or institution without the participants' authorization for such use and disclosure of the PHI.

- PHI will be stored in a secure manner according to HIPAA privacy and security provisions.

IRB_00123563 **Created:** 6/5/2019 3:01 PM

PI: Kimberly Kaphingst ScD **Submitted:** 3/5/2019

Title: Broadening the Reach, Impact, and Delivery of Genetic Services

Request for Waiver or Alteration of Authorization

1. **Purpose of the Waiver Request:**
Waver of Authorization for access to EHR for chart review of patients that are identified by CDS tool for inclusion/exclusion criteria. This would be to track patients utilizing genetic services up to 24 months to assess the impact of genetic services on their health and/or health decisions.

2. **Type of Request:**
Waiver of Authorization

3. **List the identifying information you plan to collect or keep a link to (e.g. names, dates, or identification numbers such as social security numbers or medical record numbers, etc).**
We would like to collect the following information on patients identified to meet criteria in order to determine eligibility, invite them to participate, and to be able to compare those who participate and those who decline:

   Name and contact information
   Demographics
   Race/ethnicity
   Prior referral to genetic counseling
   Family history
   Genetic counseling and testing
   Primary care provider
   Date of last appointment
   History of reading and responding to messages via the patient portal
   Other health conditions

   Identifiers will be deleted from the research record, but we will keep the other information used in planning patient recruitment in order to compare differences between those who did and did not participate in the research questionnaires.

4. **Explain why the *PHI* to be used or disclosed is the minimum necessary to accomplish the research objectives:**
The PHI data will be used only better define which populations are utilizing genetic services, and if different delivery models impact a patient's health or their primary care decisions.

5. **Explain why the research could not practicably be conducted without the waiver of authorization.** *For example, complete the following sentence: "If I had to obtain authorization, the research could not be conducted because..."*
The research could not be conducted because there is no other non-intrusive or unbiased way to determine patient adherence to recommendations from the genetic services.

   Study team requests authorization to access patient EHR who participate in genetic services. If not, no insights can be gained to determine the efficacy of genetic services in different patient populations, as well as the impact of genetic counseling, testing and education in collaboration with primary care.

6. **Describe your plan to protect the identifiers from improper use and disclosure, and indicate where the *PHI* will be stored and who will have access:**
Study team will work closely with HCI FCAC genetic counselors and genetic counseling assistants to minimize PHI used in the research record. Unique

participant identifiers will be used as name-holders.

Only authorized personnel listed on this IRB application, with updated trainings, will have access to study data and tracking methods.

7. **The identifiers must be destroyed at the earliest opportunity consistent with conduct of the research, unless there is a health or research justification for retaining the identifiers or such retention is otherwise required by law. Describe how and when you will destroy the identifiers, or justify their retention:**
PHI identifiers will be destroyed at the end of this study, when the IRB is fully closed to research activity.

8. **Describe the measures you will take to ensure the PHI will not be reused or disclosed to any other person or entity, except as required by law, for authorized oversight of the research study, or for other research approved by the IRB:**
Only individuals listed on the IRB will access patient EHR, and associated study records kept for research aims.

IRB_00123563 **Created:** 6/5/2019 3:01 PM

PI: Kimberly Kaphingst ScD **Submitted:** 3/5/2019

Title: Broadening the Reach, Impact, and Delivery of Genetic Services

IRB_00123563
IRB Smart Form

Request for Waiver or Alteration of Authorization

1. **Purpose of the Waiver Request:**
Wavier of authorization to not include authorization language in the consent cover letter.

2. **Type of Request:**
Waiver of Authorization

3. **List the identifying information you plan to collect or keep a link to (e.g. names, dates, or identification numbers such as social security numbers or medical record numbers, etc).**
Name and contact information
Demographics
Race/ethnicity
Prior referral to genetic counseling
Family history
Genetic counseling and testing
Primary care provider
Date of last appointment
History of reading and responding to messages via the patient portal
Other health conditions

4. **Explain why the *PHI* to be used or disclosed is the minimum necessary to accomplish the research objectives:**
The PHI data will be used only better define which populations are utilizing genetic services, and if different delivery models impact a patient's health or their primary care decisions.

5. **Explain why the research could not practicably be conducted without the waiver of authorization.** *For example, complete the following sentence: "If I had to obtain authorization, the research could not be conducted because..."*
The research could not be conducted because there is no other non-intrusive or unbiased way to determine patient adherence to recommendations from the genetic services.

   Study team requests authorization to access patient EHR who participate in genetic services. If not, no insights can be gained to determine the efficacy of genetic services in different patient populations, as well as the impact of genetic counseling, testing and education in collaboration with primary care.

6. **Describe your plan to protect the identifiers from improper use and disclosure, and indicate where the *PHI* will be stored and who will have access:**
Study team will work closely with HCI FCAC genetic counselors and genetic counseling assistants to minimize PHI used in the research record. Unique participant identifiers will be used as name-holders.

   Only authorized personnel listed on this IRB application, with updated trainings, will have access to study data and tracking methods.

7. **The identifiers must be destroyed at the earliest opportunity consistent with conduct of the research, unless there is a health or research justification for retaining the identifiers or such retention is otherwise required by law. Describe how and when you will destroy the identifiers, or justify their retention:**

PHI identifiers will be destroyed at the end of this study, when the IRB is fully closed to research activity.

8. **Describe the measures you will take to ensure the PHI will not be reused or disclosed to any other person or entity, except as required by law, for authorized oversight of the research study, or for other research approved by the IRB:**
Only individuals listed on the IRB will access patient EHR, and associated study records kept for research aims.

IRB_00123563 **Created:** 6/5/2019 3:01 PM

PI: Kimberly Kaphingst ScD **Submitted:** 3/5/2019

Title: Broadening the Reach, Impact, and Delivery of Genetic Services

IRB_00123563
- - Information for Accounting of Disclosures

Information for Accounting of Disclosures

1. **Earliest planned date of disclosure:** 7/1/2019
2. **Latest planned date of disclosure:** 3/1/2025
3. **Name and address of the entity or person outside of the Covered Entity who will receive the Protected Health Information:**
   National Cancer Institute
4. **A brief description of the PHI disclosed:**
   Whatever information is requested under the Moonshot Underlying Primary Data Policy. The NCI defines underlying primary data as: "recorded factual material commonly accepted in the scientific community as necessary to document and support research findings in Publications."
5. **A brief statement of the purpose of the disclosure that reasonably informs the individual whose information is disclosed of the basis for the disclosure:**
   To follow the Cancer Moonshot Public Access and Data Sharing Policy and other regulatory needs.

   https://www.cancer.gov/research/key-initiatives/moonshot-cancer-initiative/funding/public-access-policy?redirect=true

IRB_00123563 **Created:** 6/5/2019 3:01 PM

PI: Kimberly Kaphingst ScD **Submitted:** 3/5/2019

Title: Broadening the Reach, Impact, and Delivery of Genetic Services

IRB_00123563
- Limited Data Set Agreement

Limited Data Set Statement and Assurance

This assurance applies to the following part(s) of this study (select all that apply):

- ☐ **All of the information used or disclosed in this study**
- ☑ **The information received or collected from these sources:** New York University
- ☑ **The information shared with or disclosed to these groups:** New York University

Data Use Assurance:

1. As an employee of the University of Utah and the Principal Investigator for the attached study, I understand that I must comply with the requirements below regarding the permitted uses and disclosure of the limited data set I am receiving from the University.
2. I have described the limited data set with specificity in the Protocol submitted with this form.
3. I have described who will be permitted to use or receive the limited data set in the same Protocol.
4. If I am disclosing the limited data set outside the Covered Entity, I am submitting, in addition to this form, a Limited Data Set Statement and Assurance (if the recipient is within the University of Utah) or a Data Use Agreement (if the recipient is outside the University of Utah) signed by an individual able to bind the entity receiving the limited data set.
5. I understand that the use of the limited data set is governed by federal law (45 CFR Parts 160 and 164, particularly 164.514(e)).
6. I agree to the following:
   a. I will only use or disclose the information as described or permitted in the Protocol, or as permitted in writing by the Institutional Review Board;
   b. I will use appropriate safeguards, which I have described in the Protocol, to prevent use or disclosure of the information in any ways outside the Protocol or as permitted in writing by the Institutional Review Board;
   c. I will promptly report to the IRB any use or disclosure of the information not provided for in the Protocol;
   d. I will take all reasonable measures to ensure that any agents, including any subcontractors, to whom I provide the limited data set will follow the same restrictions and conditions regarding that information that I have set forth in the Protocol, and will report any violations to the Institutional Review Board (801 581-3655); and
   e. I will not attempt to identify the information and will not contact the individuals.

Limited Data Set Statement.
I declare that none of the following types of information, regarding subjects or relatives, employers, or household members of subjects, are used in this study:

1. Names;
2. Postal address information (but town or city, State, and zip code may be kept);
3. Telephone numbers;

4. Fax numbers;
5. Electronic mail addresses;
6. Social security numbers;
7. Medical record numbers;
8. Health plan beneficiary numbers;
9. Account numbers;
10. Certificate/license numbers;
11. Vehicle identifiers and serial numbers, including license plate numbers;
12. Device identifiers and serial numbers;
13. Web Universal Resource Locators (URLs);
14. Internet Protocol (IP) address numbers;
15. Biometric identifiers, including finger and voice prints; and
16. Full face photographic images and any comparable images.

IRB_00123563 **Created:** 6/5/2019 3:01 PM

PI: Kimberly Kaphingst ScD **Submitted:** 3/5/2019

Title: Broadening the Reach, Impact, and Delivery of Genetic Services

IRB_00123563 - Safe Harbor De-Identification Agreement

Safe Harbor De-Identification

1. **This declaration applies to the following part(s) of this study:**
   A. ☐ All of the information used or disclosed in this study.
   B. ☐ The information received or collected from these sources:
   C. ☑ The information shared with or disclosed to these groups: CEDR: Cancer Epidemiology Data Repository
2. **As the principal investigator for this study, I declare the following:**

   1. To the best of my knowledge, the information could not be used (alone or with other information) to identify an individual who is a subject of the information, and
   2. None of the following types of information, regarding subjects or relatives, employers, or household members of subjects, are used or disclosed in the part of this study indicated above:
      a. Names;
      b. All geographic identifiers except state or the first three digits of a zip code (however, all data from the following 17 3-digit zips are combined together under "000": 036, 059, 063, 102, 203, 556, 692, 790, 821, 823, 830, 831, 878, 879, 884, 890, and 893)
      c. The month and day (the year can be kept) from all dates directly related to an individual, including birth date, admission date, discharge date, date of death. Ages over 89 are combined in a single category of "Age 90 and older."
      d. Telephone numbers;
      e. Fax numbers;
      f. Electronic mail addresses;
      g. Social security numbers;
      h. Medical record numbers;
      i. Health plan beneficiary numbers;
      j. Account numbers;
      k. Certificate/license numbers;
      l. Vehicle identifiers and serial numbers, including license plate numbers;
      m. Device identifiers and serial numbers;
      n. Web Universal Resource Locators (URLs);
      o. Internet Protocol (IP) address numbers;

p. Biometric identifiers, including finger and voice prints;

q. Full face photographic images and any comparable images; and,

r. Any other unique identifying number, characteristic, or code, except as permitted for re-identification.

3. If I assign a code or other means of record identification to allow de-identified information to be re-identified,

1. The code or other means of record identification is not derived from or related to information about the individual and is not otherwise capable of being translated so as to identify the individual, and
2. I will not use or disclose the code or other means of record identification for any purpose other than re-identification, and I will not disclose the mechanism for re-identification.

4. Before I allow a code to be used to re-identify this information,

1. If the purpose of the re-identification is within the scope of the original protocol, I will obtain approval of an amendment from the IRB and comply with the requirements of HIPAA; or
2. If the purpose of the re-identification is outside the scope of the original protocol, I will submit a full New Study Application, obtain IRB approval, and comply with the requirements of HIPAA.

IRB_00123563 **Created:** 6/5/2019 3:01 PM

PI: Kimberly Kaphingst ScD **Submitted:** 3/5/2019

Title: Broadening the Reach, Impact, and Delivery of Genetic Services

8. Resources and Responsibilities

1. * **State and justify the qualifications of the study staff:**

   Drs. Saundra Buys and Kimberly Kaphingst will serve as Principal Investigators, with Kenneth Boucher and Wendy Kohlmann as Co-Investigators at Utah.

   The Genetic Counseling Shared Resource will provide the genetic counseling and related services at Utah for the study; the Director, Ms. Kohlmann, of the GCSR is a PI.

   The Cancer Biostatistics Shared Resource will conduct the quantitative analyses at Utah.

   Study and research coordinators with appropriate CITI training will be involved as well.

2. * **Describe the training that study staff and investigators will receive in order to be informed about the protocol and understand their research-related duties and functions:**

   All investigators and study staff will complete required human subjects training. They will meet regularly with Dr. Kaphingst (PI) to discuss study progress. The Population Sciences Trials Office at HCI will train study staff on regulatory requirements and conduct of the research study.

3. * **Describe the facilities where the research activities will be performed (e.g. hospitals, clinics, laboratories, classrooms/schools, offices, tissue banks, etc.).**

   Secure offices at Huntsman Cancer Institute, secure offices at New York University, locations where patients opt to access their patient portal

4. * **Describe the medical or psychological resources available at this site (and other participating sites, if applicable) that participants might require as a consequence of the research. If not applicable, please state.**

   Genetic counselors will be available to answer any participant questions.

5. * **How will adverse events, unanticipated problems, interim results, and changes to the research be communicated between the participating sites and the Principal Investigator?**

   Regular bi-weekly study meetings between main site (Utah) and the participating site (NYU).

IRB_00123563 **Created:** 6/5/2019 3:01 PM

PI: Kimberly Kaphingst ScD **Submitted:** 3/5/2019

Title: Broadening the Reach, Impact, and Delivery of Genetic Services

Documents and Attachments

If any of your documents (such as investigational brochures, sponsor protocols, advertisements, etc.) are not available in an electronic format, please scan and save them as PDF files or contact our office for assistance.

Naming Documents: Please use the title field to clearly indicate the content of each form. The name you enter will be listed on your approval letter. Use names that will differentiate from earlier versions.

Examples:
Consent Document Control Group 04/14/05
Consent Document Treatment Group 4/14/05
Sponsor Protocol 04/14/05 Version 2
Assent Document(Highlighted Changes)

Apple/Macintosh Users:MS Word documents must have a .doc file extension. See ERICA home page for instructions.

Print View: IRB Draft Protocol Summary

eProtocol Summary:

Name	Version	Date Created	Date Modified	Date Approved

There are no items to display

Consent Documents, Consent Cover Letters, Consent Information Sheets, Consent Scripts, etc.:

Name	Version	Date Created	Date Modified	Date Approved

There are no items to display

Parental Permission Documents:

Name	Version	Date Created	Date Modified	Date Approved

There are no items to display

Assent Documents:

Name	Version	Date Created	Date Modified	Date Approved

There are no items to display

VA Consent Documents:

Name	Version	Date Created	Date Modified	Date Approved

There are no items to display

Surveys, Questionnaires, Interview Scripts, etc.:

Name	Version	Date Created	Date Modified	Date Approved

There are no items to display

Full Protocol (company protocol, sponsor protocol, investigator-initiated protocol, etc.):

Name	Version	Date Created	Date Modified	Date Approved

There are no items to display

Investigational Brochure (IB) for Investigational Drug or Drug/Device Package Insert:

Name	Version	Date Created	Date Modified	Date Approved

There are no items to display

Grant Application:

The Federal Government is a direct or indirect sponsor of your research. You are required to provide a copy of the grant proposal, grant award, or sub-award.

By submitting to the IRB, you are confirming the grant and the study protocol are consistent (Design, Study Population, Study Objectives and Goals, Test Interventions and Procedures, etc.)

Name	Version	Date Created	Date Modified	Date Approved

There are no items to display

Literature Cited/References:

Name	Version	Date Created	Date Modified	Date Approved

There are no items to display

Principal Investigator's Scholarly Record (CV/Resume):

Name	Version	Date Created	Date Modified	Date Approved
Kaphingst CV(0.04)	0.04	4/2/2015 12:18 PM	8/27/2018 3:25 PM	

Faculty Sponsor's Scholarly Record (CV/Resume):

Name	Version	Date Created	Date Modified	Date Approved

There are no items to display

Other Stamped Documents:

Name	Version	Date Created	Date Modified	Date Approved

There are no items to display

Recruitment Materials, Advertisements, etc.:

Name	Version	Date Created	Date Modified	Date Approved

There are no items to display

Other Documents:

Name	Version	Date Created	Date Modified	Date Approved

There are no items to display

IRB_00123563 **Created:** 6/5/2019 3:01 PM

PI: Kimberly Kaphingst ScD **Submitted:** 3/5/2019

Title: Broadening the Reach, Impact, and Delivery of Genetic Services

IRB_00123563
- HCI PRMC

HCI PRMC

UNIVERSITY OF UTAH HUNTSMAN CANCER INSTITUTE

Huntsman Cancer Institute Protocol Review and Monitoring Committee (PRMC) New Project Cover Sheet

HCI Clinical Trials Office
PRMC@hci.utah.edu
(801) 585-6746

Date: 6/5/2019 3:01 PM
Principal Investigator: Kimberly Kaphingst
Department: COMMUNICATION

Site Staff and Enrollment:

Site Name	Investigator	Staff	Enrollment Number
NYU School of Medicine			Not Answered
University of Utah	Kimberly Kaphingst	Priyanka Kanth<br>Sarah Colonna<br>Ryan Mooney<br>Whitney Espinel<br>Saundra Buys<br>Jennie Vagher<br>Katie Tobik<br>Samantha Greenberg<br>Kenneth Boucher<br>Wendy Kohlmann<br>Kadyn Kimball<br>Amanda Gammon<br>David Wetter<br>Kinley Garfield<br>Kensaku Kawamoto<br>Cathryn Koptiuch<br>Anne Naumer<br>Jo Anson<br>Kelsi Hagerty<br>Joshua Schiffman	Not Answered

Total number of subjects enrolled at the University of Utah in all years prior to October 2019: 960 consent cover letters sent; up to 20,000 EHR records

Title: Broadening the Reach, Impact, and Delivery of Genetic Services

The PRMC is charged with the review of the scientific merit, priorities, and progress of cancer research at the University of Utah.

What type of Clinical Research is this?
Health services research: Protocol designed to evaluate the delivery, processes, management, organization, or financing of health care.

Multicenter Study:
Yes

Coordinating Center or Organization:
Huntsman Cancer Institute

Project Funding:

	Sponsor	Sponsor Type	Sponsor Contact Information	Prime Sponsor	Prime Sponsor Type	OrgID
View	NIH NATIONAL CANCER INSTITUTE	Federal Government	NCI Grants Management Specialist: Kimery Griffin Email: griffink3@mail.nih.gov Phone: 240-276-6315			11259

Has the Funding Source granted approval to conduct this study:
Approved

1. **Clinical Research Category (Select One):**
   Observational (OBS)

2. **Primary Purpose of the Research (Select One):**
   Health Services Research (HSR)

3. **Study Source (Select One):**
   Externally Peer-Reviewed: R01s, SPORES, U01s, U10s, P01s, CTEP, or any other clinical research study mechanism supported by the NIH or affiliate organizations (click HELP button for a full list)

4. **Study Model Code (Select One):**

5. **Time Perspective Code (Select One):**

6. **Have project statistics been reviewed by a statistician:**

7. **How will accrual and demographic information be tracked:**
   REDCap

8. **Does this study target a Rare Disease (meaning it targets a cancer with an incident rate of = < 3 per 100,000 per year):**
   ◯ Yes ● **No**

9. **Number of subjects to be enrolled at the University of Utah in year 1:**
   380

10. **Estimated number of potentially eligible subjects seen at the University in the past year:**
    500

11. **Source of data (e.g. CTRG estimate, cancer registry, clinic stats, TriNetX etc.):**
    FCAC clinic stats

12. **Estimated time (in months) for full accrual at the University of Utah:**

2.5 years

13. **Is this study run through the HCI Clinical Trials Office:**

    No

14. **Department overseeing this research:**

Comments:

IRB_00123563 **Created:** 6/5/2019 3:01 PM

PI: Kimberly Kaphingst ScD **Submitted:** 3/5/2019

Title: Broadening the Reach, Impact, and Delivery of Genetic Services

IRB_00123563
IRB Smart Form - Read Only

Sponsor Information

a. **Sponsor:**
NIH NATIONAL CANCER INSTITUTE

b. **Sponsor Contact Information:**
Address, phone number, fax number used for event reporting and study correspondence.

NCI Grants Management Specialist: Kimery Griffin
Email: griffink3@mail.nih.gov
Phone: 240-276-6315

c. **If the funding type is "Federal Agency, or federal flow through", provide the following information:**

Grant Number:
1U01CA232826

Grant Awardee (Institution and Investigator):
University of Utah (lead) and NYU

Principal Investigators: Saundra Buys and Kimberly Kaphingst (Utah); Ophira Ginsburg (NYU)

Effective Start Date: 9/1/2018

Effective End Date: 8/31/2023

If the grant has been awarded to the University of Utah, please attach a copy of the grant application to the Documents and Attachments page.

d. **Are you working on this study with the University of Utah Office of Sponsored Projects to obtain this funding?**
Yes

If no, please explain:

A contract with the Office of Sponsored Projects may be required even if you are not receiving direct funds. Any relationship with an outside entity should be discussed with OSP:

Main Campus OSP: ***801.581.6903***
UUHSC OSP: ***801.581.8949***
VAMC OSP: ***801.582.1565 x4866***

IRB_00123563 **Created:** 6/5/2019 3:01 PM **IRB_00123563** Finish

PI: Kimberly Kaphingst ScD **Submitted:** 3/5/2019

Title: Broadening the Reach, Impact, and Delivery of Genetic Services

Finish Instructions

Finish Instructions

1. **To view errors, select the "Validate" option at the top-left of the page. If you have errors on your application, you won't be able to submit it to the IRB.**

2. **Selecting the Finish button will NOT submit the application to the IRB.**
   You MUST select the "Submit" option on the workspace once you've selected the "Finish" button.

3. **If your study has a faculty sponsor: Once the PI submits the application, it will be sent to the faculty sponsor for final approval. The IRB cannot review the study until the faculty sponsor submits the application to the IRB.**

Electronic
RESEARCH INTEGRITY AND COMPLIANCE
THE UNIVERSITY OF UTAH

Date: Monday, May 4, 2020 9:30:11 PM

Print Close

IRB_00115509 - CR_3/9/2020 2:18 PM **Created:** 3/9/2020 2:18 PM

PI: Kimberly Kaphingst **Submitted:** 3/23/2020

Title: Broadening the Reach, Impact, and Delivery of Genetic Services

HELP?

1. Status

Overall, what is the enrollment status for this study?
Open to enrollment of new participants

-

IRB_00115509 - CR_3/9/2020 2:18 PM **Created:** 3/9/2020 2:18 PM

PI: Kimberly Kaphingst **Submitted:** 3/23/2020

Title: Broadening the Reach, Impact, and Delivery of Genetic Services

HELP?

2. Progress of Study and Enrollment Information

Overall Study Enrollment Progress

**Number of currently approved enrollments for this study as a whole:**	1,920 consent cover letters sent; up to 20,000 EHR records

Number enrolled across all sites as of today�s date:0

- **Describe the progress of the study, observations of the participants studied, and reason(s) for continuing the study:**
  Progress for this study remains ongoing although delayed by the technical implementation timeline. Enrollment at the Utah site is expected to commence in April with NYU enrollment projected to begin in June.

  The study team has completed building questionnaires in RedCap and is prepared to begin enrollment once patients have completed the necessary clinical procedures.

- **What results (preliminary or final) have been obtained from the study? If the study is part of a multi-center trial, this should be stated and any available results provided. If there are no results that are appropriate to report to the IRB at this time, this should be stated and explained:**
  Because this study has not enrolled any participants, there are no results to disclose at this point.

Please attach any abstracts or publications:

Name	Version	Date Created	Date Modified	Date Approved

There are no items to display

- **Describe participant enrollment and its effect on the study design and scientific merit. If under-accrued, describe any plans for increasing enrollment during the next approval period. If a multicenter trial, also discuss enrollment at other sites.**
  This study has no participants enrolled. Enrollment is expected to begin at Utah in April and at NYU in June.

IRB_00115509 - CR_3/9/2020 2:18 PM **Created:** 3/9/2020 2:18 PM
PI: Kimberly Kaphingst **Submitted:** 3/23/2020
Title: Broadening the Reach, Impact, and Delivery of Genetic Services

HELP?

Progress of Sites and Enrollment

Select each site to update the enrollment information.

List of Study Controlled Locations

	Site Name	Investigator's Name	Enrollments since Last CR	Enrollments as of Today	Non English Speakers	Withdrawn
View	NYU School of Medicine	Ophira Ginsburg		0	0	0
View	University of Utah	Kimberly Kaphingst		0	0	0

IRB_00115509 - CR_3/9/2020 2:18 PM **Created:** 3/9/2020 2:18 PM
PI: Kimberly Kaphingst **Submitted:** 3/23/2020
Title: Broadening the Reach, Impact, and Delivery of Genetic Services

HELP?

3. Problems & Events

- 1. **Provide narrative summary of any available information regarding adverse events which have occurred since the last IRB review.**
  There have been no adverse events.
- 2. **Have any additional risks to participation in this study been identified (from any site or other relevant source) since last approval? If reports or amendments identifying additional risks have been submitted since last approval, please mark �yes�.**
  ○ Yes ◉ **No**

IRB_00115509 - CR_3/9/2020 2:18 PM **Created:** 3/9/2020 2:18 PM
PI: Kimberly Kaphingst **Submitted:** 3/23/2020
Title: Broadening the Reach, Impact, and Delivery of Genetic Services

HELP?

5. Amendments

A list of amendments associated with this study since the last approval date.

Name	Date Submitted	IRB #
AM_Questionnaires and Pilot	10/7/2019	AM_00035690
AM_Option to email participants and correct questionnaire typo	1/22/2020	AM_00036697

1. **Are there any changes to be made now? (Including scientific design, procedures, participant population, or any minor changes.)**
   ○ Yes ◉ **No**

IRB_00115509 - CR_3/9/2020 2:18 PM **Created:** 3/9/2020 2:18 PM
PI: Kimberly Kaphingst **Submitted:** 3/23/2020
Title: Broadening the Reach, Impact, and Delivery of Genetic Services

HELP?

6. Report Forms

Name	Date Submitted	IRB #

IRB_00115509 - CR_3/9/2020 2:18 PM **Created:** 3/9/2020 2:18 PM
PI: Kimberly Kaphingst **Submitted:** 3/23/2020
Title: Broadening the Reach, Impact, and Delivery of Genetic Services

HELP?

7. Documents and Attachments

Approved eProtocol Summary:

Name	Version	Date Created	Date Modified	Date Approved
ID00000007(0.01)	0.01	5/4/2020 11:28 AM	5/4/2020 11:28 AM	ID00000007

Print View: IRB Draft Protocol Summary

Updated eProtocol Summary:

Name	Version	Date Created	Date Modified	Date Approved
There are no items to display				

Approved Consent Forms:

Name	Version	Date Created	Date Modified	Date Approved
03_2020 BRIDGE consent cover letter V1.2(0.01)	0.01	3/12/2020 3:24 PM	3/12/2020 3:24 PM	5/4/2020 11:28 AM

Updated Consent Forms:

Name	Version	Date Created	Date Modified	Date Approved
There are no items to display				

Approved Parental Permission Forms:

Name	Version	Date Created	Date Modified	Date Approved
There are no items to display				

Updated Parental Permission Forms:

Name	Version	Date Created	Date Modified	Date Approved
There are no items to display				

Approved Assent Forms:

Name	Version	Date Created	Date Modified	Date Approved
There are no items to display				

Updated Assent Forms:

Name	Version	Date Created	Date Modified	Date Approved
There are no items to display				

Approved VA Consent Forms:

Name	Version	Date Created	Date Modified	Date Approved
There are no items to display				

Updated VA Consent Forms:

Name	Version	Date Created	Date Modified	Date Approved
There are no items to display				

Approved Surveys, etc.:

Name	Version	Date Created	Date Modified	Date Approved
Questionnaire #1 - Genetic Testing 10042019 Clean.docx(0.01)	0.01	10/4/2019 3:36 PM	10/4/2019 3:36 PM	11/4/2019 4:30 PM
Questionnaire #1 - No Genetic Testing 100319.docx(0.01)	0.01	10/4/2019 3:36 PM	10/4/2019 3:36 PM	11/4/2019 4:30 PM

Name	Version	Date Created	Date Modified	Date Approved
Questionnaire #2 - No Genetic Testing 10042019 Clean.docx(0.01)	0.01	10/4/2019 3:37 PM	10/4/2019 3:37 PM	11/4/2019 4:30 PM
Questionnaire #2 - Genetic Testing Clean 011320(0.01)	0.01	1/13/2020 10:47 AM	1/13/2020 10:47 AM	1/22/2020 9:28 AM

Updated Surveys, etc.:

Name Version Date Created Date Modified Date Approved

There are no items to display

Approved Company Protocol:

Name Version Date Created Date Modified Date Approved

There are no items to display

Updated Company Protocol:

Name Version Date Created Date Modified Date Approved

There are no items to display

Approved Investigational Brochure:

Name Version Date Created Date Modified Date Approved

There are no items to display

Updated Investigational Brochure:

Name Version Date Created Date Modified Date Approved

There are no items to display

Approved Grant Application:

Name	Version	Date Created	Date Modified	Date Approved
Grant application(0.01)	0.01	1/20/2019 3:03 PM	1/20/2019 3:03 PM	6/5/2019 2:56 PM

Updated Grant Application:

Name Version Date Created Date Modified Date Approved

There are no items to display

Approved Literature/Cited References:

Name	Version	Date Created	Date Modified	Date Approved
Background and Introduction References(0.01)	0.01	2/22/2019 8:32 AM	2/22/2019 8:32 AM	6/5/2019 2:56 PM

Updated Literature/Cited References:

Name Version Date Created Date Modified Date Approved

There are no items to display

Current PI Scholarly Record (CV/Resume):

Name	Version	Date Created	Date Modified	Date Approved
Kaphingst CV(0.04)	0.04	4/2/2015 12:18 PM	8/27/2018 3:25 PM	

Updated PI Scholarly Record (CV/Resume):

Name	Version	Date Created	Date Modified	Date Approved
Kaphingst CV(0.04)	0.04	4/2/2015 12:18 PM	8/27/2018 3:25 PM	

Current Faculty Sponsor Scholarly Record (CV/Resume):

Name Version Date Created Date Modified Date Approved

There are no items to display

Updated Faculty Sponsor Scholarly Record (CV/Resume):

Name Version Date Created Date Modified Date Approved

There are no items to display

Approved Other Stamped Documents:

Name Version Date Created Date Modified Date Approved

There are no items to display

Updated Other Stamped Documents:

Name Version Date Created Date Modified Date Approved

There are no items to display

Approved Recruitment Materials, Advertisements, etc.:

Name	Version	Date Created	Date Modified	Date Approved
Q1 invitation message.docx(0.01)	0.01	10/21/2019 12:20 PM	10/21/2019 12:20 PM	11/4/2019 4:30 PM
Q2 reminder message.docx(0.01)	0.01	10/21/2019 12:20 PM	10/21/2019 12:20 PM	11/4/2019 4:30 PM

Updated Recruitment Materials, Advertisements, etc.:

Name Version Date Created Date Modified Date Approved

There are no items to display

Approved Other Documents:

Name Version Date Created Date Modified Date Approved

There are no items to display

Updated Other Documents:

Name Version Date Created Date Modified Date Approved

There are no items to display

IRB_00115509 - CR_3/9/2020 2:18 PM **Created:** 3/9/2020 2:18 PM

PI: Kimberly Kaphingst **Submitted:** 3/23/2020

Title: Broadening the Reach, Impact, and Delivery of Genetic Services

HELP?

8. Instructions and Finish

1. To view errors in this application, select the "Hide/Show Errors" option at the top or bottom of the page. If you have errors on your application, you won't be able to submit it to the IRB.

Changes to the Update Study Application

2. Be sure to make all proposed changes to the Update Study portion of the application by selecting the "Update Study" button located on the left side of the amendment or continuing review workspace, which will be available once you select the "Finish" button at the top or bottom of this page.
3. To attach updated or new documents with this application, you may access the Documents and Attachments page in the Update Study application.
4. If you are proposing changes to any ancillary applications (i.e. RDRC-HUS or RGE), you must access these applications through the Update Study application on the Ancillary Applications page. All changes to ancillary applications must be approved by the corresponding committee prior to IRB approval of the amendment.

Submitting the Completed Continuing Review Application

5. Selecting the �Finish� button alone will NOT submit the application to the IRB. You MUST also select the "Submit" option on the workspace after you've selected the "Finish" button. Only the PI can submit the application to the IRB.
6. If your study has a faculty sponsor: Once the PI submits the application, it will be sent to the faculty sponsor for final approval. The IRB cannot review the study until the faculty sponsor submits the application to the IRB.

IRB_00115509 - CR_3/9/2020 2:18 PM **Created:** 3/9/2020 2:18 PM

PI: Kimberly Kaphingst **Submitted:** 3/23/2020

Title: Broadening the Reach, Impact, and Delivery of Genetic Services

Study Location Questions

NYU School of Medicine

Site Status

What is the enrollment status for this site?
Open to enrollment of new participants

1,920 consent cover letters sent; up to 20,000 EHR records **Number of currently approved enrollments for this study as a whole**

Enrollment since the beginning of the study at this site:

0**Total number enrolled as of today�s date at this site**

Enrollment since the initial approval at this site:

How many non-English speakers have been enrolled?
0

How many participants signed consent forms but did not end up participating in the study?
0

Problems and Events

Have any unanticipated problems or adverse events involving risks to participants occured since the last IRB approval?
○ Yes ◉ **No**

Have there been any complaints about the research since the last IRB review?
○ Yes ◉ **No**

Local HRPP Reporting

Have any significant financial conflicts of interest for study personnel been identified since the last approval that have not been reported to the IRB?
○ Yes ◉ **No**

Have there been any changes to human research protection reviews or approvals from your institution since last approval that have not been reported to the IRB.
○ Yes ◉ **No**

IRB_00115509 - CR_3/9/2020 2:18 PM **Created:** 3/9/2020 2:18 PM
PI: Kimberly Kaphingst **Submitted:** 3/23/2020
Title: Broadening the Reach, Impact, and Delivery of Genetic Services

Study Location Questions

University of Utah

Site Status

What is the enrollment status for this site?
Open to enrollment of new participants

1,920 consent cover letters sent; up to 20,000 EHR records **Number of currently approved enrollments for this study as a whole**

Enrollment since the beginning of the study at this site:

0**Total number enrolled as of today�s date at this site**

Enrollment since the initial approval at this site:

How many non-English speakers have been enrolled?
0

How many participants signed consent forms but did not end up participating in the study?
0

Problems and Events

Have any unanticipated problems or adverse events involving risks to participants occured since the last IRB approval?
○ Yes ◉ **No**

Have there been any complaints about the research since the last IRB review?
○ Yes ◉ **No**

Electronic RESEARCH INTEGRITY AND COMPLIANCE THE UNIVERSITY OF UTAH

Date: Monday, March 29, 2021 9:31:05 PM

Print Close

IRB_00115509 - CR_2/22/2021 12:04 PM **Created:** 2/22/2021 12:04 PM

PI: Kimberly Kaphingst **Submitted:** 3/16/2021

Title: Broadening the Reach, Impact, and Delivery of Genetic Services

HELP?

1. Status

Overall, what is the enrollment status for this study?
Open to enrollment of new participants

-

IRB_00115509 - CR_2/22/2021 12:04 PM **Created:** 2/22/2021 12:04 PM

PI: Kimberly Kaphingst **Submitted:** 3/16/2021

Title: Broadening the Reach, Impact, and Delivery of Genetic Services

HELP?

2. Progress of Study and Enrollment Information

Overall Study Enrollment Progress

Number of currently approved enrollments for this study as a whole: 1,920 consent cover letters sent; up to 20,000 EHR records

Number enrolled across all sites at last Continuing Review:0

Number enrolled across all sites as of today�s date:36

Number enrolled across all sites since last Continuing Review:36

- **Describe the progress of the study, observations of the participants studied, and reason(s) for continuing the study:**
  Utah began enrollment for their pilot study in April 2020 and enrollment for their full study in November 2020. To date, 81 participants have been invited to participate in Questionnaire #1 and 1 participant has been invited to participate in Questionnaire #2 at this site. Although delayed due to COVID-19, recruitment remains ongoing and enrollment is expected to pick up during 2021 now that the technical implementation has been completed.

  NYU began enrollment for their pilot study in Dec 2020 and enrollment for the full study in February

2020. To date, 23 participants have been invited to participate in Questionnaire #1. The technical implementation was substantially delayed at this site was delayed due to COVID-19 but the study team is confident that they can still meet enrollment goals in the outlined timeframe.

- **What results (preliminary or final) have been obtained from the study? If the study is part of a multi-center trial, this should be stated and any available results provided. If there are no results that are appropriate to report to the IRB at this time, this should be stated and explained:**
  There are no results to disclose at this point.

  Please attach any abstracts or publications:

Name	Version	Date Created	Date Modified	Date Approved

  There are no items to display

- **Describe participant enrollment and its effect on the study design and scientific merit. If under-accrued, describe any plans for increasing enrollment during the next approval period. If a multicenter trial, also discuss enrollment at other sites.**
  Although significantly delayed due to COVID-19 and technical implementation hurdles, enrollment at both sites has officially begun. Now that the technical infrastructure has been completed, tested, and implemented study teams at both sites anticipate the enrollment rate to increase substantially over the next approval period.

IRB_00115509 - CR_2/22/2021 12:04 PM **Created:** 2/22/2021 12:04 PM

PI: Kimberly Kaphingst **Submitted:** 3/16/2021

Title: Broadening the Reach, Impact, and Delivery of Genetic Services

HELP?

Progress of Sites and Enrollment

Select each site to update the enrollment information.

List of Study Controlled Locations

	Site Name	Investigator's Name	Enrollments since Last CR	Enrollments as of Today	Non English Speakers	Withdrawn
View	NYU School of Medicine	Ophira Ginsburg	2	2	0	0
View	University of Utah	Kimberly Kaphingst	34	34	0	0

IRB_00115509 - CR_2/22/2021 12:04 PM **Created:** 2/22/2021 12:04 PM

PI: Kimberly Kaphingst **Submitted:** 3/16/2021

Title: Broadening the Reach, Impact, and Delivery of Genetic Services

HELP?

3. Problems & Events

1. **Provide narrative summary of any available information regarding adverse events which have occurred since the last IRB review.**
- There have been no adverse events.
2. **Have any additional risks to participation in this study been identified (from any site or other relevant source) since last approval? If reports or amendments identifying additional risks have been submitted since last approval, please mark �yes�.**
- ○ Yes ◉ **No**

IRB_00115509 - CR_2/22/2021 12:04 PM **Created:** 2/22/2021 12:04 PM
PI: Kimberly Kaphingst **Submitted:** 3/16/2021
Title: Broadening the Reach, Impact, and Delivery of Genetic Services

HELP?

5. Amendments

A list of amendments associated with this study since the last approval date.

Name	Date Submitted	IRB #
AM_Adding COVID items to questionnaires	5/18/2020	AM_00038160
AM_Questionnaire outreach and study personnel changes	7/15/2020	AM_00038525
AM_Spanish Translations	9/2/2020	AM_00039151
AM_Questionnaire outreach	10/19/2020	AM_00039559

1. **Are there any changes to be made now? (Including scientific design, procedures, participant population, or any minor changes.)**
   ◉ **Yes** ○ No

IRB_00115509 - CR_2/22/2021 12:04 PM **Created:** 2/22/2021 12:04 PM
PI: Kimberly Kaphingst **Submitted:** 3/16/2021
Title: Broadening the Reach, Impact, and Delivery of Genetic Services

HELP?

Amendment Description

1. **Type of Amendment (check all that apply):**
   Administrative changes, for example:
   Changes to study procedures, for example:

Other changes

2. **What changes are being made? List and number each change, grouping similar changes together.**

    1. Administrative Changes
        1. Remove Kelsey Kehoe and Andrew Intveld
        2. Move personnel from guest to study staff
            1. Joshua Schiffman
            2. Sarah Colonna
            3. Whitney Espinel
            4. David Wetter
        3. Add Rick Bradshaw as study staff
    2. Changes to study procedures
        a. Study staff will track COVID-related appointments and diagnoses for patients who were identified by the algorithm.
    3. Other changes
        1. Update of study category in PRMC application.

3. **Describe the reason for each of the changes described above. List and number the reasons according to the list above.**

    1. Administrative Changes
        1. Kelsey Kehoe is no longer at HCI
        2. Guests have had an influence on study design and will be included as authors on publications moving forward
        3. Rick Bradshaw should be added to be up to date with study staff
    2. Changes to study procedures
        a. COVID-related experiences may impact the way patients use and intact with the health system, including genetic services. We would like to report if patients' experience with COVID may have impacted their decision to receive counseling or testing.
    3. Other changes
        1. The study category in the PRMC application will be updated as requested by the CCSG/HCI internal reporting team. We have not made any changes to the study design.

4. **How does each change described above effect participants? List and number the effects according to the list above.**

    1. Administrative changes will have no effect on participants
    2. Changes to study procedures
        a. Tracking COVID-related appointments and diagnoses would not directly affect participants.
    3. Other changes
        1. Changing study category will not affect participants.

5. **Will the modification(s), in the opinion of the local PI, increase or decrease the risk to participants?**
Neither

    If the risk changes, provide justification:

6. **How will enrolled participants (current and past) be notified of this change?**
Participants will not be notified

If Other, please explain:

7. **Which approved documents are affected by these changes?**
   There are no items to display

   If Other, please list

8. **Which sections of the Update Study Application are affected by these changes?**
   1. Contacts and title; #2

   2. Study Location and Sponsors; #1 - University of Utah

   4. Study information; #6

   Request for waiver of consent; Waiver of Informed consent for chart review without patient contact

   Request for waiver of authorization; Waiver of authorization for access to EHR for chart review

   PRMC application; #5

   Request for waiver of authorization; Waiver of authorization to not include authorization language

9. **Select all study locations that are affected by these changes.**

Prior Approved Study Controlled Locations

Site Name	Investigator Name	Covered Entity
☐ NYU School of Medicine	Ophira Ginsburg	Study procedures will be conducted outside a HIPAA Covered Entity at this site (HIPAA Privacy Rule does not apply)
☐ University of Utah	Kaphingst	Study procedures will be conducted within a HIPAA Covered Entity at this site (HIPAA Privacy Rule applies)

New Sites

IRB_00115509 - CR_2/22/2021 12:04 PM **Created:** 2/22/2021 12:04 PM

PI: Kimberly Kaphingst **Submitted:** 3/16/2021

Title: Broadening the Reach, Impact, and Delivery of Genetic Services

HELP?

Amendment Approvals

1. **Is this amendment related to information that meets the IRB reporting policy for unanticipated problems and non-compliance?**
   ○ Yes ◉ **No**

   If yes, a Report Form must also be submitted and then linked to this amendment. Link the related report form to the amendment application by selecting the report form from the list below.

ID	Name	Date Submitted	Status

2. **Investigational Drug Data Form (IDDF) Changes:** ○ Yes ◉ No

3. **Are you adding the VA as a site?** ○ Yes ◉ No

IRB_00115509 - CR_2/22/2021 12:04 PM **Created:** 2/22/2021 12:04 PM
PI: Kimberly Kaphingst **Submitted:** 3/16/2021
Title: Broadening the Reach, Impact, and Delivery of Genetic Services

HELP?

6. Report Forms

Name	Date Submitted	IRB #

IRB_00115509 - CR_2/22/2021 12:04 PM **Created:** 2/22/2021 12:04 PM
PI: Kimberly Kaphingst **Submitted:** 3/16/2021
Title: Broadening the Reach, Impact, and Delivery of Genetic Services

HELP?

7. Documents and Attachments

Approved eProtocol Summary:

Name	Version	Date Created	Date Modified	Date Approved
ID00000007(0.01)	0.01	3/29/2021 11:11 AM	3/29/2021 11:11 AM	ID00000007

Print View: IRB Draft Protocol Summary

Updated eProtocol Summary:

Name	Version	Date Created	Date Modified	Date Approved

There are no items to display

Approved Consent Forms:

Name	Version	Date Created	Date Modified	Date Approved
Consent Cover Letter V1.2_CR2021.docx(0.01)	0.01	3/10/2021 12:55 PM	3/10/2021 12:55 PM	3/29/2021 11:11 AM
Consent Cover Letter V1.2_CR2021_Spanish.docx(0.01)	0.01	3/10/2021 12:55 PM	3/10/2021 12:55 PM	3/29/2021 11:11 AM

Updated Consent Forms:

Name	Version	Date Created	Date Modified	Date Approved

There are no items to display

Approved Parental Permission Forms:

Name	Version	Date Created	Date Modified	Date Approved

There are no items to display

Updated Parental Permission Forms:

Name	Version	Date Created	Date Modified	Date Approved

There are no items to display

Approved Assent Forms:

Name	Version	Date Created	Date Modified	Date Approved

There are no items to display

Updated Assent Forms:

Name	Version	Date Created	Date Modified	Date Approved

There are no items to display

Approved VA Consent Forms:

Name	Version	Date Created	Date Modified	Date Approved

There are no items to display

Updated VA Consent Forms:

Name	Version	Date Created	Date Modified	Date Approved

There are no items to display

Approved Surveys, etc.:

Name	Version	Date Created	Date Modified	Date Approved
Questionnaire #1 - No Genetic Testing 07102020.docx(0.01)	0.01	7/13/2020 2:51 PM	7/13/2020 2:51 PM	8/11/2020 3:10 PM
Questionnaire #1 - Genetic Testing 07102020.docx(0.01)	0.01	7/13/2020 2:51 PM	7/13/2020 2:51 PM	8/11/2020 3:10 PM
Questionnaire #2 - No Genetic Testing 07102020.docx(0.01)	0.01	7/13/2020 2:51 PM	7/13/2020 2:51 PM	8/11/2020 3:10 PM
Questionnaire #2 - Genetic Testing 07102020.docx(0.01)	0.01	7/13/2020 2:51 PM	7/13/2020 2:51 PM	8/11/2020 3:10 PM
Questionnaire -1 - Genetic Testing 05182020_Clean_Spa_Final-Amend.docx(0.01)	0.01	9/1/2020 3:24 PM	9/1/2020 3:24 PM	9/15/2020 10:58 AM
Questionnaire - 2 - No Genetic Testing 05182020_Clean_Spa_Final-Amend.docx(0.01)	0.01	9/1/2020 3:24 PM	9/1/2020 3:24 PM	9/15/2020 10:58 AM
Questionnaire - 1 - No Genetic Testing 05182020_Clean_Spa_Final-Amend.docx(0.01)	0.01	9/1/2020 3:24 PM	9/1/2020 3:24 PM	9/15/2020 10:58 AM
Questionnaire - 2 - Genetic testing 05182020_Clean_Spa_Final-Amend.docx(0.01)	0.01	9/1/2020 3:24 PM	9/1/2020 3:24 PM	9/15/2020 10:58 AM

Updated Surveys, etc.:

Name	Version	Date Created	Date Modified	Date Approved

There are no items to display

Approved Company Protocol:

Name	Version	Date Created	Date Modified	Date Approved

There are no items to display

Updated Company Protocol:

Name	Version	Date Created	Date Modified	Date Approved

There are no items to display

Approved Investigational Brochure:

Name	Version	Date Created	Date Modified	Date Approved

There are no items to display

Updated Investigational Brochure:

Name	Version	Date Created	Date Modified	Date Approved

There are no items to display

Approved Grant Application:

Name	Version	Date Created	Date Modified	Date Approved
Grant application(0.01)	0.01	1/20/2019 3:03 PM	1/20/2019 3:03 PM	6/5/2019 2:56 PM

Updated Grant Application:

Name	Version	Date Created	Date Modified	Date Approved

There are no items to display

Approved Literature/Cited References:

Name	Version	Date Created	Date Modified	Date Approved
Background and Introduction References(0.01)	0.01	2/22/2019 8:32 AM	2/22/2019 8:32 AM	6/5/2019 2:56 PM

Updated Literature/Cited References:

Name	Version	Date Created	Date Modified	Date Approved

There are no items to display

Current PI Scholarly Record (CV/Resume):

Name	Version	Date Created	Date Modified	Date Approved
Kaphingst CV(0.04)	0.04	4/2/2015 12:18 PM	8/27/2018 3:25 PM	

Updated PI Scholarly Record (CV/Resume):

Name	Version	Date Created	Date Modified	Date Approved
Kaphingst CV(0.04)	0.04	4/2/2015 12:18 PM	8/27/2018 3:25 PM	

Current Faculty Sponsor Scholarly Record (CV/Resume):

Name	Version	Date Created	Date Modified	Date Approved
There are no items to display				

Updated Faculty Sponsor Scholarly Record (CV/Resume):

Name	Version	Date Created	Date Modified	Date Approved
There are no items to display				

Approved Other Stamped Documents:

Name	Version	Date Created	Date Modified	Date Approved
There are no items to display				

Updated Other Stamped Documents:

Name	Version	Date Created	Date Modified	Date Approved
There are no items to display				

Approved Recruitment Materials, Advertisements, etc.:

Name	Version	Date Created	Date Modified	Date Approved
Q1 invitation-No testing.docx(0.01)	0.01	7/14/2020 3:54 PM	7/14/2020 3:54 PM	8/11/2020 3:10 PM
Q1 invitation-Testing.docx(0.01)	0.01	7/14/2020 3:54 PM	7/14/2020 3:54 PM	8/11/2020 3:10 PM
Q2 Inivitation-No testing.docx(0.01)	0.01	7/14/2020 3:54 PM	7/14/2020 3:54 PM	8/11/2020 3:10 PM
Q2 Invitation-Testing.docx(0.01)	0.01	7/14/2020 3:54 PM	7/14/2020 3:54 PM	8/11/2020 3:10 PM
Q1 Mail Coverletter_Testing_NYU.docx(0.01)	0.01	7/15/2020 2:44 PM	7/15/2020 2:44 PM	8/11/2020 3:10 PM
Q1 Mail Coverletter_No testing_NYU.docx(0.01)	0.01	7/15/2020 2:44 PM	7/15/2020 2:44 PM	8/11/2020 3:10 PM
Q2 Mail Coverletter_Testing_NYU.docx(0.01)	0.01	7/15/2020 2:45 PM	7/15/2020 2:45 PM	8/11/2020 3:10 PM
Q2 Mail Coverletter_No testing_NYU.docx(0.01)	0.01	7/15/2020 2:45 PM	7/15/2020 2:45 PM	8/11/2020 3:10 PM
Q1 mail coverletter-No testing_Utah.docx(0.01)	0.01	7/15/2020 2:45 PM	7/15/2020 2:45 PM	8/11/2020 3:10 PM
Q1 mail coverletter-Testing_Utah.docx(0.01)	0.01	7/15/2020 2:45 PM	7/15/2020 2:45 PM	8/11/2020 3:10 PM
Q2 mail coverletter-Testing_Utah.docx(0.01)	0.01	7/15/2020 2:45 PM	7/15/2020 2:45 PM	8/11/2020 3:10 PM
Q2 mail coverletter-No Testing_Utah.docx(0.01)	0.01	7/15/2020 2:58 PM	7/15/2020 2:58 PM	8/11/2020 3:10 PM
Q1 mail coverletter-No testing_Utah_Spa_Final.docx(0.01)	0.01	9/1/2020 3:25 PM	9/1/2020 3:25 PM	9/15/2020 10:58 AM

Name	Version	Date Created	Date Modified	Date Approved
Q2 Mail Coverletter_No testing_NYU_Spa_Final.docx(0.01)	0.01	9/1/2020 3:25 PM	9/1/2020 3:25 PM	9/15/2020 10:58 AM
Q2 Invitation-Testing_Spa_Final.docx(0.01)	0.01	9/1/2020 3:25 PM	9/1/2020 3:25 PM	9/15/2020 10:58 AM
Q1 invitation-Testing_Spa_Final.docx(0.01)	0.01	9/1/2020 3:25 PM	9/1/2020 3:25 PM	9/15/2020 10:58 AM
Q1 Mail Coverletter_No testing_NYU_Spa_Final.docx(0.01)	0.01	9/1/2020 3:25 PM	9/1/2020 3:25 PM	9/15/2020 10:58 AM
Q2 mail coverletter-Testing_Utah_Spa_Final.docx(0.01)	0.01	9/1/2020 3:25 PM	9/1/2020 3:25 PM	9/15/2020 10:58 AM
Q1 invitation-No testing_Spa_Final.docx(0.01)	0.01	9/1/2020 3:25 PM	9/1/2020 3:25 PM	9/15/2020 10:58 AM
Q1 Mail Coverletter_Testing_NYU_Spa_Final.docx(0.01)	0.01	9/1/2020 3:25 PM	9/1/2020 3:25 PM	9/15/2020 10:58 AM
Q2 mail coverletter-No Testing_Utah_Spa_Final.docx(0.01)	0.01	9/1/2020 3:25 PM	9/1/2020 3:25 PM	9/15/2020 10:58 AM
Q1 mail coverletter-Testing_Utah_Spa_Final.docx(0.01)	0.01	9/1/2020 3:25 PM	9/1/2020 3:25 PM	9/15/2020 10:58 AM
Q2 Inivitation-No testing_Spa_Final.docx(0.01)	0.01	9/1/2020 3:25 PM	9/1/2020 3:25 PM	9/15/2020 10:58 AM
Q2 Mail Coverletter_Testing_NYU_Spa_Final.docx(0.01)	0.01	9/1/2020 3:25 PM	9/1/2020 3:25 PM	9/15/2020 10:58 AM

Updated Recruitment Materials, Advertisements, etc.:

Name Version Date Created Date Modified Date Approved

There are no items to display

Approved Other Documents:

Name	Version	Date Created	Date Modified	Date Approved
Translation certificate for Consent Coverletter(0.01)	0.01	9/1/2020 3:27 PM	9/1/2020 3:27 PM	9/15/2020 10:58 AM
Translation Certificate for Questionnaires and Recruitment Letters(0.01)	0.01	9/1/2020 3:28 PM	9/1/2020 3:28 PM	9/15/2020 10:58 AM

Updated Other Documents:

Name Version Date Created Date Modified Date Approved

There are no items to display

HELP?

8. Instructions and Finish

1. To view errors in this application, select the "Hide/Show Errors" option at the top or bottom of the page. If you have errors on your application, you won't be able to submit it to the IRB.

Changes to the Update Study Application

2. Be sure to make all proposed changes to the Update Study portion of the application by selecting the "Update Study" button located on the left side of the amendment or continuing review workspace, which will be available once you select the "Finish" button at the top or bottom of this page.
3. To attach updated or new documents with this application, you may access the Documents and Attachments page in the Update Study application.
4. If you are proposing changes to any ancillary applications (i.e. RDRC-HUS or RGE), you must access these applications through the Update Study application on the Ancillary Applications page. All changes to ancillary applications must be approved by the corresponding committee prior to IRB approval of the amendment.

Submitting the Completed Continuing Review Application

5. Selecting the �Finish� button alone will NOT submit the application to the IRB. You MUST also select the "Submit" option on the workspace after you've selected the "Finish" button. Only the PI can submit the application to the IRB.
6. If your study has a faculty sponsor: Once the PI submits the application, it will be sent to the faculty sponsor for final approval. The IRB cannot review the study until the faculty sponsor submits the application to the IRB.

IRB_00115509 - CR_2/22/2021 12:04 PM **Created:** 2/22/2021 12:04 PM

PI: Kimberly Kaphingst **Submitted:** 3/16/2021

Title: Broadening the Reach, Impact, and Delivery of Genetic Services

Study Location Questions

NYU School of Medicine

Site Status

What is the enrollment status for this site?
Open to enrollment of new participants

1,920 consent cover letters sent; up to 20,000 EHR records	**Number of currently approved enrollments for this study as a whole**

Enrollment since the beginning of the study at this site:

2**Total number enrolled as of today�s date at this site**

Enrollment since the last continuing review approval at this site:

0**Reported enrollment during the last continuing review**

2**Number enrolled since the last continuing review**

How many non-English speakers have been enrolled?
0

How many participants signed consent forms but did not end up participating in the study?
0

Problems and Events

Have any unanticipated problems or adverse events involving risks to participants occured since the last IRB approval?
○ Yes ◉ **No**

Have there been any complaints about the research since the last IRB review?
○ Yes ◉ **No**

Local HRPP Reporting

Have any significant financial conflicts of interest for study personnel been identified since the last approval that have not been reported to the IRB?
○ Yes ◉ **No**

Have there been any changes to human research protection reviews or approvals from your institution since last approval that have not been reported to the IRB.
○ Yes ◉ **No**

Study Location Questions

University of Utah

Site Status

What is the enrollment status for this site?
Open to enrollment of new participants

1,920 consent cover letters sent; up to 20,000 EHR records **Number of currently approved enrollments for this study as a whole**

Enrollment since the beginning of the study at this site:

34**Total number enrolled as of today�s date at this site**

Enrollment since the last continuing review approval at this site:

0**Reported enrollment during the last continuing review**

34**Number enrolled since the last continuing review**

How many non-English speakers have been enrolled?
0

How many participants signed consent forms but did not end up participating in the study?
0

Problems and Events

Have any unanticipated problems or adverse events involving risks to participants occured since the last IRB approval?
○ Yes ◉ **No**

Have there been any complaints about the research since the last IRB review?
○ Yes ◉ **No**

Electronic RESEARCH INTEGRITY AND COMPLIANCE THE UNIVERSITY OF UTAH

Date: Monday, March 14, 2022 9:30:14 PM

Print Close

IRB_00115509 - CR_12/8/2021 12:59 PM **Created:** 12/8/2021 12:59 PM

PI: Kimberly Kaphingst **Submitted:** 1/11/2022

Title: Broadening the Reach, Impact, and Delivery of Genetic Services

HELP?

1. Status

Overall, what is the enrollment status for this study?
Open to enrollment of new participants

-

IRB_00115509 - CR_12/8/2021 12:59 PM **Created:** 12/8/2021 12:59 PM

PI: Kimberly Kaphingst **Submitted:** 1/11/2022

Title: Broadening the Reach, Impact, and Delivery of Genetic Services

HELP?

2. Progress of Study and Enrollment Information

Overall Study Enrollment Progress

Number of currently approved enrollments for this study as a whole: 1,920 consent cover letters sent; up to 20,000 EHR records

Number enrolled across all sites at last Continuing Review:36

Number enrolled across all sites as of today�s date:457

Number enrolled across all sites since last Continuing Review:421

- **Describe the progress of the study, observations of the participants studied, and reason(s) for continuing the study:**
  Utah began enrollment for their pilot study in April 2020 and enrollment for their full study in November 2020. To date, 225 participants have been invited to participate in Questionnaire #1 and 10 participants have been invited to participate in Questionnaire #2 at this site. Although delayed due to COVID-19, recruitment remains ongoing and enrollment has picked up during 2021.

  NYU began enrollment for their pilot study in Dec 2020 and enrollment for the full study in February 2021. To date, 130 participants have been invited to participate in Questionnaire #1. The technical

implementation was substantially delayed at this site due to COVID-19 but recruitment and enrollment have been successful during 2021 and remains ongoing.

- **What results (preliminary or final) have been obtained from the study? If the study is part of a multi-center trial, this should be stated and any available results provided. If there are no results that are appropriate to report to the IRB at this time, this should be stated and explained:**
  We will be conducting analysis after accrual is complete.

  Please attach any abstracts or publications:

Name	Version	Date Created	Date Modified	Date Approved

  There are no items to display

- **Describe participant enrollment and its effect on the study design and scientific merit. If under-accrued, describe any plans for increasing enrollment during the next approval period. If a multicenter trial, also discuss enrollment at other sites.**
  Although significantly delayed due to COVID-19 and technical implementation hurdles, enrollment at both sites during 2021 has been successful and on track with the projected study timeline.

IRB_00115509 - CR_12/8/2021 12:59 PM **Created:** 12/8/2021 12:59 PM

PI: Kimberly Kaphingst **Submitted:** 1/11/2022

Title: Broadening the Reach, Impact, and Delivery of Genetic Services

HELP?

Progress of Sites and Enrollment

Select each site to update the enrollment information.

List of Study Controlled Locations

	Site Name	Investigator's Name	Enrollments since Last CR	Enrollments as of Today	Non English Speakers	Withdrawn
View	NYU School of Medicine	Meena Sigireddi	177	179	0	0
View	University of Utah	Kimberly Kaphingst	244	278	7	0

IRB_00115509 - CR_12/8/2021 12:59 PM **Created:** 12/8/2021 12:59 PM

PI: Kimberly Kaphingst **Submitted:** 1/11/2022

Title: Broadening the Reach, Impact, and Delivery of Genetic Services

HELP?

3. Problems & Events

1. **Provide narrative summary of any available information regarding adverse events which have occurred since the last IRB review.**
   - There have been no adverse events
2. **Have any additional risks to participation in this study been identified (from any site or other relevant source) since last approval? If reports or amendments identifying additional risks have been submitted since last approval, please mark �yes�.**
   - ○ Yes ◉ **No**

IRB_00115509 - CR_12/8/2021 12:59 PM **Created:** 12/8/2021 12:59 PM

PI: Kimberly Kaphingst **Submitted:** 1/11/2022

Title: Broadening the Reach, Impact, and Delivery of Genetic Services

HELP?

5. Amendments

A list of amendments associated with this study since the last approval date.

Name	Date Submitted	IRB #
AM_Spanish enrollment	6/16/2021	AM_00041845
AM_NYU PI Change	8/24/2021	AM_00042488

1. **Are there any changes to be made now? (Including scientific design, procedures, participant population, or any minor changes.)**
   ◉ **Yes** ○ No

IRB_00115509 - CR_12/8/2021 12:59 PM **Created:** 12/8/2021 12:59 PM

PI: Kimberly Kaphingst **Submitted:** 1/11/2022

Title: Broadening the Reach, Impact, and Delivery of Genetic Services

HELP?

Amendment Description

1. **Type of Amendment (check all that apply):**
   Administrative changes:
   Changes to study procedures:
2. **What changes are being made? List and number each change, grouping similar changes together.**

   1. Administrative changes:

Remove Kadyn Kimball, Priyanka Kanth, and Kenneth Boucher

2. Changes to study procedures:

Change language to replace the Cancer Biostatistics Shared Resource with NYU for where data analyses will be conducted.

3. **Describe the reason for each of the changes described above. List and number the reasons according to the list above.**

   1. Administrative changes:

   Kadyn Kimball and Priyanka Kanth are no longer at HCI.

   Kenneth Boucher is being removed because data analysis is being done at NYU.

   2. Changes to study procedures:

   Data analysis will be conducted at NYU, not Utah.

4. **How does each change described above effect participants? List and number the effects according to the list above.**

   1. Administrative changes will have no effect on participants

   2. Changes to study procedures will have no effect on participants

5. **Will the modification(s), in the opinion of the local PI, increase or decrease the risk to participants?**
   Neither

   If the risk changes, provide justification:

6. **How will enrolled participants (current and past) be notified of this change?**
   Participants will not be notified

   If Other, please explain:

7. **Which approved documents are affected by these changes?**
   There are no items to display

   If Other, please list

8. **Which sections of the Update Study Application are affected by these changes?**
   1. Contacts and title; #2 and #3
   2. Study Location and Sponsors; #1 - University of Utah
   4. Study Information; Consent Process #1
   8. R&R #1 and 3
   HCI PRMC; Site staff

9. **Select all study locations that are affected by these changes.**
   Prior Approved Study Controlled Locations

Site Name	Investigator Name	Covered Entity
☐ NYU School of Medicine	Meena Sigireddi	Study procedures will be conducted outside a HIPAA Covered Entity at this site (HIPAA Privacy Rule does not apply)
☐ University of Utah	Kaphingst	Study procedures will be conducted within a HIPAA Covered Entity at this site (HIPAA Privacy Rule applies)

New Sites

IRB_00115509 - CR_12/8/2021 12:59 PM **Created:** 12/8/2021 12:59 PM
PI: Kimberly Kaphingst **Submitted:** 1/11/2022
Title: Broadening the Reach, Impact, and Delivery of Genetic Services

HELP?

Amendment Approvals

1. **Is this amendment related to information that meets the IRB reporting policy for unanticipated problems and non-compliance?**
   ○ Yes ◉ **No**

   If yes, a Report Form must also be submitted and then linked to this amendment. Link the related report form to the amendment application by selecting the report form from the list below.

ID	Name	Date Submitted	Status

2. **Investigational Drug Data Form (IDDF) Changes:** ○ Yes ◉ **No**

3. **Are you adding the VA as a site?** ○ Yes ◉ **No**

IRB_00115509 - CR_12/8/2021 12:59 PM **Created:** 12/8/2021 12:59 PM
PI: Kimberly Kaphingst **Submitted:** 1/11/2022
Title: Broadening the Reach, Impact, and Delivery of Genetic Services

HELP?

6. Report Forms

Name	Date Submitted	IRB #

IRB_00115509 - CR_12/8/2021 12:59 PM **Created:** 12/8/2021 12:59 PM
PI: Kimberly Kaphingst **Submitted:** 1/11/2022
Title: Broadening the Reach, Impact, and Delivery of Genetic Services

HELP?

7. Documents and Attachments

Approved eProtocol Summary:

Name	Version	Date Created	Date Modified	Date Approved
ID00000007(0.01)	0.01	3/14/2022 8:57 AM	3/14/2022 8:57 AM	ID00000007

Print View: IRB Draft Protocol Summary

Updated eProtocol Summary:

Name	Version	Date Created	Date Modified	Date Approved

There are no items to display

Approved Consent Forms:

Name	Version	Date Created	Date Modified	Date Approved
Consent Cover Letter V2.0(0.01)	0.01	1/6/2022 11:48 AM	1/6/2022 11:48 AM	3/14/2022 8:57 AM
Consent Cover Letter V2.0_Spanish(0.01)	0.01	1/6/2022 11:48 AM	1/6/2022 11:48 AM	3/14/2022 8:57 AM

Updated Consent Forms:

Name	Version	Date Created	Date Modified	Date Approved

There are no items to display

Approved Parental Permission Forms:

Name	Version	Date Created	Date Modified	Date Approved

There are no items to display

Updated Parental Permission Forms:

Name	Version	Date Created	Date Modified	Date Approved

There are no items to display

Approved Assent Forms:

Name	Version	Date Created	Date Modified	Date Approved

There are no items to display

Updated Assent Forms:

Name	Version	Date Created	Date Modified	Date Approved

There are no items to display

Approved VA Consent Forms:

Name	Version	Date Created	Date Modified	Date Approved

There are no items to display

Updated VA Consent Forms:

Name	Version	Date Created	Date Modified	Date Approved

There are no items to display

Approved Surveys, etc.:

Name	Version	Date Created	Date Modified	Date Approved
Questionnaire #1 - No Genetic Testing 07102020.docx(0.01)	0.01	7/13/2020 2:51 PM	7/13/2020 2:51 PM	8/11/2020 3:10 PM
Questionnaire #1 - Genetic Testing 07102020.docx(0.01)	0.01	7/13/2020 2:51 PM	7/13/2020 2:51 PM	8/11/2020 3:10 PM
Questionnaire #2 - No Genetic Testing 07102020.docx(0.01)	0.01	7/13/2020 2:51 PM	7/13/2020 2:51 PM	8/11/2020 3:10 PM
Questionnaire #2 - Genetic Testing 07102020.docx(0.01)	0.01	7/13/2020 2:51 PM	7/13/2020 2:51 PM	8/11/2020 3:10 PM
Questionnaire -1 - Genetic Testing 05182020_Clean_Spa_Final-Amend.docx(0.01)	0.01	9/1/2020 3:24 PM	9/1/2020 3:24 PM	9/15/2020 10:58 AM
Questionnaire - 2 - No Genetic Testing 05182020_Clean_Spa_Final-Amend.docx(0.01)	0.01	9/1/2020 3:24 PM	9/1/2020 3:24 PM	9/15/2020 10:58 AM
Questionnaire - 1 - No Genetic Testing 05182020_Clean_Spa_Final-Amend.docx(0.01)	0.01	9/1/2020 3:24 PM	9/1/2020 3:24 PM	9/15/2020 10:58 AM
Questionnaire - 2 - Genetic testing 05182020_Clean_Spa_Final-Amend.docx(0.01)	0.01	9/1/2020 3:24 PM	9/1/2020 3:24 PM	9/15/2020 10:58 AM

Updated Surveys, etc.:

Name	Version	Date Created	Date Modified	Date Approved

There are no items to display

Approved Company Protocol:

Name	Version	Date Created	Date Modified	Date Approved

There are no items to display

Updated Company Protocol:

Name	Version	Date Created	Date Modified	Date Approved

There are no items to display

Approved Investigational Brochure:

Name	Version	Date Created	Date Modified	Date Approved

There are no items to display

Updated Investigational Brochure:

Name	Version	Date Created	Date Modified	Date Approved

There are no items to display

Approved Grant Application:

Name	Version	Date Created	Date Modified	Date Approved
Grant application(0.01)	0.01	1/20/2019 3:03 PM	1/20/2019 3:03 PM	6/5/2019 2:56 PM

Updated Grant Application:

Name	Version	Date Created	Date Modified	Date Approved

There are no items to display

Approved Literature/Cited References:

Name	Version	Date Created	Date Modified	Date Approved
Background and Introduction References(0.01)	0.01	2/22/2019 8:32 AM	2/22/2019 8:32 AM	6/5/2019 2:56 PM

Updated Literature/Cited References:

Name	Version	Date Created	Date Modified	Date Approved

There are no items to display

Current PI Scholarly Record (CV/Resume):

Name	Version	Date Created	Date Modified	Date Approved
Kaphingst CV(0.04)	0.04	4/2/2015 12:18 PM	8/27/2018 3:25 PM	

Updated PI Scholarly Record (CV/Resume):

Name	Version	Date Created	Date Modified	Date Approved
Kaphingst CV(0.04)	0.04	4/2/2015 12:18 PM	8/27/2018 3:25 PM	

Current Faculty Sponsor Scholarly Record (CV/Resume):

Name	Version	Date Created	Date Modified	Date Approved

There are no items to display

Updated Faculty Sponsor Scholarly Record (CV/Resume):

Name	Version	Date Created	Date Modified	Date Approved

There are no items to display

Approved Other Stamped Documents:

Name	Version	Date Created	Date Modified	Date Approved

There are no items to display

Updated Other Stamped Documents:

Name	Version	Date Created	Date Modified	Date Approved

There are no items to display

Approved Recruitment Materials, Advertisements, etc.:

Name	Version	Date Created	Date Modified	Date Approved
Q1 invitation-No testing.docx(0.01)	0.01	7/14/2020 3:54 PM	7/14/2020 3:54 PM	8/11/2020 3:10 PM
Q1 invitation-Testing.docx(0.01)	0.01	7/14/2020 3:54 PM	7/14/2020 3:54 PM	8/11/2020 3:10 PM
Q2 Inivitation-No testing.docx(0.01)	0.01	7/14/2020 3:54 PM	7/14/2020 3:54 PM	8/11/2020 3:10 PM
Q2 Invitation-Testing.docx(0.01)	0.01	7/14/2020 3:54 PM	7/14/2020 3:54 PM	8/11/2020 3:10 PM
Q1 mail coverletter-No testing_Utah.docx(0.01)	0.01	7/15/2020 2:45 PM	7/15/2020 2:45 PM	8/11/2020 3:10 PM
Q1 mail coverletter-Testing_Utah.docx(0.01)	0.01	7/15/2020 2:45 PM	7/15/2020 2:45 PM	8/11/2020 3:10 PM
Q2 mail coverletter-Testing_Utah.docx(0.01)	0.01	7/15/2020 2:45 PM	7/15/2020 2:45 PM	8/11/2020 3:10 PM
Q2 mail coverletter-No Testing_Utah.docx(0.01)	0.01	7/15/2020 2:58 PM	7/15/2020 2:58 PM	8/11/2020 3:10 PM
Q1 mail coverletter-No testing_Utah_Spa_Final.docx(0.01)	0.01	9/1/2020 3:25 PM	9/1/2020 3:25 PM	9/15/2020 10:58 AM
Q2 Invitation-Testing_Spa_Final.docx(0.01)	0.01	9/1/2020 3:25 PM	9/1/2020 3:25 PM	9/15/2020 10:58 AM
Q1 invitation-Testing_Spa_Final.docx(0.01)	0.01	9/1/2020 3:25 PM	9/1/2020 3:25 PM	9/15/2020 10:58 AM
Q2 mail coverletter-Testing_Utah_Spa_Final.docx(0.01)	0.01	9/1/2020 3:25 PM	9/1/2020 3:25 PM	9/15/2020 10:58 AM
Q1 invitation-No testing_Spa_Final.docx(0.01)	0.01	9/1/2020 3:25 PM	9/1/2020 3:25 PM	9/15/2020 10:58 AM
Q2 mail coverletter-No Testing_Utah_Spa_Final.docx(0.01)	0.01	9/1/2020 3:25 PM	9/1/2020 3:25 PM	9/15/2020 10:58 AM
Q1 mail coverletter-Testing_Utah_Spa_Final.docx(0.01)	0.01	9/1/2020 3:25 PM	9/1/2020 3:25 PM	9/15/2020 10:58 AM
Q2 Inivitation-No testing_Spa_Final.docx(0.01)	0.01	9/1/2020 3:25 PM	9/1/2020 3:25 PM	9/15/2020 10:58 AM
20210812_MS_CLEAN_Q1 Mail Coverletter_Testing_NYU.docx(0.01)	0.01	8/16/2021 8:15 AM	8/16/2021 8:15 AM	8/25/2021 10:25 AM
20210812_MS_CLEAN_Q2 Mail Coverletter_Testing_NYU.docx(0.01)	0.01	8/16/2021 8:15 AM	8/16/2021 8:15 AM	8/25/2021 10:25 AM
20210812_MS_CLEAN_Q1 Mail Coverletter_Testing_NYU_Spa_Final.docx(0.01)	0.01	8/16/2021 8:15 AM	8/16/2021 8:15 AM	8/25/2021 10:25 AM
20210812_MS_CLEAN_Q2 Mail Coverletter_Testing_NYU_Spa_Final.docx(0.01)	0.01	8/16/2021 8:15 AM	8/16/2021 8:15 AM	8/25/2021 10:25 AM
20210812_MS_CLEAN_Q1 Mail Coverletter_No testing_NYU.docx(0.01)	0.01	8/16/2021 8:16 AM	8/16/2021 8:16 AM	8/25/2021 10:25 AM
20210812_CLEAN_Q2 Mail Coverletter_No testing_NYU.docx(0.01)	0.01	8/16/2021 8:16 AM	8/16/2021 8:16 AM	8/25/2021 10:25 AM
20210812_MS_CLEAN_Q1 Mail Coverletter_No testing_NYU_Spa_Final.docx(0.01)	0.01	8/16/2021 8:16 AM	8/16/2021 8:16 AM	8/25/2021 10:25 AM

Name	Version	Date Created	Date Modified	Date Approved
20210812_MS_CLEAN_Q2 Mail Coverletter_No testing_NYU_Spa_Final.docx(0.01)	0.01	8/16/2021 8:16 AM	8/16/2021 8:16 AM	8/25/2021 10:25 AM

Updated Recruitment Materials, Advertisements, etc.:

Name	Version	Date Created	Date Modified	Date Approved

There are no items to display

Approved Other Documents:

Name	Version	Date Created	Date Modified	Date Approved
Translation certificate for Consent Coverletter(0.01)	0.01	9/1/2020 3:27 PM	9/1/2020 3:27 PM	9/15/2020 10:58 AM
Translation Certificate for Questionnaires and Recruitment Letters(0.01)	0.01	9/1/2020 3:28 PM	9/1/2020 3:28 PM	9/15/2020 10:58 AM
Sigireddi CITI Biomed Research(0.01)	0.01	8/23/2021 10:01 AM	8/23/2021 10:01 AM	8/25/2021 10:25 AM
Sigireddi CITI Clinical Practice(0.01)	0.01	8/23/2021 10:02 AM	8/23/2021 10:02 AM	8/25/2021 10:25 AM
Sigireddi Coi(0.01)	0.01	8/23/2021 10:02 AM	8/23/2021 10:02 AM	8/25/2021 10:25 AM
Sigireddi CV(0.01)	0.01	8/23/2021 10:03 AM	8/23/2021 10:03 AM	8/25/2021 10:25 AM

Updated Other Documents:

Name	Version	Date Created	Date Modified	Date Approved

There are no items to display

IRB_00115509 - CR_12/8/2021 12:59 PM **Created:** 12/8/2021 12:59 PM

PI: Kimberly Kaphingst **Submitted:** 1/11/2022

Title: Broadening the Reach, Impact, and Delivery of Genetic Services

HELP?

8. Instructions and Finish

1. To view errors in this application, select the "Validate" option at the top-left of the page. If you have errors on your application, you won't be able to submit it to the IRB.

Changes to the Update Study Application

2. Be sure to make all proposed changes to the Update Study portion of the application by selecting the "Update Study" button located on the left side of the amendment or continuing review workspace, which will be available once you select the "Finish" button at the top or bottom of this page.
3. To attach updated or new documents with this application, you may access the Documents and Attachments page in the Update Study application.
4. If you are proposing changes to any ancillary applications (i.e. RDRC-HUS or RGE), you must access these applications through the Update Study application on the Ancillary Applications page.

All changes to ancillary applications must be approved by the corresponding committee prior to IRB approval of the amendment.

Submitting the Completed Continuing Review Application

5. Selecting the �Finish� button alone will NOT submit the application to the IRB. You MUST also select the "Submit" option on the workspace after you've selected the "Finish" button. Only the PI can submit the application to the IRB.
6. If your study has a faculty sponsor: Once the PI submits the application, it will be sent to the faculty sponsor for final approval. The IRB cannot review the study until the faculty sponsor submits the application to the IRB.

IRB_00115509 - CR_12/8/2021 12:59 PM **Created:** 12/8/2021 12:59 PM

PI: Kimberly Kaphingst **Submitted:** 1/11/2022

Title: Broadening the Reach, Impact, and Delivery of Genetic Services

Study Location Questions

NYU School of Medicine

Site Status

What is the enrollment status for this site?
Open to enrollment of new participants

1,920 consent cover letters sent; up to 20,000 EHR records **Number of currently approved enrollments for this study as a whole**

Enrollment since the beginning of the study at this site:

179**Total number enrolled as of today�s date at this site**

Enrollment since the last continuing review approval at this site:

2**Reported enrollment during the last continuing review**

177**Number enrolled since the last continuing review**

How many non-English speakers have been enrolled?

0

How many participants signed consent forms but did not end up participating in the study?
0

Problems and Events

Have any unanticipated problems or adverse events involving risks to participants occured since the last IRB approval?
○ Yes ◉ **No**

Have there been any complaints about the research since the last IRB review?
○ Yes ◉ **No**

Local HRPP Reporting

Have any significant financial conflicts of interest for study personnel been identified since the last approval that have not been reported to the IRB?
○ Yes ◉ **No**

Have there been any changes to human research protection reviews or approvals from your institution since last approval that have not been reported to the IRB.
○ Yes ◉ **No**

IRB_00115509 - CR_12/8/2021 12:59 PM **Created:** 12/8/2021 12:59 PM
PI: Kimberly Kaphingst **Submitted:** 1/11/2022
Title: Broadening the Reach, Impact, and Delivery of Genetic Services

Study Location Questions

University of Utah

Site Status

What is the enrollment status for this site?
Open to enrollment of new participants

Number of currently approved enrollments for this study as a whole
1,920 consent cover letters sent; up to 20,000 EHR records

Enrollment since the beginning of the study at this site:

278**Total number enrolled as of today�s date at this site**

Enrollment since the last continuing review approval at this site:

34**Reported enrollment during the last continuing review**

244**Number enrolled since the last continuing review**

How many non-English speakers have been enrolled?
7

Which non-English languages were used in the enrollment process since the last review?

Yes **Spanish**

How many Spanish fully translated long forms were used? 7 How many Spanish short forms were used? 0

☐ **Mandarin**

☐ **Nepali**

☐ **Vietnamese**

☐ **Russian**

☐ **Other**

How many participants signed consent forms but did not end up participating in the study?
0

Problems and Events

Have any unanticipated problems or adverse events involving risks to participants occured since the last IRB approval?

○ Yes ◉ **No**

Have there been any complaints about the research since the last IRB review?

○ Yes ◉ **No**

Electronic
RESEARCH INTEGRITY AND COMPLIANCE
THE UNIVERSITY OF UTAH

Date: Monday, February 13, 2023 9:30:25 PM

Print Close

IRB_00115509 - CR_12/15/2022 9:44 AM **Created:** 12/15/2022 9:44 AM

PI: Kimberly Kaphingst **Submitted:** 1/19/2023

Title: Broadening the Reach, Impact, and Delivery of Genetic Services

HELP?

1. Status

Overall, what is the enrollment status for this study?
Open to enrollment of new participants

-

IRB_00115509 - CR_12/15/2022 9:44 AM **Created:** 12/15/2022 9:44 AM

PI: Kimberly Kaphingst **Submitted:** 1/19/2023

Title: Broadening the Reach, Impact, and Delivery of Genetic Services

HELP?

2. Progress of Study and Enrollment Information

Overall Study Enrollment Progress

Number of currently approved enrollments for this study as a whole: 1,920 consent cover letters sent; up to 20,000 EHR records

Number enrolled across all sites at last Continuing Review:457

Number enrolled across all sites as of today�s date:794

Number enrolled across all sites since last Continuing Review:337

- **Describe the progress of the study, observations of the participants studied, and reason(s) for continuing the study:**
  Utah began enrollment for their pilot study in April 2020 and enrollment for their full study in November 2020. To date, appox. 450 participants have been invited to participate in Questionnaire #1 and 100 participants have been invited to participate in Questionnaire #2 at this site. Although delayed due to COVID-19, recruitment remains ongoing and enrollment has picked up during 2022.

  NYU began enrollment for their pilot study in Dec 2020 and enrollment for the full study in February 2021. To date, 480 participants have been invited to participate in Questionnaire #1. The technical

implementation was substantially delayed at this site due to COVID-19 but recruitment and enrollment have been successful during 2022 and remains ongoing.

- **What results (preliminary or final) have been obtained from the study? If the study is part of a multi-center trial, this should be stated and any available results provided. If there are no results that are appropriate to report to the IRB at this time, this should be stated and explained:**
  We will be conducting analysis after accrual is complete.

  Please attach any abstracts or publications:

Name	Version	Date Created	Date Modified	Date Approved

  There are no items to display

- **Describe participant enrollment and its effect on the study design and scientific merit. If under-accrued, describe any plans for increasing enrollment during the next approval period. If a multicenter trial, also discuss enrollment at other sites.**
  Although significantly delayed due to COVID-19 and technical implementation hurdles, enrollment at both sites during 2021 & 2022 has been successful and on track with the projected study timeline.

  Enrollment for this continuing review was defined as those who had completed only research procedures (i.e., research questionnaires) at both sites. We realized that in the prior continuing review, the number reported as enrolled was the number who completed both clinical and research procedures rather than research procedures only. This is why ERICA seems to have a negative number within the progress of the sites within this application. At the last CR the number enrolled should have been the 157 individuals completing research procedures. Between sites, we have enrolled 150 participants since the last CR, for a total enrolled of 307 between sites.

IRB_00115509 - CR_12/15/2022 9:44 AM **Created:** 12/15/2022 9:44 AM

PI: Kimberly Kaphingst **Submitted:** 1/19/2023

Title: Broadening the Reach, Impact, and Delivery of Genetic Services

HELP?

Progress of Sites and Enrollment

Select each site to update the enrollment information.

List of Study Controlled Locations

	Site Name	Investigator's Name	Enrollments since Last CR	Enrollments as of Today	Non English Speakers	Withdrawn
View	NYU School of Medicine	Meena Sigireddi	-108	71	0	0
View	University of Utah	Kimberly Kaphingst	-42	236	2	0

IRB_00115509 - CR_12/15/2022 9:44 AM **Created:** 12/15/2022 9:44 AM
PI: Kimberly Kaphingst **Submitted:** 1/19/2023
Title: Broadening the Reach, Impact, and Delivery of Genetic Services

HELP?

3. Problems & Events

1. **Provide narrative summary of any available information regarding adverse events which have occurred since the last IRB review.**
   - N/A there have been no adverse events
2. **Have any additional risks to participation in this study been identified (from any site or other relevant source) since last approval? If reports or amendments identifying additional risks have been submitted since last approval, please mark �yes�.**
   - ○ Yes ◉ **No**

IRB_00115509 - CR_12/15/2022 9:44 AM **Created:** 12/15/2022 9:44 AM
PI: Kimberly Kaphingst **Submitted:** 1/19/2023
Title: Broadening the Reach, Impact, and Delivery of Genetic Services

HELP?

5. Amendments

A list of amendments associated with this study since the last approval date.

Name	Date Submitted	IRB #

1. **Are there any changes to be made now? (Including scientific design, procedures, participant population, or any minor changes.)**
   ○ Yes ◉ **No**

IRB_00115509 - CR_12/15/2022 9:44 AM **Created:** 12/15/2022 9:44 AM
PI: Kimberly Kaphingst **Submitted:** 1/19/2023
Title: Broadening the Reach, Impact, and Delivery of Genetic Services

HELP?

6. Report Forms

Name	Date Submitted	IRB #

IRB_00115509 - CR_12/15/2022 9:44 AM **Created:** 12/15/2022 9:44 AM
PI: Kimberly Kaphingst **Submitted:** 1/19/2023
Title: Broadening the Reach, Impact, and Delivery of Genetic Services

HELP?

7. Documents and Attachments

Approved eProtocol Summary:

Name	Version	Date Created	Date Modified	Date Approved
ID00000007(0.01)	0.01	2/13/2023 7:43 AM	2/13/2023 7:43 AM	ID00000007

Print View: IRB Draft Protocol Summary

Updated eProtocol Summary:

Name	Version	Date Created	Date Modified	Date Approved

There are no items to display

Approved Consent Forms:

Name	Version	Date Created	Date Modified	Date Approved
Consent Cover Letter V2.0_CR2023.docx(0.01)	0.01	12/15/2022 10:22 AM	12/15/2022 10:22 AM	2/13/2023 7:43 AM
Consent Cover Letter V2.0_CR2023_Spanish.docx(0.01)	0.01	12/15/2022 10:22 AM	12/15/2022 10:22 AM	2/13/2023 7:43 AM

Updated Consent Forms:

Name	Version	Date Created	Date Modified	Date Approved

There are no items to display

Approved Parental Permission Forms:

Name	Version	Date Created	Date Modified	Date Approved

There are no items to display

Updated Parental Permission Forms:

Name	Version	Date Created	Date Modified	Date Approved

There are no items to display

Approved Assent Forms:

Name	Version	Date Created	Date Modified	Date Approved

There are no items to display

Updated Assent Forms:

Name	Version	Date Created	Date Modified	Date Approved

There are no items to display

Approved VA Consent Forms:

Name	Version	Date Created	Date Modified	Date Approved

There are no items to display

Updated VA Consent Forms:

Name	Version	Date Created	Date Modified	Date Approved

There are no items to display

Approved Surveys, etc.:

Name	Version	Date Created	Date Modified	Date Approved
Questionnaire #1 - No Genetic Testing 07102020.docx(0.01)	0.01	7/13/2020 2:51 PM	7/13/2020 2:51 PM	8/11/2020 3:10 PM
Questionnaire #1 - Genetic Testing 07102020.docx(0.01)	0.01	7/13/2020 2:51 PM	7/13/2020 2:51 PM	8/11/2020 3:10 PM
Questionnaire #2 - No Genetic Testing 07102020.docx(0.01)	0.01	7/13/2020 2:51 PM	7/13/2020 2:51 PM	8/11/2020 3:10 PM
Questionnaire #2 - Genetic Testing 07102020.docx(0.01)	0.01	7/13/2020 2:51 PM	7/13/2020 2:51 PM	8/11/2020 3:10 PM
Questionnaire -1 - Genetic Testing 05182020_Clean_Spa_Final-Amend.docx(0.01)	0.01	9/1/2020 3:24 PM	9/1/2020 3:24 PM	9/15/2020 10:58 AM
Questionnaire - 2 - No Genetic Testing 05182020_Clean_Spa_Final-Amend.docx(0.01)	0.01	9/1/2020 3:24 PM	9/1/2020 3:24 PM	9/15/2020 10:58 AM
Questionnaire - 1 - No Genetic Testing 05182020_Clean_Spa_Final-Amend.docx(0.01)	0.01	9/1/2020 3:24 PM	9/1/2020 3:24 PM	9/15/2020 10:58 AM
Questionnaire - 2 - Genetic testing 05182020_Clean_Spa_Final-Amend.docx(0.01)	0.01	9/1/2020 3:24 PM	9/1/2020 3:24 PM	9/15/2020 10:58 AM

Updated Surveys, etc.:

Name	Version	Date Created	Date Modified	Date Approved

There are no items to display

Approved Company Protocol:

Name	Version	Date Created	Date Modified	Date Approved

There are no items to display

Updated Company Protocol:

Name	Version	Date Created	Date Modified	Date Approved

There are no items to display

Approved Investigational Brochure:

Name	Version	Date Created	Date Modified	Date Approved

There are no items to display

Updated Investigational Brochure:

Name	Version	Date Created	Date Modified	Date Approved

There are no items to display

Approved Grant Application:

Name	Version	Date Created	Date Modified	Date Approved
Grant application(0.01)	0.01	1/20/2019 3:03 PM	1/20/2019 3:03 PM	6/5/2019 2:56 PM

Updated Grant Application:

Name	Version	Date Created	Date Modified	Date Approved

There are no items to display

Approved Literature/Cited References:

Name	Version	Date Created	Date Modified	Date Approved
Background and Introduction References(0.01)	0.01	2/22/2019 8:32 AM	2/22/2019 8:32 AM	6/5/2019 2:56 PM

Updated Literature/Cited References:

Name	Version	Date Created	Date Modified	Date Approved

There are no items to display

Current PI Scholarly Record (CV/Resume):

Name	Version	Date Created	Date Modified	Date Approved
Kaphingst CV(0.04)	0.04	4/2/2015 12:18 PM	8/27/2018 3:25 PM	

Updated PI Scholarly Record (CV/Resume):

Name	Version	Date Created	Date Modified	Date Approved
Kaphingst CV(0.04)	0.04	4/2/2015 12:18 PM	8/27/2018 3:25 PM	

Current Faculty Sponsor Scholarly Record (CV/Resume):

Name	Version	Date Created	Date Modified	Date Approved

There are no items to display

Updated Faculty Sponsor Scholarly Record (CV/Resume):

Name	Version	Date Created	Date Modified	Date Approved

There are no items to display

Approved Other Stamped Documents:

Name	Version	Date Created	Date Modified	Date Approved

There are no items to display

Updated Other Stamped Documents:

Name	Version	Date Created	Date Modified	Date Approved

There are no items to display

Approved Recruitment Materials, Advertisements, etc.:

Name	Version	Date Created	Date Modified	Date Approved
Q1 invitation-No testing.docx(0.01)	0.01	7/14/2020 3:54 PM	7/14/2020 3:54 PM	8/11/2020 3:10 PM
Q1 invitation-Testing.docx(0.01)	0.01	7/14/2020 3:54 PM	7/14/2020 3:54 PM	8/11/2020 3:10 PM
Q2 Inivitation-No testing.docx(0.01)	0.01	7/14/2020 3:54 PM	7/14/2020 3:54 PM	8/11/2020 3:10 PM
Q2 Invitation-Testing.docx(0.01)	0.01	7/14/2020 3:54 PM	7/14/2020 3:54 PM	8/11/2020 3:10 PM
Q1 mail coverletter-No testing_Utah.docx(0.01)	0.01	7/15/2020 2:45 PM	7/15/2020 2:45 PM	8/11/2020 3:10 PM
Q1 mail coverletter-Testing_Utah.docx(0.01)	0.01	7/15/2020 2:45 PM	7/15/2020 2:45 PM	8/11/2020 3:10 PM
Q2 mail coverletter-Testing_Utah.docx(0.01)	0.01	7/15/2020 2:45 PM	7/15/2020 2:45 PM	8/11/2020 3:10 PM
Q2 mail coverletter-No Testing_Utah.docx(0.01)	0.01	7/15/2020 2:58 PM	7/15/2020 2:58 PM	8/11/2020 3:10 PM
Q1 mail coverletter-No testing_Utah_Spa_Final.docx(0.01)	0.01	9/1/2020 3:25 PM	9/1/2020 3:25 PM	9/15/2020 10:58 AM
Q2 Invitation-Testing_Spa_Final.docx(0.01)	0.01	9/1/2020 3:25 PM	9/1/2020 3:25 PM	9/15/2020 10:58 AM
Q1 invitation-Testing_Spa_Final.docx(0.01)	0.01	9/1/2020 3:25 PM	9/1/2020 3:25 PM	9/15/2020 10:58 AM
Q2 mail coverletter-Testing_Utah_Spa_Final.docx(0.01)	0.01	9/1/2020 3:25 PM	9/1/2020 3:25 PM	9/15/2020 10:58 AM
Q1 invitation-No testing_Spa_Final.docx(0.01)	0.01	9/1/2020 3:25 PM	9/1/2020 3:25 PM	9/15/2020 10:58 AM
Q2 mail coverletter-No Testing_Utah_Spa_Final.docx(0.01)	0.01	9/1/2020 3:25 PM	9/1/2020 3:25 PM	9/15/2020 10:58 AM
Q1 mail coverletter-Testing_Utah_Spa_Final.docx(0.01)	0.01	9/1/2020 3:25 PM	9/1/2020 3:25 PM	9/15/2020 10:58 AM
Q2 Inivitation-No testing_Spa_Final.docx(0.01)	0.01	9/1/2020 3:25 PM	9/1/2020 3:25 PM	9/15/2020 10:58 AM
20210812_MS_CLEAN_Q1 Mail Coverletter_Testing_NYU.docx(0.01)	0.01	8/16/2021 8:15 AM	8/16/2021 8:15 AM	8/25/2021 10:25 AM
20210812_MS_CLEAN_Q2 Mail Coverletter_Testing_NYU.docx(0.01)	0.01	8/16/2021 8:15 AM	8/16/2021 8:15 AM	8/25/2021 10:25 AM
20210812_MS_CLEAN_Q1 Mail Coverletter_Testing_NYU_Spa_Final.docx(0.01)	0.01	8/16/2021 8:15 AM	8/16/2021 8:15 AM	8/25/2021 10:25 AM
20210812_MS_CLEAN_Q2 Mail Coverletter_Testing_NYU_Spa_Final.docx(0.01)	0.01	8/16/2021 8:15 AM	8/16/2021 8:15 AM	8/25/2021 10:25 AM
20210812_MS_CLEAN_Q1 Mail Coverletter_No testing_NYU.docx(0.01)	0.01	8/16/2021 8:16 AM	8/16/2021 8:16 AM	8/25/2021 10:25 AM
20210812_CLEAN_Q2 Mail Coverletter_No testing_NYU.docx(0.01)	0.01	8/16/2021 8:16 AM	8/16/2021 8:16 AM	8/25/2021 10:25 AM

Name	Version	Date Created	Date Modified	Date Approved
20210812_MS_CLEAN_Q1 Mail Coverletter_No testing_NYU_Spa_Final.docx(0.01)	0.01	8/16/2021 8:16 AM	8/16/2021 8:16 AM	8/25/2021 10:25 AM
20210812_MS_CLEAN_Q2 Mail Coverletter_No testing_NYU_Spa_Final.docx(0.01)	0.01	8/16/2021 8:16 AM	8/16/2021 8:16 AM	8/25/2021 10:25 AM

Updated Recruitment Materials, Advertisements, etc.:

Name Version Date Created Date Modified Date Approved

There are no items to display

Approved Other Documents:

Name	Version	Date Created	Date Modified	Date Approved
Translation certificate for Consent Coverletter(0.01)	0.01	9/1/2020 3:27 PM	9/1/2020 3:27 PM	9/15/2020 10:58 AM
Translation Certificate for Questionnaires and Recruitment Letters(0.01)	0.01	9/1/2020 3:28 PM	9/1/2020 3:28 PM	9/15/2020 10:58 AM
Sigireddi CITI Biomed Research(0.01)	0.01	8/23/2021 10:01 AM	8/23/2021 10:01 AM	8/25/2021 10:25 AM
Sigireddi CITI Clinical Practice(0.01)	0.01	8/23/2021 10:02 AM	8/23/2021 10:02 AM	8/25/2021 10:25 AM
Sigireddi Coi(0.01)	0.01	8/23/2021 10:02 AM	8/23/2021 10:02 AM	8/25/2021 10:25 AM
Sigireddi CV(0.01)	0.01	8/23/2021 10:03 AM	8/23/2021 10:03 AM	8/25/2021 10:25 AM

Updated Other Documents:

Name Version Date Created Date Modified Date Approved

There are no items to display

IRB_00115509 - CR_12/15/2022 9:44 AM **Created:** 12/15/2022 9:44 AM

PI: Kimberly Kaphingst **Submitted:** 1/19/2023

Title: Broadening the Reach, Impact, and Delivery of Genetic Services

HELP?

8. Instructions and Finish

1. To view errors in this application, select the "Validate" option at the top-left of the page. If you have errors on your application, you won't be able to submit it to the IRB.

Changes to the Update Study Application

2. Be sure to make all proposed changes to the Update Study portion of the application by selecting the "Update Study" button located on the left side of the amendment or continuing review workspace, which will be available once you select the "Finish" button at the top or bottom of this page.
3. To attach updated or new documents with this application, you may access the Documents and Attachments page in the Update Study application.

4. If you are proposing changes to any ancillary applications (i.e. RDRC-HUS or RGE), you must access these applications through the Update Study application on the Ancillary Applications page. All changes to ancillary applications must be approved by the corresponding committee prior to IRB approval of the amendment.

Submitting the Completed Continuing Review Application

5. Selecting the �Finish� button alone will NOT submit the application to the IRB. You MUST also select the "Submit" option on the workspace after you've selected the "Finish" button. Only the PI can submit the application to the IRB.
6. If your study has a faculty sponsor: Once the PI submits the application, it will be sent to the faculty sponsor for final approval. The IRB cannot review the study until the faculty sponsor submits the application to the IRB.

IRB_00115509 - CR_12/15/2022 9:44 AM **Created:** 12/15/2022 9:44 AM

PI: Kimberly Kaphingst **Submitted:** 1/19/2023

Title: Broadening the Reach, Impact, and Delivery of Genetic Services

Study Location Questions

NYU School of Medicine

Site Status

What is the enrollment status for this site?
Open to enrollment of new participants

1,920 consent cover letters sent; up to 20,000 EHR records **Number of currently approved enrollments for this study as a whole**

960**Number of currently approved enrollments for this study location**

Enrollment since the beginning of the study at this site:

71**Total number enrolled as of today�s date at this site**

From the beginning of the study, how many non-English speakers have been enrolled?
0

From the beginning of the study, how many participants signed consent forms but did not end up participating in the study?
0

Enrollment since the last continuing review approval at this site:

179**Reported enrollment during the last continuing review**

-108**Number enrolled since the last continuing review**

Problems and Events

Have any unanticipated problems or adverse events involving risks to participants occured since the last IRB approval?

○ Yes ◉ **No**

Have there been any complaints about the research since the last IRB review?

○ Yes ◉ **No**

Local HRPP Reporting

Have any significant financial conflicts of interest for study personnel been identified since the last approval that have not been reported to the IRB?

○ Yes ◉ **No**

Have there been any changes to human research protection reviews or approvals from your institution since last approval that have not been reported to the IRB.

○ Yes ◉ **No**

IRB_00115509 - CR_12/15/2022 9:44 AM **Created:** 12/15/2022 9:44 AM

PI: Kimberly Kaphingst **Submitted:** 1/19/2023

Title: Broadening the Reach, Impact, and Delivery of Genetic Services

Study Location Questions

University of Utah

Site Status

What is the enrollment status for this site?

Open to enrollment of new participants

1,920 consent cover letters sent; up to 20,000 EHR records **Number of currently approved enrollments for this study as a whole**

960**Number of currently approved enrollments for this study location**

Enrollment since the beginning of the study at this site:

236**Total number enrolled as of today�s date at this site**

From the beginning of the study, how many non-English speakers have been enrolled?
2

Which non-English languages were used in the enrollment process since the last review?

Yes **Spanish**

How many Spanish fully translated long forms were used? 2 How many Spanish short forms were used? 0

☐ **Mandarin**

☐ **Nepali**

☐ **Vietnamese**

☐ **Russian**

☐ **Other**

From the beginning of the study, how many participants signed consent forms but did not end up participating in the study?
0

Enrollment since the last continuing review approval at this site:

278**Reported enrollment during the last continuing review**

-42**Number enrolled since the last continuing review**

Problems and Events

Have any unanticipated problems or adverse events involving risks to participants occured since the last IRB approval?

○ Yes ◉ **No**

Have there been any complaints about the research since the last IRB review?

○ Yes ◉ **No**

Electronic RESEARCH INTEGRITY AND COMPLIANCE THE UNIVERSITY OF UTAH

Date: Friday, February 2, 2024 9:30:05 PM

Print Close

IRB_00115509 - CR_12/15/2023 4:09 PM **Created:** 12/15/2023 4:09 PM

PI: Kimberly Kaphingst **Submitted:** 1/4/2024

Title: Broadening the Reach, Impact, and Delivery of Genetic Services

HELP?

1. Status

Overall, what is the enrollment status for this study?
Open to enrollment of new participants

-

IRB_00115509 - CR_12/15/2023 4:09 PM **Created:** 12/15/2023 4:09 PM

PI: Kimberly Kaphingst **Submitted:** 1/4/2024

Title: Broadening the Reach, Impact, and Delivery of Genetic Services

HELP?

2. Progress of Study and Enrollment Information

Overall Study Enrollment Progress

Number of currently approved enrollments for this study as a whole: 1,920 consent cover letters sent; up to 20,000 EHR records

Number enrolled across all sites at last Continuing Review:794

Number enrolled across all University affiliated sites as of today�s date:335

Number enrolled across all sites since last Continuing Review:-459

- **Describe the progress of the study, observations of the participants studied, and reason(s) for continuing the study:**
  Utah has completed accrual. We would like to keep the protocol open for enrollment until NYU completes questionnaire 2, which is anticipated in April 2024.

- **What results (preliminary or final) have been obtained from the study? If the study is part of a multi-center trial, this should be stated and any available results provided. If there are no results that are appropriate to report to the IRB at this time, this should be stated and explained:**
  We are working on analysis of primary trial outcomes and anticipate having those ready in 2024.

  Please attach any abstracts or publications:

Name	Version	Date Created	Date Modified	Date Approved

  There are no items to display
- **Describe participant enrollment and its effect on the study design and scientific merit. If under-accrued, describe any plans for increasing enrollment during the next approval period. If a multicenter trial, also discuss enrollment at other sites.**
  As in the approved protocol and consistent with the last continuing review, we are counting enrollment as those who completed questionnaire 1 or questionnaire 2 as those are the research procedures for this study. There are some participants at the NYU site who may still complete questionnaire 2, which is why the study is left open for enrollment at this time.

  The negative number above in the enrolled at all sites comes from the discrepancy in the correction in last year's reporting of enrolled numbers and the continuation of counting only participants who complete research activities. Our accrual numbers to date are 237 at Utah and 98 at NYU, leading to the 335 reported above. In the last approved CR, we reported accrual of 307 but we are not able to indicate that in the fields above.

IRB_00115509 - CR_12/15/2023 4:09 PM **Created:** 12/15/2023 4:09 PM

PI: Kimberly Kaphingst **Submitted:** 1/4/2024

Title: Broadening the Reach, Impact, and Delivery of Genetic Services

HELP?

Progress of Sites and Enrollment

Select each site to update the enrollment information.

List of Study Controlled Locations

	Site Name	Investigator's Name	Enrollments since Last CR	Enrollments as of Today	Non English Speakers	Withdrawn
View	NYU School of Medicine	Meena Sigireddi	27	98	0	0
View	University of Utah	Kimberly Kaphingst	1	237	2	0

IRB_00115509 - CR_12/15/2023 4:09 PM **Created:** 12/15/2023 4:09 PM

PI: Kimberly Kaphingst **Submitted:** 1/4/2024

Title: Broadening the Reach, Impact, and Delivery of Genetic Services

HELP?

3. Problems & Events

1. **Provide narrative summary of any available information regarding adverse events which have occurred since the last IRB review.**
   - no events to report
2. **Have any additional risks to participation in this study been identified (from any site or other relevant source) since last approval? If reports or amendments identifying additional risks have been submitted since last approval, please mark �yes�.**
   - ○ Yes ◉ **No**

IRB_00115509 - CR_12/15/2023 4:09 PM **Created:** 12/15/2023 4:09 PM

PI: Kimberly Kaphingst **Submitted:** 1/4/2024

Title: Broadening the Reach, Impact, and Delivery of Genetic Services

HELP?

5. Amendments

A list of amendments associated with this study since the last approval date.

Name	Date Submitted	IRB #

1. **Are there any changes to be made now? (Including scientific design, procedures, participant population, or any minor changes.)**
   ◉ **Yes** ○ No

IRB_00115509 - CR_12/15/2023 4:09 PM **Created:** 12/15/2023 4:09 PM

PI: Kimberly Kaphingst **Submitted:** 1/4/2024

Title: Broadening the Reach, Impact, and Delivery of Genetic Services

HELP?

Amendment Description

1. **Type of Amendment (check all that apply):**
   Administrative changes:
2. **What changes are being made? List and number each change, grouping similar changes together.**

   1. Administrative changes to research staff

3. **Describe the reason for each of the changes described above. List and number the reasons according to the list above.**

   1. Administrative changes to reflect those who are no longer part of the study team and add a new team member.

4. **How does each change described above effect participants? List and number the effects according to the list above.**

   1. Administrative changes will have no effect on the participants.

5. **Will the modification(s), in the opinion of the local PI, increase or decrease the risk to participants?**
   Neither

   If the risk changes, provide justification:

6. **How will enrolled participants (current and past) be notified of this change?**
   Participants will not be notified

   If Other, please explain:

7. **Which approved documents are affected by these changes?**
   There are no items to display

   If Other, please list

8. **Which sections of the Update Study Application are affected by these changes?**
   1.3 and 2.1
9. **Select all study locations that are affected by these changes.**

Prior Approved Study Controlled Locations

Site Name	Investigator Name	Covered Entity
☐ NYU School of Medicine	Meena Sigireddi	Study procedures will be conducted outside a HIPAA Covered Entity at this site (HIPAA Privacy Rule does not apply)
☐ University of Utah	Kaphingst	Study procedures will be conducted within a HIPAA Covered Entity at this site (HIPAA Privacy Rule applies)

New Sites

IRB_00115509 - CR_12/15/2023 4:09 PM **Created:** 12/15/2023 4:09 PM

PI: Kimberly Kaphingst **Submitted:** 1/4/2024

Title: Broadening the Reach, Impact, and Delivery of Genetic Services

HELP?

Amendment Approvals

1. **Is this amendment related to information that meets the IRB reporting policy for unanticipated problems and non-compliance?**

○ Yes ◉ **No**

If yes, a Report Form must also be submitted and then linked to this amendment. Link the related report form to the amendment application by selecting the report form from the list below.

ID	Name	Date Submitted	Status

2. **Investigational Drug Data Form (IDDF) Changes:** ○ Yes ◉ **No**

3. **Are you adding the VA as a site?** ○ Yes ◉ **No**

IRB_00115509 - CR_12/15/2023 4:09 PM **Created:** 12/15/2023 4:09 PM

PI: Kimberly Kaphingst **Submitted:** 1/4/2024

Title: Broadening the Reach, Impact, and Delivery of Genetic Services

HELP?

6. Report Forms

Name	Date Submitted	IRB #
RP_Findings from routine audit	7/25/2023	RP_00018128

IRB_00115509 - CR_12/15/2023 4:09 PM **Created:** 12/15/2023 4:09 PM

PI: Kimberly Kaphingst **Submitted:** 1/4/2024

Title: Broadening the Reach, Impact, and Delivery of Genetic Services

HELP?

7. Documents and Attachments

Approved eProtocol Summary:

Name	Version	Date Created	Date Modified	Date Approved
ID00000007(0.01)	0.01	2/13/2023 7:43 AM	2/13/2023 7:43 AM	ID00000007
ID00000007(0.01)	0.01	2/2/2024 1:13 PM	2/2/2024 1:13 PM	ID00000007

Print View: IRB Draft Protocol Summary

Updated eProtocol Summary:

Name	Version	Date Created	Date Modified	Date Approved

There are no items to display

Approved Consent Forms:

Name	Version	Date Created	Date Modified	Date Approved
Consent Cover letter Spanish_WM.docx(0.01)	0.01	2/2/2024 1:13 PM	2/2/2024 1:13 PM	2/2/2024 1:13 PM
Consent Cover Letter _NYU (1).docx(0.01)	0.01	2/2/2024 1:13 PM	2/2/2024 1:13 PM	2/2/2024 1:13 PM

Updated Consent Forms:

Name	Version	Date Created	Date Modified	Date Approved

There are no items to display

Approved Parental Permission Forms:

Name	Version	Date Created	Date Modified	Date Approved

There are no items to display

Updated Parental Permission Forms:

Name	Version	Date Created	Date Modified	Date Approved

There are no items to display

Approved Assent Forms:

Name	Version	Date Created	Date Modified	Date Approved

There are no items to display

Updated Assent Forms:

Name	Version	Date Created	Date Modified	Date Approved

There are no items to display

Approved VA Consent Forms:

Name	Version	Date Created	Date Modified	Date Approved

There are no items to display

Updated VA Consent Forms:

Name	Version	Date Created	Date Modified	Date Approved

There are no items to display

Approved Surveys, etc.:

Name	Version	Date Created	Date Modified	Date Approved
Questionnaire #1 - No Genetic Testing 07102020.docx(0.01)	0.01	7/13/2020 2:51 PM	7/13/2020 2:51 PM	8/11/2020 3:10 PM
Questionnaire #1 - Genetic Testing 07102020.docx(0.01)	0.01	7/13/2020 2:51 PM	7/13/2020 2:51 PM	8/11/2020 3:10 PM
Questionnaire #2 - No Genetic Testing 07102020.docx(0.01)	0.01	7/13/2020 2:51 PM	7/13/2020 2:51 PM	8/11/2020 3:10 PM

Name	Version	Date Created	Date Modified	Date Approved
Questionnaire #2 - Genetic Testing 07102020.docx(0.01)	0.01	7/13/2020 2:51 PM	7/13/2020 2:51 PM	8/11/2020 3:10 PM
Questionnaire -1 - Genetic Testing 05182020_Clean_Spa_Final-Amend.docx(0.01)	0.01	9/1/2020 3:24 PM	9/1/2020 3:24 PM	9/15/2020 10:58 AM
Questionnaire - 2 - No Genetic Testing 05182020_Clean_Spa_Final-Amend.docx(0.01)	0.01	9/1/2020 3:24 PM	9/1/2020 3:24 PM	9/15/2020 10:58 AM
Questionnaire - 1 - No Genetic Testing 05182020_Clean_Spa_Final-Amend.docx(0.01)	0.01	9/1/2020 3:24 PM	9/1/2020 3:24 PM	9/15/2020 10:58 AM
Questionnaire - 2 - Genetic testing 05182020_Clean_Spa_Final-Amend.docx(0.01)	0.01	9/1/2020 3:24 PM	9/1/2020 3:24 PM	9/15/2020 10:58 AM

Updated Surveys, etc.:

Name	Version	Date Created	Date Modified	Date Approved

There are no items to display

Approved Company Protocol:

Name	Version	Date Created	Date Modified	Date Approved

There are no items to display

Updated Company Protocol:

Name	Version	Date Created	Date Modified	Date Approved

There are no items to display

Approved Investigational Brochure:

Name	Version	Date Created	Date Modified	Date Approved

There are no items to display

Updated Investigational Brochure:

Name	Version	Date Created	Date Modified	Date Approved

There are no items to display

Approved Grant Application:

Name	Version	Date Created	Date Modified	Date Approved
Grant application(0.01)	0.01	1/20/2019 3:03 PM	1/20/2019 3:03 PM	6/5/2019 2:56 PM

Updated Grant Application:

Name	Version	Date Created	Date Modified	Date Approved

There are no items to display

Approved Literature/Cited References:

Name	Version	Date Created	Date Modified	Date Approved
Background and Introduction References(0.01)	0.01	2/22/2019 8:32 AM	2/22/2019 8:32 AM	6/5/2019 2:56 PM

Updated Literature/Cited References:

Name Version Date Created Date Modified Date Approved

There are no items to display

Current PI Scholarly Record (CV/Resume):

Name	Version	Date Created	Date Modified	Date Approved
Kaphingst CV(0.04)	0.04	4/2/2015 12:18 PM	8/27/2018 3:25 PM	

Updated PI Scholarly Record (CV/Resume):

Name	Version	Date Created	Date Modified	Date Approved
Kaphingst CV(0.04)	0.04	4/2/2015 12:18 PM	8/27/2018 3:25 PM	

Current Faculty Sponsor Scholarly Record (CV/Resume):

Name Version Date Created Date Modified Date Approved

There are no items to display

Updated Faculty Sponsor Scholarly Record (CV/Resume):

Name Version Date Created Date Modified Date Approved

There are no items to display

Approved Other Stamped Documents:

Name Version Date Created Date Modified Date Approved

There are no items to display

Updated Other Stamped Documents:

Name Version Date Created Date Modified Date Approved

There are no items to display

Approved Recruitment Materials, Advertisements, etc.:

Name	Version	Date Created	Date Modified	Date Approved
Q1 invitation-No testing.docx(0.01)	0.01	7/14/2020 3:54 PM	7/14/2020 3:54 PM	8/11/2020 3:10 PM
Q1 invitation-Testing.docx(0.01)	0.01	7/14/2020 3:54 PM	7/14/2020 3:54 PM	8/11/2020 3:10 PM
Q2 Inivitation-No testing.docx(0.01)	0.01	7/14/2020 3:54 PM	7/14/2020 3:54 PM	8/11/2020 3:10 PM
Q2 Invitation-Testing.docx(0.01)	0.01	7/14/2020 3:54 PM	7/14/2020 3:54 PM	8/11/2020 3:10 PM
Q1 mail coverletter-No testing_Utah.docx(0.01)	0.01	7/15/2020 2:45 PM	7/15/2020 2:45 PM	8/11/2020 3:10 PM

Name	Version	Date Created	Date Modified	Date Approved
Q1 mail coverletter-Testing_Utah.docx(0.01)	0.01	7/15/2020 2:45 PM	7/15/2020 2:45 PM	8/11/2020 3:10 PM
Q2 mail coverletter-Testing_Utah.docx(0.01)	0.01	7/15/2020 2:45 PM	7/15/2020 2:45 PM	8/11/2020 3:10 PM
Q2 mail coverletter-No Testing_Utah.docx(0.01)	0.01	7/15/2020 2:58 PM	7/15/2020 2:58 PM	8/11/2020 3:10 PM
Q1 mail coverletter-No testing_Utah_Spa_Final.docx(0.01)	0.01	9/1/2020 3:25 PM	9/1/2020 3:25 PM	9/15/2020 10:58 AM
Q2 Invitation-Testing_Spa_Final.docx(0.01)	0.01	9/1/2020 3:25 PM	9/1/2020 3:25 PM	9/15/2020 10:58 AM
Q1 invitation-Testing_Spa_Final.docx(0.01)	0.01	9/1/2020 3:25 PM	9/1/2020 3:25 PM	9/15/2020 10:58 AM
Q2 mail coverletter-Testing_Utah_Spa_Final.docx(0.01)	0.01	9/1/2020 3:25 PM	9/1/2020 3:25 PM	9/15/2020 10:58 AM
Q1 invitation-No testing_Spa_Final.docx(0.01)	0.01	9/1/2020 3:25 PM	9/1/2020 3:25 PM	9/15/2020 10:58 AM
Q2 mail coverletter-No Testing_Utah_Spa_Final.docx(0.01)	0.01	9/1/2020 3:25 PM	9/1/2020 3:25 PM	9/15/2020 10:58 AM
Q1 mail coverletter-Testing_Utah_Spa_Final.docx(0.01)	0.01	9/1/2020 3:25 PM	9/1/2020 3:25 PM	9/15/2020 10:58 AM
Q2 Inivitation-No testing_Spa_Final.docx(0.01)	0.01	9/1/2020 3:25 PM	9/1/2020 3:25 PM	9/15/2020 10:58 AM
20210812_MS_CLEAN_Q1 Mail Coverletter_Testing_NYU.docx(0.01)	0.01	8/16/2021 8:15 AM	8/16/2021 8:15 AM	8/25/2021 10:25 AM
20210812_MS_CLEAN_Q2 Mail Coverletter_Testing_NYU.docx(0.01)	0.01	8/16/2021 8:15 AM	8/16/2021 8:15 AM	8/25/2021 10:25 AM
20210812_MS_CLEAN_Q1 Mail Coverletter_Testing_NYU_Spa_Final.docx(0.01)	0.01	8/16/2021 8:15 AM	8/16/2021 8:15 AM	8/25/2021 10:25 AM
20210812_MS_CLEAN_Q2 Mail Coverletter_Testing_NYU_Spa_Final.docx(0.01)	0.01	8/16/2021 8:15 AM	8/16/2021 8:15 AM	8/25/2021 10:25 AM
20210812_MS_CLEAN_Q1 Mail Coverletter_No testing_NYU.docx(0.01)	0.01	8/16/2021 8:16 AM	8/16/2021 8:16 AM	8/25/2021 10:25 AM
20210812_CLEAN_Q2 Mail Coverletter_No testing_NYU.docx(0.01)	0.01	8/16/2021 8:16 AM	8/16/2021 8:16 AM	8/25/2021 10:25 AM
20210812_MS_CLEAN_Q1 Mail Coverletter_No testing_NYU_Spa_Final.docx(0.01)	0.01	8/16/2021 8:16 AM	8/16/2021 8:16 AM	8/25/2021 10:25 AM
20210812_MS_CLEAN_Q2 Mail Coverletter_No testing_NYU_Spa_Final.docx(0.01)	0.01	8/16/2021 8:16 AM	8/16/2021 8:16 AM	8/25/2021 10:25 AM

Updated Recruitment Materials, Advertisements, etc.:

Name Version Date Created Date Modified Date Approved

There are no items to display

Approved Other Documents:

Name	Version	Date Created	Date Modified	Date Approved
Translation certificate for Consent Coverletter(0.01)	0.01	9/1/2020 3:27 PM	9/1/2020 3:27 PM	9/15/2020 10:58 AM
Translation Certificate for Questionnaires and Recruitment Letters(0.01)	0.01	9/1/2020 3:28 PM	9/1/2020 3:28 PM	9/15/2020 10:58 AM
Sigireddi CITI Biomed Research(0.01)	0.01	8/23/2021 10:01 AM	8/23/2021 10:01 AM	8/25/2021 10:25 AM
Sigireddi CITI Clinical Practice(0.01)	0.01	8/23/2021 10:02 AM	8/23/2021 10:02 AM	8/25/2021 10:25 AM
Sigireddi Coi(0.01)	0.01	8/23/2021 10:02 AM	8/23/2021 10:02 AM	8/25/2021 10:25 AM
Sigireddi CV(0.01)	0.01	8/23/2021 10:03 AM	8/23/2021 10:03 AM	8/25/2021 10:25 AM

Updated Other Documents:

Name	Version	Date Created	Date Modified	Date Approved

There are no items to display

IRB_00115509 - CR_12/15/2023 4:09 PM **Created:** 12/15/2023 4:09 PM

PI: Kimberly Kaphingst **Submitted:** 1/4/2024

Title: Broadening the Reach, Impact, and Delivery of Genetic Services

HELP?

8. Instructions and Finish

1. To view errors in this application, select the "Validate" option at the top-left of the page. If you have errors on your application, you won't be able to submit it to the IRB.

Changes to the Update Study Application

2. Be sure to make all proposed changes to the Update Study portion of the application by selecting the "Update Study" button located on the left side of the amendment or continuing review workspace, which will be available once you select the "Finish" button at the top or bottom of this page.
3. To attach updated or new documents with this application, you may access the Documents and Attachments page in the Update Study application.
4. If you are proposing changes to any ancillary applications (i.e. RDRC-HUS or RGE), you must access these applications through the Update Study application on the Ancillary Applications page. All changes to ancillary applications must be approved by the corresponding committee prior to IRB approval of the amendment.

Submitting the Completed Continuing Review Application

5. Selecting the �Finish� button alone will NOT submit the application to the IRB. You MUST also select the "Submit" option on the workspace after you've selected the "Finish" button. Only the PI can submit the application to the IRB.
6. If your study has a faculty sponsor: Once the PI submits the application, it will be sent to the faculty sponsor for final approval. The IRB cannot review the study until the faculty sponsor submits the application to the IRB.

IRB_00115509 - CR_12/15/2023 4:09 PM **Created:** 12/15/2023 4:09 PM

PI: Kimberly Kaphingst **Submitted:** 1/4/2024

Title: Broadening the Reach, Impact, and Delivery of Genetic Services

Study Location Questions

NYU School of Medicine

Site Status

What is the enrollment status for this site?
Open to enrollment of new participants

1,920 consent cover letters sent; up to 20,000 EHR records **Number of currently approved enrollments for this study as a whole**

960**Number of currently approved enrollments for this study location**

Enrollment since the beginning of the study at this site:

98**Total number enrolled as of today�s date at this site**

From the beginning of the study, how many non-English speakers have been enrolled?
0

From the beginning of the study, how many participants signed consent forms but did not end up participating in the study?
0

Enrollment since the last continuing review approval at this site:

71**Reported enrollment during the last continuing review**

27**Number enrolled since the last continuing review**

Problems and Events

Have any unanticipated problems or adverse events involving risks to participants occured since the last IRB approval?
○ Yes ● **No**

Have there been any complaints about the research since the last IRB review?
○ Yes ● **No**

Local HRPP Reporting

Have any significant financial conflicts of interest for study personnel been identified since the last approval that have not been reported to the IRB?
○ Yes ● **No**

Have there been any changes to human research protection reviews or approvals from your institution since last approval that have not been reported to the IRB.
○ Yes ● **No**

Title: Broadening the Reach, Impact, and Delivery of Genetic Services

Study Location Questions

University of Utah

Site Status

What is the enrollment status for this site?
Permanently closed to enrollment of new participants

Will consent forms be used to re-consent participants after this application is approved?
○ Yes ◉ **No**

Select all of the remaining research activities that will be performed at this site after approval of this continuing review:
Data and/or biospecimen analysis

Indicate the type(s) of data/biospecimens are being analyzed:
Identifiable private data/biospecimens
A HIPAA Limited Data set

1,920 consent cover letters sent; up to 20,000 EHR records **Number of currently approved enrollments for this study as a whole**

960**Number of currently approved enrollments for this study location**

Enrollment since the beginning of the study at this site:

237**Total number enrolled as of today�s date at this site**

From the beginning of the study, how many non-English speakers have been enrolled?
2

Which non-English languages were used in the enrollment process since the last review?

Yes **Spanish**

How many Spanish fully translated long forms were used? 2 How many Spanish short forms were used? 0

☐ **Mandarin**

- [ ] **Nepali**
- [ ] **Vietnamese**
- [ ] **Russian**
- [ ] **Other**

From the beginning of the study, how many participants signed consent forms but did not end up participating in the study?
0

Enrollment since the last continuing review approval at this site:

236**Reported enrollment during the last continuing review**

1**Number enrolled since the last continuing review**

Problems and Events

Have any unanticipated problems or adverse events involving risks to participants occured since the last IRB approval?
○ Yes ◉ **No**

Have there been any complaints about the research since the last IRB review?
○ Yes ◉ **No**
