# Supplementary material for: Uptake of Cancer Genetic Services for Chatbot vs Standard-of-Care Delivery Models: The BRIDGE Randomized Clinical Trial
Source: JAMA Netw Open. 2024 Sep 9;7(9):e2432143. doi: 10.1001/jamanetworkopen.2024.32143 (PMC11385050; doi:10.1001/jamanetworkopen.2024.32143)
Supplement: Supplement 2. — eFigure. BRIDGE Pretest Genetics Education Chat for the University of Utah Health Site [file jamanetwopen-e2432143-s002.pdf]

Supplemental Online Content

Kaphingst KA, Kohlmann WK, Lorenz Chambers R, et al; BRIDGE Research Team. Uptake of cancer genetic services for chatbot vs standard-of-care delivery models the BRIDGE randomized clinical trial. *JAMA Netw Open*. 2024;7(9):e2432143. doi:10.1001/jamanetworkopen.2024.32143

eFigure. BRIDGE Pretest Genetics Education Chat for the University of Utah Health Site

This supplemental material has been provided by the authors to give readers additional information about their work.

© 2024 Kaphingst KA et al. *JAMA Network Open.*

eFigure. BRIDGE pretest genetics education chat for University of Utah Health site

**Patient receives this pretest education chat if identified by BRIDGE as meeting criteria for testing.**
Gia: Hi, {patientName}.<br><br>Gia: I'm GIA, your automated Genetic Information Assistant.<br><br>Gia: On behalf of the University of Utah healthcare team, I'm reaching out to patients who might benefit from genetic testing.<br><br>Patient: Hi Gia<br><br>Gia: Before we get started, here's a quick message from Wendy at University of Utah genetics.<br><br>*** INTRO VIDEO ***<br><br>Patient: Let's continue<br><br>Gia: Based on the family health history in your medical record, it looks like you meet criteria for testing of genes related to certain cancers.<br><br>Gia: The healthcare team at University of Utah recommends having this genetic testing to provide more information about your cancer risk and to help plan your care.<br><br>Gia: It can also be important for your family members, including children if you have them.<br><br>Patient: I see<br><br>Gia: In this chat I'll ask you some basic information about your family, and provide information about why testing is important, how it is done, and what results are possible.<br><br>Gia: At the end, I can answer any additional questions you have.<br><br>Patient: Sounds good<br><br>Gia: At any time during our conversation you can click the menu button (☰) to access additional options such as increasing font size or read speed.<br><br>Gia: You can also click on any of your responses to change them.<br><br>Gia: When we're done chatting, a team member will contact you to help with next steps.

© 2024 Kaphingst KA et al. *JAMA Network Open.*

Patient: Let's continue	Patient: I'll come back later<br><br>Gia: Ok, when you come back we'll continue where we left off.<br><br>Gia: The sooner you complete our chat, the sooner we can take next steps.<br><br>Patient: I'm ready to continue now

Gia: Great!

Gia: Let's start with the questions about your family.

Gia: I know everyone's family is different and that a blood relationship is not always what defines family.

Gia: But because we're talking about genetics, I will only be asking for information about your biological (or blood) relatives.

Gia: First, do you have any siblings?

Patient: Yes / No / I'm not sure

Gia: How many brothers do you have? (includes full and half brothers)

Patient: *Types in*

Gia: How many sisters do you have? (includes full and half sisters)

Patient: *Types in*

Gia: And do you have any children?

Patient: Yes / No

Gia: How many sons do you have?

Patient: Chooses from options 0 - 10

Gia: How many daughters do you have?

© 2024 Kaphingst KA et al. *JAMA Network Open.*

Patient: Chooses from options 0 - 10

Gia: Thank you for answering those questions.

Gia: Now let's review some basics of genetics and cancer.

Patient: Sounds good

Gia: Here's a diagram that I think is helpful.

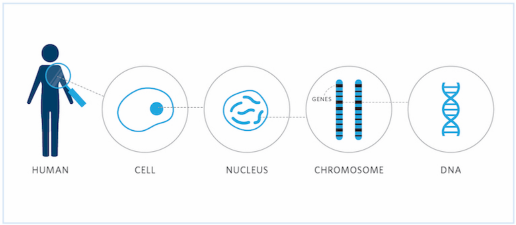

Gia: Your body is made up of trillions of cells, like the one in the diagram and each cell contains structures called chromosomes.

Gia: Chromosomes come in pairs, represented by the 2 lines, one from your mother and one from your father.

Gia: The chromosomes contain genes and each gene does a specific job such as controlling hair or eye color for example.

Gia: Sometimes a gene has a change in the genetic code. A change that keeps a gene from doing its job correctly is called a mutation.

Patient: I see / This is sounding familiar now

Gia: Some genes have the job of preventing cancer.

Patient: I have a good understanding of genetics

Gia: Great. As you may know, your cells contain chromosomes which come in pairs.

Gia: The chromosomes contain genes and each gene does a specific job such as controlling hair or eye color.

Gia: Sometimes a gene has a change in the genetic code. A change that keeps a gene from doing its job correctly is called a mutation.

Gia: If a person has a mutation in a gene whose job is to prevent cancer then their risk of cancer can be increased.

Gia: It doesn't mean that a person will definitely get cancer and their degree of risk depends on many factors.

Patient: Ok / Yes, we're on the same page

© 2024 Kaphingst KA et al. *JAMA Network Open.*

Gia: If a person has a mutation in a gene that is supposed to be preventing cancer, then their risk of cancer can be increased.<br><br>Gia: It doesn’t mean that person will definitely get cancer and their degree of risk depends on many factors.<br><br>Patient: Ok / Interesting!	

Gia: Cancer can be inherited, or it can occur randomly.

Gia: Cancer that is caused by a gene mutation is “hereditary”, meaning that it is passed down within families.

Gia: Other cancer is random, or “sporadic,” which means that it’s caused by things like aging, lifestyle, and environment.

Patient: I understand	Patient: Can you explain this in another way?<br><br>Gia: Yes! Here's a short video that explains it nicely.<br><br>[Video]<br><br>Patient: Thanks, that was helpful / Let’s continue

Gia: Everyone has some level of cancer risk. Most people have what is known as "average" or "population" risk.

Gia: People with a close family member with cancer, such as a parent or sibling, generally have an increased risk for developing that type of cancer.

Gia: Those that test positive for a gene mutation related to cancer may have an even higher risk.

Patient: I see	Patient: Can you give me an example?<br><br>Gia: Of course!

© 2024 Kaphingst KA et al. *JAMA Network Open.*

<table>
<tr><td rowspan="3"></td><td colspan="4">Gia: Here's an illustration of lifetime breast cancer risk for a woman. ***IMAGE***

Gia: The average lifetime risk of breast cancer is 10%.

Gia: Having a mother or sister with breast cancer increases the risk to 15 - 25%.

Gia: And those with gene mutations in the gene called BRCA1 have a 50 - 70% risk for breast cancer.</td></tr>
<tr><td rowspan="2">Patient: Thanks, that helps</td><td colspan="2">Patient: Why is there a range of percentages?

Gia: The exact risk depends on many factors including the specific gene mutation, if there is one, and the details of the family history.

Gia: You can talk to a genetic counselor to help you understand your personal risk.</td><td rowspan="2">Patient: Can I see more examples?

Loop through offering other examples below</td></tr>
<tr><td>Patient: Ok</td><td>Patient: Can I see more examples?

Loop through offering other examples below</td></tr>
</table>

Ovarian cancer

Gia: Here's an illustration of lifetime ovarian cancer risk for a woman. ***IMAGE***<br><br>Gia: The average lifetime risk of ovarian cancer is 1%.<br><br>Gia: Those with family history have a risk of 3 - 5%.<br><br>Gia: And those with a high-risk gene mutation have a risk of 20 - 50%.

© 2024 Kaphingst KA et al. *JAMA Network Open.*

Colon cancer

Gia: Here's an illustration of lifetime colon cancer risk. ***IMAGE***

Gia: The average lifetime risk of colon cancer is 5%.

Gia: Those with family history have a risk of 8 - 10%.

Gia: And those with a high-risk gene mutation have a risk of 20 - 50%.

Pancreatic cancer

Gia: Here's an illustration of lifetime pancreatic cancer risk. ***IMAGE***

Gia: The average lifetime risk of pancreatic cancer is 1%.

Gia: Those with family history have a risk of 2 - 3%.

Gia: And those with a high-risk gene mutation have a risk of 5 - 20%.

Gia: There are specific things we look for in a family's cancer history to figure out whether the cancer is likely to be hereditary.

Gia: You may have been identified as eligible for genetic testing because we found one or more of these things in your medical record.

Patient: What kind of things do you look for?<br><br>Gia: Some of the things we look for are:<br><br>Gia: Having cancer at a young age (usually younger than 50)	Patient: Ok, Gia

© 2024 Kaphingst KA et al. *JAMA Network Open.*

Gia: Having multiple family members with cancers that are known to share a common genetic link<br><br>Gia: Some rare cancer types such as ovarian cancer, men with breast cancer, or pancreatic cancer<br><br>Gia: Jewish ancestry in combination with certain cancers<br><br>Patient: Got it	

Gia: The genetic testing we are offering you looks at many genes that are related to hereditary cancer.

Gia: Each gene is unique in terms of which cancer(s) it relates to, the level of risk it causes, and how it impacts care.

Patient: I see	Patient: Which genes are included?<br><br>Gia: We test for mutations in genes you may have heard of like *BRCA1* and *BRCA2*.<br><br>Gia: And also, for many other genes that you likely are not familiar with.<br><br>Gia: If you have questions about specific genes, we can answer those when we talk to you on the phone.<br><br>Patient: Got it

Gia: If we find a mutation in one of these genes, additional or more frequent cancer screening may be recommended to help detect cancer early.

Gia: For some types of cancer there might be other options that can help lower cancer risk.

Patient: Ok / What kind of options? / Do I have to get tested?

© 2024 Kaphingst KA et al. *JAMA Network Open.*

Patient: Do I have to get tested?<br><br>Gia: Testing is your choice, but this information could help your healthcare team plan what cancer screening is best for you.<br><br>Gia: If you decide to get tested, you will be well supported.<br><br>Gia: If you decide not to get genetic testing, we can only give recommendations for cancer screening based on your personal and family history.<br><br>Gia: At the end of this chat you can decide if you want testing and can talk to a genetics professional to get more information.<br><br>Patient: Ok	Patient: What kind of options?<br><br>Gia: It depends on your specific situation.<br><br>Gia: For example, if you are at high risk of developing breast cancer, you may be offered a mammogram and breast MRI every year to screen you more closely.<br><br>Gia: Surgical options may be considered with some mutations.<br><br>Patient: Ok / Do I have to have testing?

Gia: There are 3 types of results that we may find when we look at your genes. Let me describe them for you:

1) **Positive**: A gene mutation was found that increases the chances of developing certain cancers. Laboratories may call a mutation a "pathogenic variant" on a test report.

2) **Negative**: No gene mutations that increase the chance of developing cancer were found in any of the genes tested.

3) **Variant of Unknown Significance (VUS)**: A variation was found in one of your genes, but the variation has not been proven to increase the risk of cancer. This is not a positive result and should not be used to change your medical management.

Patient: More on positive result	Patient: More on negative result	Patient: More on VUS result	Patient: Let's move on

© 2024 Kaphingst KA et al. *JAMA Network Open.*

Gia: If you have a positive result, your doctor or a genetic counselor will walk you through the next steps.<br><br>Gia: It's important to know that if you test positive, it does not mean you will definitely develop cancer.	Gia: A negative result would help reassure you that you do not have a gene mutation causing a higher risk for cancer.<br><br>Gia: However, you may still have an increased risk for cancer based on your family history.<br><br>Gia: The genetic counseling team can help a relative have genetic testing.	Gia: All people have variations in their genes. This is part of what makes each person unique.<br><br>Gia: Sometimes genetic testing identifies variations and we can't say for sure yet if they are linked to cancer.<br><br>Gia: As we learn more, most of these variants are found to be normal (not associated with cancer or other disease).<br><br>Gia: If we find you have a VUS we will keep track of it and notify you when we learn more about it.<br><br>Patient: Got it / 👍<br><br>Gia: Even with a VUS result, you may still have an increased risk for cancer based on your family history.<br><br>Gia: A genetic counselor will review your family history and let you know what cancer screening is recommended.	

© 2024 Kaphingst KA et al. *JAMA Network Open.*

<table>
<tr><td rowspan="2">Patient: Let’s move on / Ok</td><td>Patient: Let’s move on / Ok</td><td></td></tr>
<tr><td colspan="2">If didn’t already cover more info on positive result (otherwise skip this section)

Gia: Sounds good

Gia: If you have a positive result, your doctor or a genetic counselor will walk you through the next steps.

Gia: It’s important to know that if you test positive, it does not mean you will definitely develop cancer.

Patient: Ok</td></tr>
<tr><td colspan="3">Gia: If you are found to have a mutation, it will be important to share this information with family members.

Gia: Close relatives like children, siblings and parents have a 1 in 2 (or 50%) chance of having the same mutation and other relatives may also have increased risk.

Gia: If needed, we can help your relatives get testing.</td></tr>
<tr><td colspan="3">Patient: Got it / How is privacy protected?

Gia: Only you and your healthcare providers will have access to your results.

Gia: Your report will go into your medical record so those involved in your care can review it as needed.

Gia: It is up to you whether you choose to share your genetic test results with others outside of the healthcare system.

Gia: However, as we have discussed it is often very helpful to share your genetic test results with family members.</td></tr>
</table>

© 2024 Kaphingst KA et al. *JAMA Network Open*.

<table>
<tr><td colspan="3">Patient: Ok

Gia: Also, you are protected by GINA, the Genetic Information Nondiscrimination Act.

Gia: GINA provides specific protections related to health insurance and employment. For more complete information click **here**.

Patient: Ok / Thank you</td></tr>
<tr><td colspan="3">Gia: If you’re interested in genetic testing for cancer risk, the genetics team will give you a call to discuss next steps.

Gia: We’ll work with you on insurance coverage. Out of pocket cost is usually $100 or less.

Gia: Before I ask if you're interested in testing, do you have any additional questions?

Clear AI

Gia: What do you think? Would you like to move forward with testing?</td></tr>
<tr><td>Patient: Yes

Gia: Great. When we call you, we’ll go over instructions for providing a sample for the testing and to answer any additional questions about insurance.

Gia: A kit can be sent to your home in the next 72 hours, and results will be available in 3 to 4 weeks after you send the sample.

Gia: Here’s some more information on next steps from Wendy at University of Utah genetics.</td><td>Patient: No

Gia: Ok. Someone on the team might reach out to you to make sure you got all the information you need.</td><td>Patient: I’m not sure yet

Gia: Ok, I’ll pass that information on to the team.

Gia: Someone will reach out to you about your questions.</td></tr>
</table>

© 2024 Kaphingst KA et al. *JAMA Network Open.*

Gia: Thank you {patient name} for taking the time to chat with me.

Gia: Have a great day. Goodbye!

end chat

© 2024 Kaphingst KA et al. *JAMA Network Open.*
