# Supplementary material for: Uptake of Cancer Genetic Services for Chatbot vs Standard-of-Care Delivery Models: The BRIDGE Randomized Clinical Trial
Source: JAMA Netw Open. 2024 Sep 9;7(9):e2432143. doi: 10.1001/jamanetworkopen.2024.32143 (PMC11385050; doi:10.1001/jamanetworkopen.2024.32143)
Supplement: Supplement 3. — Data Sharing Statement [file jamanetwopen-e2432143-s003.pdf]

Data Sharing Statement

Kaphingst. Uptake of Cancer Genetic Services for Chatbot vs Standard-of-Care Delivery Models. *JAMA Netw Open*. Published September 09, 2024.
doi:10.1001/jamanetworkopen.2024.32143

Data
Data available: Yes
Data types: Deidentified participant data
How to access data: An open-source version of the chatbot is available through the GARDE platform, which is funded by the Informatics Technology for Cancer (ITCR) program of the NCI. GARDE contains a chatbot authoring tool that allows researchers to create scripted chatbots. The authoring tool also provides an open repository of chatbots, including the BRIDGE chatbot, that researchers can use as a starting point and adapt it to their needs. More recently, the authoring tool has been enhanced to support hybrid chatbots that include scripted information and ability to ask questions that are redirected to large language models (LLMs) such as GPT4. Access to the GARDE authoring tool can be granted upon request to corresponding author. The trial data presented is available through the Inter-university Consortium for Political and Social Research (ICPSR) data repository at www.icpsr.umich.edu
When available: With publication

Supporting Documents
Document types: None

Additional Information
Who can access the data: Those requesting data from ICPSR
Types of analyses: No specified purpose
Mechanisms of data availability: ICPSR request process
